# Supplementary material for: Use of strategies to improve retention in primary care randomised trials: a qualitative study with in-depth interviews
Source: BMJ Open. 2014 Jan 23;4(1):e003835. doi: 10.1136/bmjopen-2013-003835 (PMC3902408; doi:10.1136/bmjopen-2013-003835)
Supplement: Author's manuscript [file bmjopen-2013-003835.draft_revisions.pdf]

Use of strategies to improve retention in primary care randomised trials: a qualitative study with in-depth interviews

Journal:	*BMJ Open*
Manuscript ID:	bmjopen-2013-003835
Article Type:	Research
Date Submitted by the Author:	16-Aug-2013

Complete List of Authors:	Brueton, Valerie; MRC Clinical Trials Unit, Meta-analysis Group<br>Stevenson, Fiona; University College London, Research Department of Primary Care and Population Health<br>Vale, Claire; MRC Clinical Trials Unit, Meta-analysis Group<br>Stenning, Sally; MRC Clinical Trials Unit, Cancer and non infections Group<br>Tierney, Jayne; MRC Clinical Trials Unit, Meta-analysis Group<br>Harding, Seeromanie; MRC Social and Public Health Sciences Unit,<br>Nazareth, Irwin; UCL, Primary Care and Population Health<br>Meredith, Sarah; MRC Clinical Trials Unit,<br>Rait, Greta; University College London, Primary Care and Population Health

**Primary Subject Heading**:	Research methods
Secondary Subject Heading:	General practice / Family practice, Qualitative research
Keywords:	Retention strategies, Randomised trials, PRIMARY CARE

SCHOLARONE™ Manuscripts

For peer review only

Use of strategies to improve retention in primary care randomised trials: a qualitative study with in-depth interviews

Brueton V

Research Scientist, Meta-analysis Group
MRC Clinical Trials Unit
Aviation House
125 Kingsway
London WC2B 6NH
e-mail: vcb@ctu.mrc.ac.uk

Stevenson F

Senior Lecturer
UCL Research Department of Primary care and Population Health
Royal Free and University College Medical School
Rowland Hill Street
London
NW3 2PF

Vale CL

Senior Scientist Meta-analysis Group
MRC Clinical Trials Unit
Aviation House
125 Kingsway
London WC2B 6NH

Stenning SP

Senior Statistician
MRC Clinical Trials Unit
Aviation House
125 Kingsway
London WC2B 6NH

Tierney JF

Senior Scientist Meta-analysis Group
MRC Clinical Trials Unit
Aviation House
125 Kingsway
London WC2B 6NH

Harding S

Program leader: Ethnicity and Health

For peer review only

BMJ Open qualitative paper submitted 15.08.2013

MRC Social & Public Health Sciences Unit
4 Lilybank Gardens
Glasgow G12 8RZ

Nazareth I

Professor of Primary Care
PRIMENT Clinical Trials Unit
Research Department of Primary care and Population Health
UCL Medical School
Rowland Hill Street
London
NW3 2PF

Meredith S

Deputy Director
MRC Clinical Trials Unit
Aviation House
125 Kingsway
London WC2B 6NH

Rait G

Clinical Senior Lecturer
PRIMENT Clinical Trials Unit
Research Department of Primary care and Population Health
UCL Medical School
Rowland Hill Street
London
NW3 2PF

For peer review only

BMJ Open qualitative paper submitted 15.08.2013

Abstract

Objective

To explore the use of strategies to improve retention identified by a Cochrane systematic review in UK primary care randomised trials (RCTs).

Design

Qualitative in-depth interviews and thematic analysis.

Participants

Twenty nine UK primary care chief and principal investigators, trial managers and research nurses.

Methods

In-depth face to face interviews.

Results

Primary care researchers used various incentives and communication strategies to improve retention in trials, but were unsure of their effect. Small monetary incentives were used to increase response to postal questionnaires. Non-monetary incentives were used although there was scepticism about the impact of these on retention. Nurses routinely used telephone communication to encourage participants to return for trial follow-up. Trial managers routinely used 1st class post to increase questionnaire response. Shorter questionnaires were sent to non-responders, and suggestions were offered for improving questionnaire design.

Interviewees thought that an open trial design could lead to biased results and were generally negative about the use of behavioural strategies to improve retention.

There was consensus among the interviewees that effective communication and rapport with participants, participant altruism, flexibility of trial personnel and appointment schedules, and trial information improve retention. Participant age, disease type and the work environment at trial sites were thought to contribute to loss to follow-up.

Conclusions

For peer review only

Integration of this qualitative study with the Cochrane review gave an in-depth explanation of the review results in the context of primary care trials. The findings provide an understanding of the complexity of retention in primary care trials and the factors that may facilitate or limit the implementation of effective strategies. The use of small monetary incentives to improve questionnaire response is effective and acceptable. A reconsideration of the use of non-monetary incentives, and some communication strategies is needed. A similar exploration of barriers and facilitators to retention in other research contexts would help identify ways to improve retention in trials conducted in other research contexts.

For peer review only

Introduction

Retention in primary care randomised controlled trials (RCTs) conducted across different disease areas and communities can be challenging. A Cochrane systematic review of strategies to improve retention in randomised trials demonstrated that adding a monetary incentive and offering higher valued monetary incentives increased postal and electronic questionnaire response[1]. Questionnaire response was also increased by recorded delivery of questionnaires, a "package" of postal communication strategies known as the Total Design Method and an open trial design although these are based on the results of single trials. The evidence of an effect for shorter questionnaires and questionnaires relevant to the disease / condition was less clear. Also, there was no good evidence that the following strategies improved retention: adding or offering a non-monetary incentive, communication strategies, behavioural motivational strategies, new questionnaire formats and case management.

Reasons for loss to follow-up can include a change in the participants' location, withdrawal from treatment and / or loss of commitment to the trial, for example due to complicated treatment / medicine regimens[2]. Primary care trials often require participants to return to sites for clinical outcome measurement for example, assessment of disease progression[3,4]. However, in the systematic review, there were few evaluations of strategies to increase this type of retention and only six of the 38 retention trials included in the systematic review were embedded in UK primary care host trials[1]. Rather the included trials targeted improving response to postal questionnaires[5-8] Moreover, there may be barriers to using strategies to improve retention and in particular those identified by the systematic review. We therefore designed a qualitative study to explore the use of strategies to improve retention and also to identify the factors thought to contribute to retention in the context of primary care RCTs. The aim was to gain insights into how best to improve retention in primary care RCTs and trials in general.

Methods

Study population and sample

In-depth interviews were conducted with principal / chief investigators (PIs), trial managers (TMs) and research nurses (RNs) purposively sampled from primary care trials published between 2000-2010, across different disease areas, research units, time intervals, and levels of loss to follow-up (Tables 1 and 2). The trials were identified from either the MRC General Practice Research Framework (GPRF) database of clinical research; websites of UK primary

For peer review only - http://bmjopen.bmj.com/site/about/guidelines.xhtml

care research units or from hand searches of trials published between 2009-2010 in *Lancet, British Medical Journal, Family Practice* and *British Journal of General Practice*. Principal and chief investigators identified by word of mouth were also invited to participate. Most TMs were recruited through the Trial Managers Network[9], and RNs through a primary care research network. In the sampling frame trial publications were stratified by loss to follow-up of <20% and >20%, and the date of publication i.e. pre or post 2005. Potential interviewees were identified from the list of authors associated with each trial in the sampling frame and through trial records held at the MRC GPRF.

Data collection

Ethics approval was granted by University College London Research Ethics committee (application no 2342/002). Potential interviewees were sent an information sheet and recruited via email. After informed consent, all in-depth interviews were recorded digitally. The interviews explored the use in primary care trials of the six types of strategies identified by the systematic review of strategies to improve retention in randomised trials (Interview schedule Appendix 1). The six types of strategies identified were: incentives, different means of communication, new questionnaire formats, behavioural, case management and methodological strategies[1]. Interviewees were asked about the factors that they thought contributed to retention and loss to follow-up in primary care trials. Interviewees were also asked specifically about the role of the ethics approval process on the use of monetary incentives to improve retention in primary care trials. The interviews were approximately one hour long and were conducted by one author (VB) in a place convenient to the interviewee.

Data analysis

We conducted a thematic content analysis of the interview transcripts. These were anonymised and analysed by one author (VB) and, all transcripts were reviewed and validated by at least two authors (CV, FS, GR, VB). The textual data was managed using Atlas ti 6. Each transcript was read and re read and themes identified and recorded. Codes were developed by one author (VB) verified by another author (FS) and then used to label textual data for retrieval from Atlas ti 6. Textual data in each transcript relating to retention strategies identified by the systematic review and any new strategies was subsequently retrieved using Atlas ti 6. Coded content was compared across each of the interview transcripts within the three groups interviewed, in order to identify the use, and advantages and disadvantages of

For peer review only - http://bmjopen.bmj.com/site/about/guidelines.xhtml

each strategy. Any factors thought by the interviewees to contribute to either retention or loss to follow-up were also coded and retrieved for analysis[10,11]. Relevant quotes, representing the interviewee's views, were selected to illustrate the results. Interviews were conducted between August 2010 and May 2011 until no new content or themes emerged.

Results

Ten PIs, ten TMs and nine RNs were interviewed across 37 primary care trials. Interviewees were from the fields of nutrition, health promotion, neurology, gynaecology, mental health, and musculoskeletal, ear nose and throat (ENT), respiratory, and endocrine medicine, as well as minor medical conditions. Collectively their experiences included trial design, implementation, coordination and data collection at GP practice sites by post, face to face and electronic methods. Trial publications reported loss to follow-up rates between 5% - 39%. Twenty interviewees were from trials with <20% loss to follow-up.

Nine PIs were registered GPs with academic roles, and one was a non-clinical senior academic. They were responsible for the design and implementation of trials, including research governance and ethics approval applications. The TMs were based in academic research units. They managed trial data collection via telephone, post, email and SMS text message; they coordinated and monitored data centrally and communicated with trial sites. Six RNs were based at GP practice sites where they coordinated clinical research. They collected biomedical and clinical data by telephone and face-to-face interview, and also communicated with the coordinating centre and with PI clinicians at sites. Three RNs were based at a primary care research network coordinating centre; where they answered clinical data collection queries and monitored data quality. See Tables 1 and 2 for the characteristics of the study sample.

The use of retention strategies identified by the Cochrane review

Incentive strategies

Monetary incentives and offers of monetary incentives were mentioned spontaneously by most PIs and TMs and were often used by interviewees to try to increase questionnaire response. Over half of the PIs mentioned using these. The value of incentives used was between £5 and £20. Most of those interviewed thought it was acceptable to use incentives to

For peer review only - http://bmjopen.bmj.com/site/about/guidelines.xhtml

increase questionnaire response although those that used these were unsure of their effect. Those that used monetary incentives felt that these should be given as a "*thank you*" rather than as payment. Larger monetary incentives (>£50) were perceived as coercion or bribery by interviewees across all three groups interviewed. Interviewees thought that ethics committees were now more accepting of the use of small monetary incentives and of reimbursing participants' trial related expenses. Offers of entry into prize draws were seldom used, but thought by some interviewees to be a cost effective alternative to incentives and acceptable to ethics committees.

> *"If we said to the Ethics Committee "every participant will receive £50 per visit", um, I …. imagine that the, ….Ethics Committee might think then we're coercing people"*
>
> ***Trial manager interview 7***

> *"Say, well if you come along we'll give you a hundred pounds or whatever it might be. We don't feel that's an ethical thing to do for these types of studies".*
>
> ***Trial manager interview 26***

There was uncertainty among TMs about when to administer an incentive during follow-up. Some gave a small incentive at different times e.g. £5 at randomisation and £5 at trial completion. Others sent the incentive with the questionnaire, or on receipt of a completed questionnaire.

Non-monetary incentives such as pens, certificates of appreciation, key rings and mugs were sometimes used in an attempt to improve both participant recruitment and retention. These were used to market the trial, and in the case of pens, to encourage participants to return completed questionnaires. Interviewees thought gifts could be perceived by participants as patronising, not useful and a waste of public money.

> *"I think people are very sensitive about money especially in the public sector, ….if they see something that could be considered wasteful or extravagant, I don't think it would go down too well. Because…… if you get involved in a study that's being funded from public funds and from charity, you know, it's quite an altruistic thing to do, I don't think people are looking to be rewarded for it ….then they see you wasting money on pens and mugs or, I don't think it would go down very well".*
>
> ***Trial manager interview 7***

For peer review only - http://bmjopen.bmj.com/site/about/guidelines.xhtml

Some interviewees thought that the money used to finance such incentives could be better used to subsidise follow-up visits for trial participants. Indeed there was consensus amongst interviewees about the importance of reimbursing participants' travel and parking expenses associated with such visits. Some interviewees differentiated this from giving incentives to participants. Others recounted cases where participants did not want to be reimbursed particularly if their motivation for taking part was altruistic, and others told of occasions where the offer of money to cover expenses made participants feel valued, but the interviewees thought that these cases could vary by the participants' socio-economic group.

> *"We actually gave them ten pounds for their travel costs, once at the beginning and once at the end.. and a lot of them didn't want it, you know, they'd say, oh... And I'd say, well just pop it in your local charity".*
>
> ***Research nurse interview 2***

Trial managers and PIs thought that the ethics approval process was lengthy and complicated, but interviewees across all groups thought it was important that ethics committees asked questions about the use of incentives. They felt that ethics committees still varied in their opinion about the use of these. Some PIs said that it was helpful to know who was on the ethics committee and that approval to use incentives is generally granted when a clear justification for the use of these is given. Others thought the use of incentives was likely to be approved if the financial incentive involved paying the travel expenses of the participants.

Communication strategies

Communication strategies were spontaneously mentioned by all interviewees as the main method to retain participants in primary care trials. RNs frequently used telephone calls, for example, to arrange or re schedule appointments or to remind participants to return questionnaires. Both RNs and PIs were conscious of not harassing participants with telephone reminders.

> *"Let's say you've made three appointments and they haven't turned up for three appointments, then you would phone them and say I'm really sorry, you know, you're obviously not interested or you're presumably not interested, because you don't want to be harassing them and they have a right to withdraw from that study, so you have to respect it, and their circumstances could have changed.".*
>
> ***Research nurse interview 3***

For peer review only - http://bmjopen.bmj.com/site/about/guidelines.xhtml

Letters were used predominantly by TMs to communicate with trial participants. Electronic coloured signatures, franked institutional logos on outward envelopes, different coloured envelopes, and first class outward post were used to attract the attention of participants. Recorded delivery was used sometimes to send important study-related materials, or for resending questionnaires to non-responders. Some interviewees thought this was useful to find out if participants had moved, but others thought it was inconvenient for the participant if they had to collect trial supplies from a post office. 1st class post was used to send trial materials to participants. While some TMs and RNs thought that this was a waste of financial resources, other TMs thought 1st class stamped letters were more likely to be opened by participants. They were, however, uncertain of the effect of sending post by 1st class on improving questionnaire response.

Letters, when used, were usually signed by TMs rather than PIs; the TMs thought consistency in the signatory was important for retention. Some TMs sent letters to participants in time for these to arrive at the weekend. PIs thought personalised letters and the tone of the language used were important for retention and also that mail-outs were necessary but burdensome for trial staff. Greeting cards were used occasionally; on the whole however, interviewees were negative about their use, with the exception of the use of birthday cards in paediatric trials, and also Christmas cards sent to sites from coordinating centres.

> *"I'm routinely now suggesting that we send out birthday cards to children where we have that information. We've done Christmas cards to participants on behalf of the trial team but there's no evidence that it works, but I think that it's a gesture and it's in the right direction, it's better than nothing".*
>
> ***Trial manager interview 27***

> *"…There were different strategies that we've had for keeping practices involved in the trial, … sending out Christmas cards and things, ….[to] keep the practice remembering that they're involved in a trial and keep your trial in their mind, but that's been at a higher-up… one step up from the participant".*
>
> ***Principal investigator interview 18***

TMs and research RNs used email contact with participants who preferred this approach. SMS text messaging was used less, but thought to be potentially useful for retention. The PIs and RNs thought that appointment reminder text messaging similar to that used for NHS appointments would be useful.

For peer review only - http://bmjopen.bmj.com/site/about/guidelines.xhtml

Newsletters were used by TMs and PIs to keep trial staff at sites and participants informed of trial progress, but some TMs were uncertain about the impact of these on trial retention.

> *"Newsletters are another thing that we all love to do… we recently sent out a newsletter and we were all pleased with ourselves … and then I'll say to them [participants] "oh did you get the newsletter?" And… a good proportion go "ah, um, I think so…". And I think.. when people receive so much stuff through the post it's just another bit of…..junk mail".*
>
> ***Trial manager interview 7***

PIs thought that answers to frequently asked questions on trial websites could increase retention (Box 1).

New questionnaire format strategies

The interviewees did not spontaneously mention using modifications to questionnaires to improve response / retention, although some PIs and TMs used shorter questionnaires for non-responders. When asked specifically about the effect of questionnaire length on retention, interviewees expressed a preference for shorter questionnaires, which were thought to be better for increasing postal questionnaire response. Interviewees also thought that long questionnaires administered frequently led to "*questionnaire fatigue*", and a few thought that dividing long questionnaires into several short questionnaires could be useful to reduce the burden of data collection. However, some PIs thought that if the questionnaire content was of interest to participants, then the length of the questionnaire may not affect response. TMs and PIs mentioned that involving patient representatives in the design and pre-testing of questionnaires could improve retention. Many suggestions were also offered for improving questionnaire design in order to increase questionnaire response (Figure 1). Some TMs had noticed that questionnaires could influence response. For instance, questionnaires sent before clinic visits were thought to enable participants to complete the questionnaire at home before the next clinic visit. Posting questionnaires on Thursday to arrive on Saturday was also thought useful for improving questionnaire response and therefore retention of participants.

How the questionnaire was administered and by whom was thought to also impact on retention rather than questionnaire length. TMs and PIs provide a prepaid envelope for return of postal questionnaires to reduce the cost of postage to participants and the inconvenience of

For peer review only - http://bmjopen.bmj.com/site/about/guidelines.xhtml

having to buy a stamp. To minimise respondent burden, clear instructions on what is to be done with the questionnaire once it is completed was also thought by TMs to contribute to postal questionnaire return.

Use of other strategies to improve retention

The use of behavioural or methodology strategies was not mentioned spontaneously. Behavioural strategies were described to interviewees as *"Arranging workshops to give participants information about goal setting and time management"* for example in parenting and exercise trials. Similarly comparing a blind trial to an open trial was described as an example of a methodology strategy. When asked specifically about the use of these strategies, interviewees were generally negative. Such strategies were discussed in the following ways: "*strange*", "*preachy", "condescending*", "*never came across it", "would never think of doing it*", *I'd be surprised if it worked*" and "*would need evidence that it worked*". Some thought that these should only be used as part of a trial intervention and could affect the generalisability of trial results.

Although the interviewees were familiar with the concept of blind and open trials, they were not aware of the use of either design as a strategy to improve retention. Interviewees felt that using an open trial design to improve retention could bias trial results, because blinding is used to reduce bias and also participants may drop out if they are not allocated to their preferred intervention. They also felt that some interventions, for example therapist-led behavioural interventions, cannot be evaluated using blind designs.

One TM had used case management to improve trial retention. This involved the trial manager arranging services to enable participants to attend follow-up appointments. The interviewees generally thought that case management could improve trial retention in some groups e.g. the elderly, disabled and participants with low incomes but they also thought this could create dependence on the service. The impact of this strategy on retention was thought by some to depend on the personality of the case manager.

Other factors thought to contribute to retention in primary care trials (Box 1)

Use of participant's time

For peer review only - http://bmjopen.bmj.com/site/about/guidelines.xhtml

A key factor mentioned was reducing the burden of follow-up for participants in terms of time, and also the type of data collected (e.g. biomedical specimens, long questionnaires) . The time participants spend getting to, and from, and at follow-up visits; giving participants adequate time at follow-up visits, and ensuring that participants were not kept waiting too long for follow-up appointments were all considered important.

> *"The cost to the patient in time and effort to get to the surgery,…what time these appointments would be. How long they would take, how much effort it would take to get the patient… and also, if it's a research project, I always feel that patients shouldn't wait, …it's up to the nurse to make her appointments work so that she doesn't have anybody waiting there for any length of time,*
>
> ***Research nurse interview no 1***

RNs felt that some participants dropped out because they did not have enough time to participate. To reduce multiple return visits to sites, some TMs synchronised treatment and condition monitoring follow-up with trial follow-up visits to improve retention.

Engaging with participants at recruitment

The recruitment visit was seen as a very important time for engaging participants and giving them important information about trial follow-up procedures.

> *"It [the recruitment visit] sets the stage for the participants relationship with the trial …………. it's important to get that right, right from the start…. that affects whether people think it's worthwhile coming back…"*
>
> ***Trial manager interview 26***

Establishing a rapport with participants that continues throughout follow-up was also thought to lead to retention.

> *"At the end of the day it's how the nurse and GP …….establish a rapport and a relationship with the participant,… that is the key issue in keeping participants in a study".*
>
> ***Research nurse interview 12***

Good rapport was thought to be maintained through continuity of trial staff at the trial site for follow-up visits. Some TMs thought that having "*personal*" or "*good*" communication with

For peer review only

participants contributed to retention. Some interviewees across all groups mentioned the impact of the personalities of trial staff on trial retention. Interviewees thought trial RNs needed to be "*flexible*" and "*sympathetic*" toward participants, while TMs needed to be "*competent*", "*personable*", "*persistent*", "*enthusiastic*", and "*good communicators*".

Perceived benefit of participation to the participant

Most interviewees thought that if trial participants perceived an extra benefit of participation this would contribute to retention.

> *"In studies where (there) is … constant monitoring ....they don't mind giving..... samples again and again as part of the trial..... But I ...think .... if they can see some benefit then they'll do it but if they can't or if they've already got that benefit from giving the sample at the first time point they might not want to do it again and again"*
>
> ***Trial manager interview 10***

Some of the benefits identified were extra health monitoring for example: "*ECG*" "*BP*" "*cholesterol*" and other "*health checks*" as well as having the extra attention from staff. However, these perceived benefits were thought to be trial dependent.

Other factors thought to increase participant retention were participant altruism, where people wanted to give something back to society:

> *"…They [participants] feel as though they want to do good because they often, a lot of them say "oh it's payback time", they think the NHS has been good to them so they want to give something back".*
>
> ***Trial manager interview 7***

and tailoring trial retention strategies to different population groups. For example in mental health trials, some interviewees had found that some participants may wish to be followed at home rather than returning to sites for follow-up visits.

Factors thought to contribute to loss to follow-up in primary care trials (Box 2)

Participant age was thought to affect retention in primary care trials. Several age groups were highlighted as challenging, for example, teenagers opting out of a trial when they reach the age of consent; working mothers juggling school runs with work time and follow-up visits;

For peer review only - http://bmjopen.bmj.com/site/about/guidelines.xhtml

elderly participants either losing their independence or being involved with extended family caring activities, and young men with other interests.

> *"Certainly, the age group does seem to make quite a lot of difference with younger males being very difficult to get in and keep into trials (laughs) in the normal course of events or getting to send back questionnaires. They've got so many other interests, haven't they?"*
>
> ***Research nurse interview 6***

The disease area under investigation was also thought to impact on retention of trial participants. Some interviewees thought that if participants did not perceive a benefit from participation they might not return for their follow-up visit. They thought for example that in obesity trials, if participants were not losing weight they might not return to be weighed perhaps due to the shame and stigma associated with not achieving their expected aims. Retention in trials involving behaviour change was thought to be problematic if change targets were not achieved by participants. For example, interviewees were generally in agreement that retention can be problematic in trials involving participants with a mental health issue.

> *"For smoking cessation or weight management or any of these things where certain behaviour is expected or wanting to be demonstrated. If they're not achieving it they are more likely to just drop out"*
>
> ***Research nurse interview 9***

Interviewees also felt that participants with chronic diseases may have more interest in the treatment of their condition and return for follow-up visits which would contribute to retention. Others thought healthy volunteers may drop out of trials because of lack of interest.

Some interviewees reported that the working environment at general practice trial sites might impact upon trial retention and that it was important to have a staff member at the site to "*champion*" the study locally. Part-time RNs reported working in isolation, and sometimes struggled to find a consulting room for follow-up visits, restricting the availability of flexible appointment times to offer participants.

> *"The last study that I worked on, ..I struggled to find .. a room, the practice were incredibly accommodating, and really wanted me to have a room, but it just is very, very tight. ...it has been, a major issue for me…It was affecting follow up for me because I was not always able to have a room when I was available, that ….would*

For peer review only - http://bmjopen.bmj.com/site/about/guidelines.xhtml

> *have been potentially available for participants, so it just narrowed down the opportunities for me to offer to participants to come in"*
>
> ***Research nurse interview 5***

Reception teams are generally the first point of contact for trial participants at trial sites, some interviewees said that it was off-putting for participants who return for follow-up if practice staff or receptionists are not aware of a trial.

Discussion

Statement of principal findings

Based on in-depth interviews with primary care trial personnel we identified that incentive, communication and questionnaire strategies are used to try to increase retention in primary care RCTs. Making efficient use of participant time and engaging with them were also thought to promote retention as was the perception by participants that they would have some benefit from participation. Interviewees also thought that the age of participants the disease / condition and research environment were all contributing factors to loss to follow-up.

Strengths and weaknesses

There is limited work on the retention of participating individuals in the area of primary care trials. Work to date is grouped with recruitment strategies for participants and trial sites[12,13]. This study is the first to explore the use of retention strategies in UK primary care randomised trials and the factors associated with retention and loss to follow-up. Interviews were conducted with researchers experienced in the design, leadership, management, and implementation at site level of UK primary care trials who had used strategies to improve retention. Prior to this study some of the interviewees reported that they had not thought deeply about loss to follow-up or the strategies to overcome this. With the focus by funders on recruitment rather than retention targets, this study raises awareness of the complexity of retention in primary care trials among primary care researchers. A limitation of this study is that the thoughts and experiences of trial participants who have dropped out or of those who were retained in trials were not captured to further explore the barriers and facilitators to retention in primary care trials. Also it is not clear how generalisable the results are to RCTs in general.

For peer review only - http://bmjopen.bmj.com/site/about/guidelines.xhtml

Meaning and implications

These results enhance the interpretation and implementation of the results of the Cochrane systematic review on ways to improve retention in trials[1], and used together, can inform the future use of effective strategies to improve retention in primary care trials.

There was agreement across the groups that small monetary incentives were acceptable in the context of increasing questionnaire response, and were likely to be viewed favourably by ethics committees, although there was uncertainty among those interviewed about whether monetary incentives should be given up front or offered on receipt of data. The systematic review shows that both increase postal questionnaire response[1].

Non-monetary incentives are also used in primary care trials, but with considerable scepticism as to their value to the extent that it was thought that these could have a negative impact on trial retention and questionnaire response. As this type of incentive was not found to be effective in the systematic review, primary care trialists or indeed any trialists may wish to reconsider the use of this type of incentive in randomised trials.

Communication strategies appeared to be commonly used to increase return to sites for follow-up and to improve return of questionnaires. 1st class post is used routinely for outgoing post. Based on two studies, Edwards review found that 1st class post was effective at increasing questionnaire response but was not specific to RCTs. However the systematic review of strategies in RCTs demonstrates no benefit of using 1st class post for outgoing mail[14]. Therefore, 2nd class post can probably be used for postal correspondence with primary care trial participants, with little impact in loss to follow-up. Also, some trial managers did post letters so that they would arrive on a Saturday to trial participants. However, early versus later administration of letters was also not effective in our review.

Although recorded delivery was used for important study related materials and to resend questionnaires, this was thought to be potentially frustrating for participants if delivery was made while they were out. Nevertheless recorded delivery of questionnaires compared with a telephone reminder was found to be effective for improving questionnaire response[1,5]. To avoid any inconvenience associated with the use of this strategy, preplanning the delivery time with the participants and reminding them to expect a recorded delivery of trial materials is recommended if this strategy is to be used in future trials.

For peer review only

The trial managers describe enhancing the letters they send. However, the systematic review showed no effect for using such enhanced letters[1]. Although the TDM, a communication package that encompasses sending hand prepared letters with a series of reminders, to improve questionnaire response, was found to be effective, based on the results if one trial reported in 1996[15]. This approach may not be applicable to all participant groups (e.g. young people), or for trials using electronic data collection methods.

Different types of reminders are routinely used by primary care researchers to improve trial follow-up, but the systematic review found no clear evidence that reminders and prompts in addition to the standard trial follow-up procedures are beneficial to retention. Also, Edwards found that telephone reminders did not improve questionnaire response[14]. Reminders are part of the standard trial follow-up procedures in trials for collection of data from non-responders. They can play an important part in participant engagement especially if there is little face to face contact with trial participants. However, the interviewees in this study thought too many additional reminders could be perceived as harassment by trial participants. This raises questions about the use of additional reminders to participants.

Several alternative questionnaire formats were thought to be potentially useful for increasing response, but not used as first-line strategies. Shorter questionnaires were used by some interviewees to increase questionnaire response. Although Edwards found that shorter questionnaires increased questionnaire response in research generally[14], this was not borne out by the systematic review in randomised trials[1]. A strategy also mentioned but not yet evaluated is to divide long questionnaires into several short questionnaires to reduce the burden for participants of data collection associated with longer questionnaires. This may not be practical in trials where there are a number of follow-up questionnaires to be completed.

Behavioural and methodological strategies were not used in the context of these UK primary care trials. The interviewees were particularly negative toward the use of behavioural strategies and there was no effect found for these in the systematic review[1]. The interviewees also thought that blinding participants to their allocated intervention would improve retention. However, our systematic review found that an open trial design was more effective than a blind design in the context of secondary osteopathic fracture prevention[16]. Avenell argues that double blind trials do not reflect usual health care procedures, and that open trials, where the

For peer review only - http://bmjopen.bmj.com/site/about/guidelines.xhtml

intervention is compared to no treatment or usual treatment, could give a better measure of the differential effects in normal care settings[16]. The open trial design, if used as a stand-alone strategy to improve retention, would need to be considered in the context of the overall aims and objectives of the trial. Further evaluation of this strategy is needed in different trial contexts and settings if it is to be adopted as a strategy to improve trial retention. Although case management was seldom used, elements of this strategy were thought to be useful but there is no evidence that it is effective[1].

Integrating qualitative research with systematic reviews

Integrating the results of this qualitative study and the Cochrane systematic review helped us understand the different experiences primary care researchers have had using the retention strategies identified by the review. The qualitative results support some of the results of the review and demonstrate the potential transferability of the different effective retention strategies to trials conducted in primary care. The results also helped identify the factors and barriers that could affect the use of effective strategies in the context of primary care trials and furthermore, helped identify other strategies to be evaluated. This mixed methods approach could be replicated in other research contexts to determine how appropriate and useful the strategies identified by the systematic review are in practice.

While the absolute effects of effective strategies identified by the Cochrane review are modest, this qualitative study highlights the complementary strategies used to achieve maximum retention in primary care trials. Also it suggests that primary care researchers should consider other factors that may affect retention, such as building a rapport with participants and minimising the impact of trial follow-up on their daily lives. Consideration should also be given to the characteristics of the intervention, participants and trial personnel when effective strategies identified by the review are being considered to improve trial retention.

Future research

It was clear from the interviews that there is a move toward contacting participants by email and a desire to use text reminders when automated facilities for these become available for use in randomised trials. These new strategies would need evaluation in future trials.

For peer review only

Further research is also needed to identify potential barriers and facilitators to follow-up in trials e.g. with trial participants from different social, economic, age and disease groups. The results would be useful for primary care researchers and would help target and tailor strategies to meet the needs of different population groups to keep them specifically engaged in trial follow-up[17].

Conclusion

Qualitative research can help to interpret the results of systematic reviews by telling us what researchers do or think happens in research practice. These findings provide a deeper understanding of the complexity of retention in primary care trials and the factors that may facilitate or limit the implementation of effective strategies. The use of small monetary incentives to improve questionnaire response is effective and acceptable. A reconsideration of the use of non-monetary incentives, and certain communication strategies including 1st class post is needed. A similar exploration of barriers and facilitators to retention in other research contexts would help identify ways to improve retention in trials conducted in other research contexts.

For peer review only

For peer review only - http://bmjopen.bmj.com/site/about/guidelines.xhtml

BMJ Open qualitative paper submitted 15.08.2013

Article Summary

Article focus

Strategies to improve retention are widely used in trials but without evidence of effect. Strategies thought to improve retention in UK primary care randomized trials have not been well documented or evaluated. This study explores the use of strategies to improve retention identified by a Cochrane systematic review in UK primary care randomised trials (RCTs).

Key messages

Small monetary incentives, non-monetary incentives and communication strategies are used in primary care randomised trials to improve retention. Behavioural, methodology and case management strategies were not used. Small monetary incentives and communication strategies were thought to be useful for retention and case management thought to be potentially useful. Interviewees were negative about the use of behavioural, methodology and non-monetary incentive strategies.

Factors thought to contribute to retention are: effective communication, participant altruism and perceived benefits of participation for participants, burden reduction, personality and flexibility of trial staff.

Factors thought to contribute to loss to follow-up are: participant age, disease/condition and research environment.

Strengths and limitations

This study is the first to explore the use of retention strategies in UK primary care randomised trials and the factors associated with retention and loss to follow-up. Interviews were conducted with researchers experienced in UK primary care trials who had used strategies to improve retention.

A limitation is that the thoughts and experiences of trial participants have not been gathered to further explore the barriers and facilitators to retention in primary care trials.

For peer review only

For peer review only - http://bmjopen.bmj.com/site/about/guidelines.xhtml

BMJ Open qualitative paper submitted 15.08.2013

Acknowledgements

We would like to thank all twenty nine interviewees who gave of their time and thoughts on the complexities of trial retention in primary care.

Statement of funding and competing interests

All authors have completed the ICMJE uniform disclosure form and declare no competing interests. This project was funded by the Medical Research Council Population Health Sciences Research Network grant number PHSRN 30

For peer review only

Reference List

(1) Brueton VC, Tierney J, Stenning S, Nazareth I, Meredith S, Harding S et al. Strategies to improve retention in randomised trials . *Cochrane Methodology Group* 2013; in press.

(2) Janson SL, Alioto ME, Boushey HA. Attrition and Retention of Ethnically Diverse Subjects in a Multicenter Randomized Controlled Research Trial. *Control Clin Trials* 2001; 22(6, Supplement 1):S236-S243.

(3) Williamson I, Benge S, Barton S, Petrou S, Letley L, Fasey N et al. Topical intranasal corticosteroids in 4-11 year old children with persistent bilateral otitis media with effusion in primary care: double blind randomised placebo controlled trial. *BMJ* 2009; 339(dec16_1):b4984.

(4) Dangour AD, Allen E, Elbourne D, Fasey N, Fletcher AE, Hardy P et al. Effect of 2-y n-3 long-chain polyunsaturated fatty acid supplementation on cognitive function in older people: a randomized, double-blind, controlled trial. *Am J Clin Nutr* 2010; 91(6):1725-1732.

(5) Tai SS, Nazareth I, Haines A, Jowett C. A randomized trial of the impact of telephone and recorded delivery reminders on the response rate to research questionnaires. *J Public Health* 1997; 19(2):219-221.

(6) McColl EM, Eccles MPM, Rousseau NSB, Steen INP, Parkin DWD, Grimshaw JMP. From the Generic to the Condition-specific?: Instrument Order Effects in Quality of Life Assessment. [Article]. *Med Care* 2003; 41(7):777-790.

(7) Cockayne S, Torgerson D. A randomised controlled trial to assess the effectiveness of offering study results as an incentive to increase response rates to postal questionnaires [ISRCTN26118436]. *BMC Medical Research Methodology* 2005; 5(1):34.

(8) Man MS, Tilbrook HE, Jayakody S, Hewitt CE, Cox H, Cross B et al. Electronic reminders did not improve postal questionnaire response rates or response times: a randomized controlled trial. *J Clin Epidemiol* 2011; 64(9):1001-1004.

(9) Trial Managers Network. 2013.

Ref Type: Online Source

(10) Pope C, Ziebland S, May N. Analysing qualitative data. In: Catherine Pope, Nicolas May, editors. Qualitative Research in Health Care. second ed. London: BMJ Publishing Group; 2000. 75-88.

(11) Farmer T, Robinson K, Elliott SJ, Eyles J. Developing and Implementing a Triangulation Protocol for Qualitative Health Research. *Qual Health Res* 2006; 16(3):377-394.

For peer review only

(12) Leathem C, Cupples M, Byrne M, O'Malley M, Houlihan A, Murphy A et al. Identifying strategies to maximise recruitment and retention of practices and patients in a multicentre randomised controlled trial of an intervention to optimise secondary prevention for coronary heart disease in primary care. *BMC Medical Research Methodology* 2009; 9(1):40.

(13) Graffy J, Grant J, Boase S, Ward E, Wallace P, Miller J et al. UK research staff perspectives on improving recruitment and retention to primary care research; nominal group exercise. *Fam Pract* 2009; 26(1):48-55.

(14) Edwards PJ, Roberts IG, Clarke MJ, DiGuiseppi C, Wentz R, Kwan I et al. Methods to increase response rates to postal and electronic questionnaires. *Cochrane Database of Systematic Reviews 2009, Issue 3 Art No : MR000008* 2009;(3).

(15) Sutherland HJ, Beaton M, Mazer R, Kriukov V, Boyd NF. A randomized trial of the total design method for the postal follow-up of women in a cancer prevention trial. *Eur J Cancer Prev* 1996; 5(3):165-168.

(16) Avenell A, Grant AM, McGee M, McPherson G, Campbell MK, McGee MA et al. The effects of an open design on trial participant recruitment, compliance and retention - a randomized controlled trial comparison with a blinded, placebo-controlled design. *Clinical Trials* 2004; 1(6):490-498.

(17) Kimmel SE, Troxel AB. Novel incentive-based approaches to adherence. *Clin Trials* 2012; 9(6):689-695.

For peer review only

For peer review only - http://bmjopen.bmj.com/site/about/guidelines.xhtml

Table 1 Characteristics of interviewees

Characteristics	Number of interviewees
Male	10
Female	19
**Role**	
Research nurse	9
Principal investigator	10
Trial manager	10
**Location**	
London	8
Midlands	8
Northeast England	3
Southwest England	3
East of England	5
Scotland	2
**Unit**	
University/Research organisation based	23
GP practice site based	6

Table 2 Other sample characteristics

Other characteristics	Number of interviewees
No of interviewees from trials published between 2000-2004	6
No of interviewees from trials published between 2005-2010	23
No of interviewees from trials conducted through the MRC GPRF	19
No of interviewees from trials conducted through other research units	10
No of interviewees recruited from trials with loss-to–follow-up levels*:	
>20%	10
<20%	17

* Two nurses not linked to a sample frame trial

For peer review only

Table 3 Use of retention strategies identified by the Cochrane review by primary care research team members

Strategy	Principal investigators	Trial managers	Research nurses
**Incentives**			
Monetary incentives	One PI had never used incentives	Used to increase response to postal questionnaires. Some split the incentive and gave participants a small incentive at different times e.g. £5 at randomisation and £5 at completion. Some send the incentive with the questionnaire; others on receipt of the questionnaire.	Mostly in pharmaceutical trials. Sometimes in academic trials.
Vouchers	Used in trials they lead	For those that had used vouchers, types used included: high street stores (M&S, ASDA, Tesco) online gift tokens (Amazon, Waterstones and WHS), or fresh fruit vouchers. Some had tailored the type of voucher to participant requests e.g. one interviewee used B&Q home improvement vouchers. These were sent by TMs with questionnaires or with a promissory element that on completion of questionnaires the participant would receive a voucher. Mobile phone top up vouchers used in trial to retain young people.	Administered at site level by research nurse. Used as a thank you from the trial team.
Gifts	One PI used gifts. Given for practical reasons i.e. carrying study materials	Used gifts. Seldom mentioned spontaneously. Used as reminders to keep trial at the forefront of participants and clinicians' minds or as a thank you for participants. Uncertainty about the effectiveness of gifts given on behalf of the team to either participants or clinicians.	RNs used gifts to retain people in randomised trials if prescribed. Seldom mentioned spontaneously. Used to increase recruitment rather than retention.
**Communication**			
Letter	Used in trials they lead	Uncertain of effectiveness. Trial managers signed letters sent from coordinating centre. Electronic and hand signatures used. Name of CI mentioned. Sometimes used letters as a stand-alone communication method.	Only used if specified in trial protocol/ study procedure manual.
Post	Used in trials they lead	2nd class used for pre-paid reply envelopes to reduce cost. 1st class used to send letters. Recorded delivery for second copy of questionnaire. Would confirm patient had moved. Hand delivered letters if participant difficult to contact.	Only used if specified in trial protocol/ study procedure manual. Sometimes hand delivered.
Telephone contact	Used in trials they lead	Used sometimes	Preferred method. Used for follow-up contact.
SMS texting	Used in trials they lead	Used when all other methods fail or if prescribed for follow-up	Used if prescribed. Seldom used.

For peer review only

Strategy	Principal investigators	Trial managers	Research nurses
Emails	Used in trials they lead	Used when email address given	Used when email address given.
New questionnaire formats	Some used shorter follow-up questionnaire with the second reminder.	Used a briefer follow-up questionnaire with a second reminder.	Short ones used for telephone interviews for non responders. Different lengths used for face to face interviews at site.
Behavioural	Not used	Not familiar with this	Not used
Case management	Not used	Used by one	Not used but aware of this
Methodology	Not used	Not used	Not used

For peer review only

Table 4 Speculation about the use of retention strategies identified by the Cochrane review in primary care randomised trials

Strategy	Principal investigators	Trial managers	Research nurses
Monetary incentives	Participation in pharmaceutical company trials paid. Payment could be perceived as bribery used to coerce participants, if the amount given is excessive e.g. >£50 acceptable to increase questionnaire response. Young people motivated by money. Older people participate for altruistic reasons. Some felt "cash in the hand" was a useful motivator. One suggested increasing the value of incentives for each questionnaire returned. Incentive values used £5 - £20 cash or vouchers. Considered reasonable for adults and children.	Could be perceived as bribery used to coerce participants, if the amount excessive >£50. Incentives are acceptable to increase questionnaire response. Uncertainty about when to administer incentives during follow-up. £5 - £20 considered reasonable for adults and children.	Could be perceived as bribery used to coerce participants, if amount is excessive i.e. >£50. Small incentives acceptable to increase questionnaire response. Participants should not be out of pocket. £5 - £20 considered reasonable for adults and children.
Vouchers	Some never use vouchers as incentives. Unclear if these were successful. Some thought vouchers might not be appropriate for some social groups. Generic vouchers most appropriate.	One TM never used vouchers. Unclear if these were successful. Vouchers thought to be less open to corruption, but a burden. Some TMs thought some vouchers might not be appropriate for some social groups. Generic vouchers are more appropriate.	Vouchers good for retention. Generic vouchers more have more universal application. Do not have to deal with cash at sites.
Gifts	Gifts not given on behalf of the study team by PIs. Interested to know what participants thought about gifts. Some thought these were patronising, a waste of money and associated with charity fundraising and participants might question the use of public money for this purpose. Some thought participants would prefer monetary alternatives. Participants would not participate in a trial for the gifts alone	For altruistic participants gifts could be perceived as "insulting" or "uninteresting". Some thought participants might be suspicious about the use of public money on gifts. This might be interpreted as wasteful or extravagant use of public money especially if motivated by altruism to participate. Could be associated with Charities and fund raising.	Gifts, offered or given, should be useful, appropriate, not patronising and tailored toward the disease/condition and participant group. One thought pens were "incidentals" not gifts. Felt gifts given would not retain participants in trials.
Communication			

For peer review only

Strategy	Principal investigators	Trial managers	Research nurses
Letter	Few comments on usefulness. Important factors tone and personalisation. Participant input important. Don't want to be seen as interfering in trial management. Mail outs boring and burdensome for trial managers.	Consistency of signatory more important than who signs. Name of CI mentioned. Some participants respond to authority. Franking with institutional logo used. Effect unknown. "Hate" brown envelopes, perceived as "bills" "junk mail" "letter from tax man". One thought no evidence that envelope colour mattered. Uncertain about hand written envelopes over preaddressed.	May not be opened by participants. Could get lost/ opened by someone else. Makes it look different with Logo. Might work.
Post	Not raised spontaneously by PIs	1st class used to send letters. More likely to be opened.	1st class post waste of money
Telephone	Involved in decisions about use of telephone contact. Calls should be conversational to build rapport with participant. Would be "daunting" if they heard from a PI. Young people change telephone numbers. Difficult to get hold of. Be careful not to harass participants.	Nurses use this, should use it more to keep in touch with participants, might increase retention. Reminder call before due to attend would increase retention. Calls may not be answered fear of telemarketing.	Prefer telephone to letters. Engage in dialogue. One thought telephone contact more intrusive letters. Be careful not to harass participants.
SMS texting	Good for young people. A system based on the automated NHS clinic appointments system would be useful. Need evidence it works.	Used for young people. Young people change numbers.	Good for young people. Utilising similar system to automated NHS clinic appointments system might be useful. Limited to people that can text
Email	Every mode should be considered	Used for people with busy lives	Good for people with busy lives
**New questionnaire formats**	One thought getting the balance right between length, readability, content and the acceptability of the topic were important. If this could be done without compromising what the questionnaire intends to measure. If questionnaire administered at clinic by nurse rather than post response could increase.	Long questionnaires considered off putting, no matter how engaged they were in a trial. Adding extra questions was another barrier to completion and non return. Having several short questionnaires could encourage return. Not so short that participants would not see the point of completion. Questionnaires administered at the clinic by a nurse rather than by post could increase response. The time at which questionnaires are posted was felt to be a factor associated with improved response. Questionnaires sent before a clinic visit enables the participant to complete a	Shorter questionnaires of 30-50 pages were thought to be long and off putting for participants to complete, no matter how engaged they were in a trial. The addition of extra questions that were not often analysed was another barrier to completion and non return. Having several short questionnaires was thought to encourage return, these should not be so short that trial participants would not see the point of completing them. People get used to questionnaires.

For peer review only

Strategy	Principal investigators	Trial managers	Research nurses
		questionnaire at home before their clinic visit.	
**Behavioural**	Don't understand it. May contaminate intervention. Generalisability of results questionable. "*Preachy*". Need evidence. Sounds like giving them a bit of the intervention. Could lessen effects of the treatment intervention.	Work shops about motivation condescending to participants. Don't know if it would work. Time consuming and expensive. May work with some groups. Don't like it and don't know much about it. Could not imagine using it. May be a burden for coordinators and participants.	Not heard of this. Workshop potential problems: non attendance, burden for participants and organisers, time commitment.
**Case management**	Where the intervention has to be reproduced could be problematic. Useful if there is not much benefit for participants. Takes time to arrange. Expensive. Annoying if participant does not turn up. Would not have thought of doing it. We do some of this.	Important for elderly. Costly. Should be done, rarely have luxury to do it. Should not be a substitute for good communication. Degrees of case management used. Logistics need working out.	Could be useful. Some heard about this. "*Cannot fail*", expensive, logistics need to be worked out. Useful for elderly. Some do elements of this. Personality of case manager important.
**Methodology**	Could not blind some participants to e.g. therapist lead interventions. Depends on study. Could bias results, blinding should be done where possible. Not suitable as a strategy to reduce attrition.	Do not know enough and never thought about it. Risks: would drop out if open and did not get allocation to preferred treatment.	Don't know enough about this. Participants could drop out if not in preferred allocation group.

For peer review only

Box 1 Factors thought to contribute to retention in primary care trials

Reducing the burden of participation for participants

"The burden of activity on the patients, how easy it is for them, how long it's going to take, how intrusive in terms of blood samples or answering huge long questionnaires, it's partly the complexity as well, if it's very simple for them to do, often you just want very simple data back from people, alive or dead, or specific complications, if it can be done either with a, increasingly with a click of a button by sending a text or an email to people or just a very you know, reply paid cards so you're just getting the data back from them, I suppose the other thing is….trying to do electronic follow-up as much as possible so you don't actually have to bother the patient, you have their consent to access their electronic records and either their Primary care data can be interrogated …a way of linking all of those together, like with Cancer Registry, Registrar General data, so I suppose the more you can automate it the easier it becomes".

Principal investigator interview 4

Establishing a rapport with participants

"I think with a lot of studies the rapport with whoever, the sort of teams that they're engaging with, you know, be that the GP and the practice nurse or be it a consultant and a research nurse, if they show interest and passion and enthusiasm for the study that's really, really helpful and then if they're sort of treated well when they come for the follow-up visits……"

Principal investigator interview 28

Effective communication with participants

"I think it is communication, communication at all levels; so the nurse who interfaces with the trial management team behind the coordinating centre, …but her or him listening to the participant's views as well and … feeding them back, so ……. a two-way process. Because we're this side doing the research, we don't know best, we have to listen to… which is, I guess, why we involve consumers in our work as well, so yes, just communication and understanding your population that you're working with".

Trial manager interview 13

"We …..mak(e) sure that we have [communication] strategies in place to get as many …..responses to our postal questionnaires …we send out the questionnaire…, if we don't hear back ….we will ring up to say …."Could you possibly send it back to us?" If we still don't hear …we'll send .. another copy … if we don't have a response from that copy then we'll ring them again…if we still don't hear we've telephoned …again to collect a set of core outcomes to get a response………."

Trial manager interview 14

Personality of trial research staff

"[Being] friendly is very important. And I just think having the right approach to things in general. So

For peer review only

you have a 'can do' attitude rather than a inflexible attitude. You have to have somebody who... Or people who... people who are prepared to go out of their way to do things. And have a very person focused way of doing things."

Trial manager interview 26

"If you're enthusiastic, and can give the information to the participants with,…interest and,… also sympathetic to their, …. queries, always being accommodating, always getting back to them if they have any queries if, there are messages left, being absolutely spot on with, so that they don't feel they've been let down or you haven't been bothered to get back to them."

Research nurse interview 5

"I think there's a paradox here because they [trial managers] need to be obsessional about detail but they also need to be relaxed and flexible in their response to different situations ….that come up, different problems that come up. That's asking quite a lot, I think. So you know it's great if you find somebody who's good at it…..Or be very kind hearted. And prepared to work out of hours!"

Principal investigator interview 25

Flexibility of trial research staff

"They're [participants] do[you] a favour if they are taking part in a clinical trial so we need to try to be flexible, come very early morning …or stay later or they can come after work. Lunch time if it is, you know near to their work but of course like sometimes people work in the city and is away from where they live or it's a long journey. ….If a patient sees that they're going sit there for an hour and a half he says "I can't, I can't afford the time", ….. you get that, you know, quite often…"

Research nurse interview 12

Altruism of participants

"I've always been of the opinion that (…..)healthy volunteers, … they have what can be considered a less of a kind of vested interest in the study (…) they're doing it all out of the goodness of their hearts, there's not a lot in it for them"

Trial manager interview 26

Tailoring strategies to different follow-up groups

"Depending on the trial and the length of the trial and your client group and all those sort of things…you would have a completely different strategy for, you know, …..thinking of Chlamydia, the fifteen to twenty-four year olds, [compared] to something like [a trial involving the elderly] where you're looking at the seventy-five plus….. you do sort of tailor it [the strategy] to your group."

Research Nurse interview 2

\

For peer review only

Box 2 Factors thought to contribute to loss to follow-up in primary care trials

Participant age

Younger participants

"A young girl whose parents had agreed to be in the trial and then, you know, she didn't want to and then decided herself she was going to withdraw. And, you know, I can see the potential for that sort of thing if it's a trial in the sort of pre-teen, teen group that,... they might well change their mind….more interesting things going on in their lives, they've got more after school activities or they've got exams or they've got whatever. And coupled with being more independent and wanting to assert their own ideas".

Principal investigator interview 28

Older participants

"In trials with older people, people will die, people will get frailer and maybe be institutionalised and not be able to continue or not want to continue for those sort of reasons".

Principal investigator interview 28

"There are all the usual things, we assume that older people don't move, there's a astonishingly high level of mobility in the older population. So lost to follow-up can occur just because people say "right, I'm going to go and live in…………." or "I'm going to go and live near my daughter" or "I've off to ……….for the winter". [They] do… a lot of child care, grandparental child care and sorting out their children's divorces and so on and so they get distracted and drop out completely through distraction".

Principal investigator interview 21

Disease/condition health status

"Bi-polar groups, a lot of them find it very, very difficult going to an NHS facility".

Trial manager interview 17

"it depends on what area of medicine it is, for instance, if you're dealing with people with psychiatric illness, there's always a problem with attendance, whether that's within the surgery or whether that's within research".

Research nurse interview 1

"In nutrition studies we're very much bound by looking at healthy individuals to begin with. So from that perspective they may certainly be less motivated than an individual participating in a drug trial who has a severe or debilitating condition that, you know, they want to try and do something about".

Trial manager interview 15

Research environment at the general practice

"if somebody genuinely did want to make the trial work then it was possible to make it work. ……………….it needed somebody to actually manage the trial within that practice. The practices in which we had success I felt that we had a champion there who was fighting our corner for us"

Trial manager interview 27

For peer review only

"the other thing is practice awareness, you know, because you said it's a big practice and if somebody comes in and hasn't got the sort of clock-in bit straight and they go to the desk and say, I've come to see X about this trial and, you know, new receptionist knows nothing. That can be quite off-putting so as long as everybody's engaged …and everybody knows about it".

Research nurse interview 2

For peer review only

Figure 1 Questionnaire design features reported by interviewees to improve questionnaire return

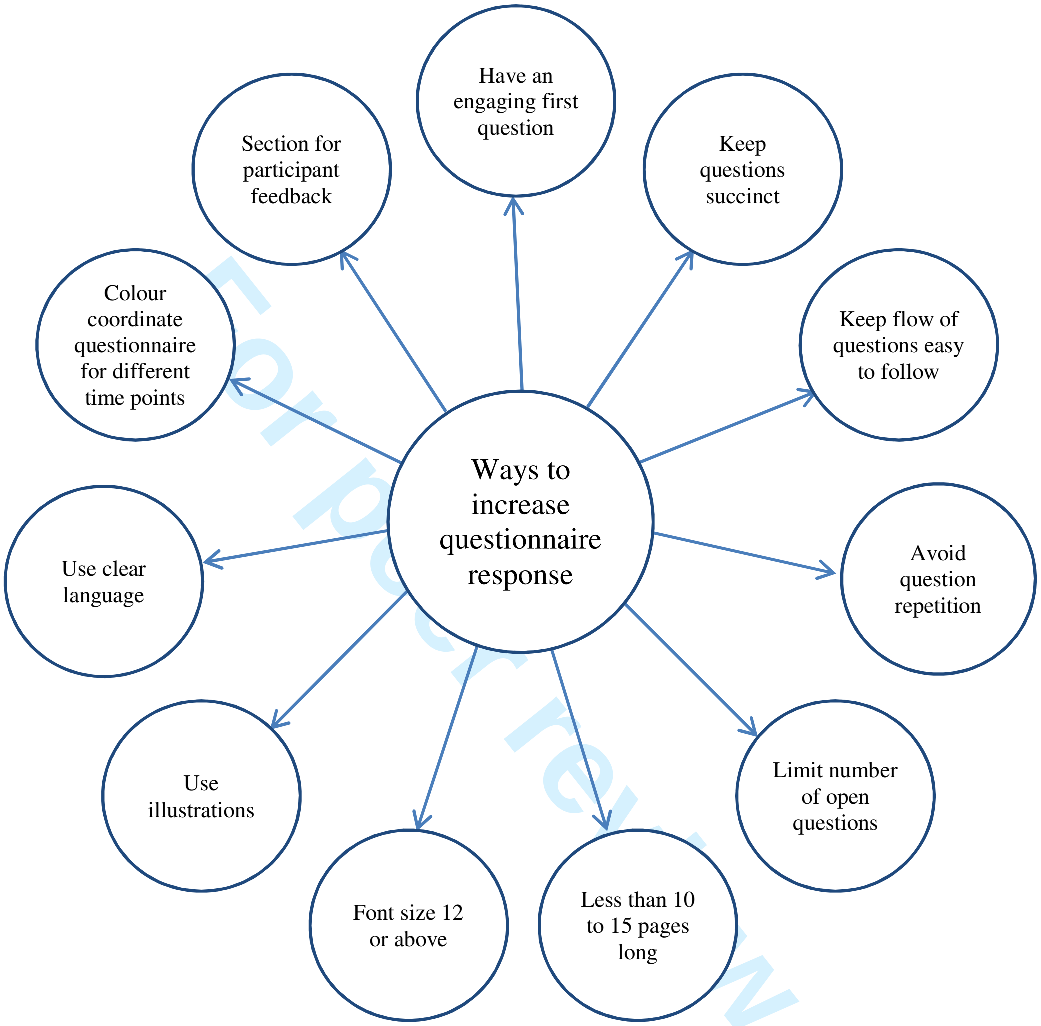

For peer review only

Web Appendix 1: Interview schedule

Background / introduction to the interview

I am interested in attrition from randomised trials. We define attrition as incomplete ascertainment of the primary outcome, but will subsequently use the term "loss to follow-up". I want to explore trialist's opinions on if and when loss to follow-up has been a problem in their experience and the strategies they may have used to deal with or to prevent loss to follow-up. I also want to find out more about trialists preferred strategies for preventing loss to follow-up.

Topic guide questions

1. **Can you tell me about recent trials you have worked on?**
   Were there any with high follow-up?
   Were there any with low follow-up?
2. **What do you think are the factors that lead to loss to follow-up in trials?**
   Why do you think it can be difficult to keep participants in randomised trials?
3. **What are the factors that lead to retention in randomised trials?**
4. **What strategies to increase follow-up have been successful for you in trials you have worked on?**
   Why do you think these have worked?
   What strategies have been unsuccessful?
   Why have these not worked?

Decision making around strategies used to reduce loss to follow-up

1. When do you perceive loss to follow-up to be a problem?
2. How do you decide which strategies to improve follow-up work best?
3. How did/ do you implement the strategies to improve follow-up that worked best?
4. How is loss to follow-up monitored in trials you have worked on?
5. Who deals with loss to follow-up when it presents?

Impact of research governance

1. What do you feel about using incentives to keep participants in trials?
2. Have you had any experience with ethics committees?
3. What do you feel about ethics committees asking about payments or giving other incentives to participants?
4. Has ethics committees approach to payments affected any trials you have worked on in anyway?

Ask this next question at the end of the interview

These are the strategies to reduce attrition/ improve retention identified by the Cochrane review.

For peer review only

Show participant each card separately (See below). Then ask the following questions for each strategy discussed:

1. Have you used these?
2. What do you think about using this strategy?
3. Might you have considered using this strategy in your trial?
4. What could be the advantages of using the strategy?
5. What could be the disadvantages of using this strategy?

Each card shown individually

**Card no 1**
Communication strategies. e.g. email, telephone, text messages, letters signed by different study personnel, type of delivery- e.g. post 1st 2nd class, or recorded delivery, type of envelope used for response.

**Card no 2**
Incentives to either participants or trialists e.g. gifts pens, pins, monetary incentives, offers of incentives, vouchers.

**Card no 3**
Methodological strategies blind versus un blind trials.

**Card no 4**
Different length of questionnaire: Short versus long.

**Card no 5**
Using case management. Having trial assistants manage participant follow-up, for example arranging transport and services to enable participants to keep trial follow-up appointments.

**Card no 6**
Motivational/educational strategies. Such as arranging workshops to give participants information about goal setting and time management.

For peer review only

Use of strategies to improve retention in primary care randomised trials: a qualitative study with in-depth interviews

Journal:	*BMJ Open*
Manuscript ID:	bmjopen-2013-003835.R1
Article Type:	Research
Date Submitted by the Author:	04-Nov-2013
Complete List of Authors:	Brueton, Valerie; MRC Clinical Trials Unit, Meta-analysis Group<br>Stevenson, Fiona; University College London, Research Department of Primary Care and Population Health<br>Vale, Claire; MRC Clinical Trials Unit, Meta-analysis Group<br>Stenning, Sally; MRC Clinical Trials Unit, Cancer and non infections Group<br>Tierney, Jayne; MRC Clinical Trials Unit, Meta-analysis Group<br>Harding, Seeromanie; MRC Social and Public Health Sciences Unit,<br>Nazareth, Irwin; UCL, Primary Care and Population Health<br>Meredith, Sarah; MRC Clinical Trials Unit,<br>Rait, Greta; University College London, Primary Care and Population Health
**Primary Subject Heading**:	Research methods
Secondary Subject Heading:	General practice / Family practice, Qualitative research
Keywords:	Retention strategies, Randomised trials, PRIMARY CARE

SCHOLARONE™
Manuscripts

Use of strategies to improve retention in primary care randomised trials: a qualitative study with in-depth interviews

Brueton V

Research Scientist, Meta-analysis Group
MRC Clinical Trials Unit
Aviation House
125 Kingsway
London WC2B 6NH
e-mail: vcb@ctu.mrc.ac.uk

Stevenson F

Senior Lecturer
UCL Research Department of Primary care and Population Health
Royal Free and University College Medical School
Rowland Hill Street
London
NW3 2PF

Vale CL

Senior Scientist Meta-analysis Group
MRC Clinical Trials Unit
Aviation House
125 Kingsway
London WC2B 6NH

Stenning SP

Senior Statistician
MRC Clinical Trials Unit
Aviation House
125 Kingsway
London WC2B 6NH

Tierney JF

Senior Scientist Meta-analysis Group
MRC Clinical Trials Unit
Aviation House
125 Kingsway
London WC2B 6NH

Harding S

Program leader: Ethnicity and Health

For peer review only

BMJ Open qualitative paper submitted Version 3 VB 4.11.13

MRC Social & Public Health Sciences Unit
University of Glasgow
4 Lilybank Gardens
Glasgow G12 8RZ

Nazareth I

Professor of Primary Care
PRIMENT Clinical Trials Unit
Research Department of Primary care and Population Health
UCL Medical School
Rowland Hill Street
London
NW3 2PF

Meredith S

Deputy Director
MRC Clinical Trials Unit
Aviation House
125 Kingsway
London WC2B 6NH

Rait G

Clinical Senior Lecturer
PRIMENT Clinical Trials Unit
Research Department of Primary care and Population Health
UCL Medical School
Rowland Hill Street
London
NW3 2PF

For peer review only

Abstract

Objective

To explore strategies used to improve retention in primary care randomised trials.

Design

Qualitative in-depth interviews and thematic analysis.

Participants

Twenty nine UK primary care chief and principal investigators, trial managers and research nurses.

Methods

In-depth face to face interviews.

Results

Primary care researchers use incentive and communication strategies to improve retention in trials, but were unsure of their effect. Small monetary incentives were used to increase response to postal questionnaires. Non-monetary incentives were used although there was scepticism about the impact of these on retention. Nurses routinely used telephone communication to encourage participants to return for trial follow-up. Trial managers routinely used 1st class post to try to increase questionnaire response. Shorter questionnaires wereused, and suggestions offered for improving questionnaire design. Interviewees thought that an open trial design could lead to biased results and were negative about using behavioural strategies to improve retention.

There was consensus among the interviewees that effective communication and rapport with participants, participant altruism, respect for participant's time, flexibility of trial personnel and appointment schedules, and trial information improve retention. Participant age, e.g. teenagers, disease type and the work environment at trial sites were thought to contribute to loss to follow-up.

Conclusions

For peer review only

Integration of the findings of this qualitative study with the results of the Cochrane review allowed us to reflect on the gap between the evidence from the Cochrane review and how trialists behave in practice. Trialists describe acting without evidence from the literature and from experience of what works. The use of small monetary incentives to improve questionnaire response is acceptable. A reconsideration of the use of non-monetary incentives, and some communication strategies is needed. A similar exploration of barriers and facilitators to retention in other research contexts would help identify ways to improve retention in trials conducted in other research contexts.

For peer review only

Introduction

Retention in primary care randomised controlled trials conducted across different disease areas and communities can be challenging. Inadequate retention can reduce the power of a trial and introduce bias, particularly if drop-out differs across trial arms. Reasons for loss to follow-up can include a change in the participants' location, withdrawal from treatment and / or loss of commitment to the trial, for example due to complicated treatment regimens[1]. A Cochrane review of strategies to improve retention in trials demonstrated that adding a monetary incentive and offering higher valued monetary incentives increased postal and electronic questionnaire response[2]. Questionnaire response was also increased by recorded delivery of questionnaires, a "package" of postal communication strategies known as the Total Design Method (TDM)[3]and an open trial design[4], although these were based on the results of single trials. The evidence of an effect for shorter questionnaires and questionnaires relevant to the disease / condition was less clear. Also, there was no good evidence that the following strategies improved retention: adding or offering a non-monetary incentive, communication strategies (including use of first class mail), behavioural motivational strategies, new questionnaire formats and case management.

Only six of the 38 retention trials included in the Cochrane review were embedded in UK primary care trials and all targeted improving response to postal questionnaires in order to improve retention[5-8]. However, primary care trials also require participants to return to sites for clinical outcome measurement to assess disease progression[9;10]. There could be important factors that contribute to the use of strategies to improve questionnaire response and for participants to return to sites for follow-up in the context of primary care trials.

We conducted a qualitative study with primary care trial personnel to explore the strategies generally used to improve retention in primary care randomised trials and to map these to the results of our Cochrane review[2] .

Methods

Sampling the study population

To explore the use of strategies to improve retention in primary care trials, in-depth face to face interviews were conducted with principal / chief investigators (PIs), trial managers (TMs) and research nurses (RNs) purposively sampled from a sampling frame of primary care

For peer review only - http://bmjopen.bmj.com/site/about/guidelines.xhtml

trials published from 2000-2010. The trials were identified from either the MRC General Practice Research Framework (GPRF) database of clinical research projects, websites of UK primary care research units or from hand searches of trials published between 2009 - 2010 in *Lancet, British Medical Journal, Family Practice* and *British Journal of General Practice*. The trial publications identified spanned different disease areas, research units, and levels of loss to follow-up (Tables 1 and 2) and were stratified by levels of loss to follow-up (<20%, ≥20%), and publication date (2000-2004 and 2005-2010). All PIs, TMs and RNs were identified for recruitment from the list of authors associated with each trial in the sampling frame or through records of trial staff associated with MRC GPRF primary care trial publications. In addition TMs were identified through the Trial Managers Network[11]. TMs recruited in this way had to be associated with a primary care trial published between 2000 - 2010 which was subsequently entered into the sampling frame.

Data collection

Ethics approval was granted by University College London Research Ethics committee (application no 2342/002). Potential interviewees were sent an information sheet and recruited via email. Prior to the interview participants were given study information and asked to sign a consent form. Face-to-face interviews explored everyday retention practices and the factors thought to contribute to retention in primary care trials. If retention strategies found by the Cochrane review (i.e. incentives, communication methods, new questionnaire formats, behavioural, case management and methodological strategies[2]) were not mentioned spontaneously by interviewees, they were asked specifically about these. (Interview schedule Appendix 1). Interviewees were also asked specifically about the role of the ethics approval process on the use of monetary incentives to improve retention in primary care trials.

The interviewee's experiences were not limited to the trial that they were sampled from. The interviews were conducted by one author (VB) in a place convenient to the interviewee they were recorded digitally and took approximately one hour. Transcripts were checked following each interview for flow and usefulness of results and the interview schedule modified accordingly. Appropriate probes were used to allow participants to expand on issues around the use of strategies to improve retention.

Data management and analysis

For peer review only - http://bmjopen.bmj.com/site/about/guidelines.xhtml

We conducted a thematic content analysis. The textual data were managed using Atlas ti 6. A transcript review group was formed with four authors (VB, CV, GR, FS) and was heterogeneous in terms of the members' professional background. Each transcript was reviewed independently by at least two group members who documented the emerging major themes which were then discussed by the group in pre-planned meetings. This group met monthly while data were collected. There was a high degree of convergence in the themes identified.

Data were also analysed iteratively so early results could be incorporated and probed in later interviews to increase the depth of the findings. Labels were agreed and used as broad codes to label textual data associated with major themes. Sub codes were identified discussed and agreed between two authors (FS, VB) and also used to label textual data. Transcripts were subsequently coded by one author (VB). The first two transcripts were checked by another author (FS). A key consideration in coding was to mark data produced spontaneously and that which was specifically asked about, therefore data were coded to take account of the response and the question that prompted that response in order to apply appropriate emphasis on responses. Codes for the six strategies identified by the Cochrane review i.e. *"communication", "incentives", "questionnaires", " methodology", "case management"* and "*behavioural"* were used as a priori codes for the later part of the interview transcripts as participants were asked specifically about the use of Cochrane review strategies.

Textual data relating to retention strategies was subsequently retrieved from a coded database and constantly compared within and across the three groups of trial personnel (i.e. PIs, TMs, RNs) to identify the use of each strategy and to document the reported advantages and disadvantages of each. Any factors thought by the interviewees to contribute to either retention or loss to follow-up were also coded and retrieved for analysis[12;13]. Relevant quotes, representing the interviewee's views, were selected to illustrate the results. Interviews were conducted between August 2010 and May 2011 until no new content or themes emerged.

Results

Fifty four trial personnel were identified for recruitment from 37 primary care trials from England and Scotland that were included in the sampling frame. Eleven of the fifty four invitees declined to participate (PIs (n=10), TMs (n=1)). Seven of these PIs recommended

For peer review only - http://bmjopen.bmj.com/site/about/guidelines.xhtml

another co-author to invite. Fourteen of the fifty four did not respond (PIs (n=10), TMs (n=3), RNs (n=1). Ten PIs, ten TMs and nine RNs were recruited from 23 of the 37 primary care trials identified.

Interviewees were from primary care trials conducted in nutrition, health promotion, neurology, gynaecology, mental health, musculoskeletal, ear nose and throat (ENT), respiratory, and endocrine medicine, as well as from trials of minor medical conditions. Collectively their experiences included trial design, implementation, coordination and data collection at GP practice sites by post, face to face and electronic methods. Trial publications reported loss to follow-up rates between 5% - 39%. Twenty interviewees were from trials with <20% loss to follow-up reported (See Tables 1 and 2). Different types of follow-up were used in these trials: face-to-face follow-up at clinics (n=20), postal questionnaires (n =6), follow-up at home (n=2) or self-completion diaries (n=2). Combinations of follow-up types were also used: clinic visits or postal follow-up (n= 4), telephone and postal follow-up (n=2), register data and follow-up at home (n=1).

The PIs were responsible for the design and implementation of trials, including research governance and ethics approval applications. Nine PIs were GPs with academic roles, and one was a non-clinical senior academic. The TMs were based in academic research units and managed trial data collection via telephone, post, email and SMS text message. TMs also coordinated and monitored data centrally and communicated with trial sites. Six RNs coordinated clinical research at GP practice sites. They collected biomedical and clinical data by telephone and face-to-face interview, and communicated with the coordinating centre and with PI clinicians at sites. Three RNs were based at a primary care research network coordinating centre where they answered clinical data collection queries and monitored data quality.

Results are presented first on the interviewee's experiences of and perspectives on using communication, incentive and new questionnaire strategies, identified by the Cochrane review, to improve primary care trial retention. This is followed by views on other less frequently evaluated strategies identified by the Cochrane review i.e. behavioural, case management, and methodological strategies. Factors thought to contribute to retention and loss to follow-up in primary care trials are also presented.

For peer review only - http://bmjopen.bmj.com/site/about/guidelines.xhtml

The use of retention strategies identified by the Cochrane review

1. Use of incentives

Monetary incentives

Monetary incentives and offers of monetary incentives were mentioned spontaneously by most PIs and TMs as useful strategies to improve response to postal questionnaires. Over half of the PIs interviewed had used monetary incentives given in either cash or voucher format to increase questionnaire return, although there was uncertainty about their effect. The different types used were high street vouchers and mobile phone vouchers, but vouchers were thought of as a burden to administer for trial management teams. Generic vouchers were thought to be more acceptable to all trial participant groups. It was felt that the incentives should be seen by trial participants as a "*thank you*" rather than as payment for participation.

The monetary value of the incentives used was between £5 and £20 and this amount was considered reasonable for adults and children. Larger monetary incentives (>£50) were perceived as coercion or bribery by interviewees across the three groups interviewed. Offers of entry into a prize draw, although seldom used as a strategy to improve questionnaire response, were thought by some interviewees to be a cost effective alternative to giving a monetary incentive.

There was uncertainty among TMs about effective ways to administer incentives during follow-up to optimise questionnaire response. Some gave a small incentive at different follow-up time points e.g. £5 at randomisation and £5 at trial completion. Others sent an incentive with the questionnaire, or on receipt of a completed questionnaire.

TMs and PIs sampled from trials with <20% loss to follow-up appeared to use more strategies to improve retention than those from trials with ≥20% loss to follow-up. For example those in the <20% group gave incentives at different time points to try to keep the participants motivated to return questionnaires. TMs and PIs sampled from trials with ≥20% loss to follow-up seemed more cautious about the use of incentives and some felt participants could feel coerced if incentives were used to improve follow-up.

For peer review only - http://bmjopen.bmj.com/site/about/guidelines.xhtml

Non-monetary incentives

Non-monetary incentive strategies or gifts i.e. pens, certificates of appreciation, key rings and mugs were sometimes used in an attempt to improve both participant recruitment and retention. These were used to market the trial, and in the case of pens, to encourage and remind participants to return completed questionnaires. Interviewees thought that these gifts could be perceived by trial participants as patronising, not useful, or associated with charity fundraising and also as a waste of public money, especially if the participant had altruistic motivations for participation.

> *"I think people are very sensitive about money especially in the public sector, ...if they see something that could be considered wasteful or extravagant, I don't think it would go down too well. Because...... if you get involved in a study that's being funded from public funds and from charity, you know, it's quite an altruistic thing to do, I don't think people are looking to be rewarded for it ....then they see you wasting money on pens and mugs or, I don't think it would go down very well".*
>
> ***Trial manager interview 7***

Some interviewees thought that the money used to finance such non-monetary / gift incentives could be better used to subsidise follow-up visits for participants. Indeed there was consensus amongst interviewees about the importance of reimbursing travel and parking expenses associated with follow-up visits. Some interviewees differentiated this from giving incentives to participants. Others recounted cases where participants did not want to be reimbursed particularly if they had altruistic motivations for participation, and others felt that an offer of money to cover expenses could make participants feel valued. There was a general feeling that this varied by participants' socio-economic circumstances e.g. it was thought that participants from higher socioeconomic groups were less likely to accept a monetary incentive.

> *"We actually gave them ten pounds for their travel costs, once at the beginning and once at the end.. and a lot of them didn't want it, you know, they'd say, oh... And I'd say, well just pop it in your local charity".*
>
> ***Research nurse interview 2***

Ethical approval for the use of incentives

Interviewees across all groups thought it was important that ethics committees sought justification for the use of incentives in trials. They felt that opinion varied among ethics

For peer review only

committees about the use of incentives to improve retention but overall they were now more accepting of the use of small monetary incentives and of reimbursing participants' trial related expenses. Some PIs said that it was helpful to know who was on the ethics committee and that approval to use incentives is generally granted when a clear justification for use is given. Others thought incentives were likely to be approved if they were used to pay participant travel expenses.

2. Use of communication strategies

Communication strategies, for example contact with participants by telephone, letter and email during follow-up, were spontaneously mentioned and routinely used to improve trial retention. RNs and TMs used these as first line retention strategies.

Telephone calls

RNs frequently used telephone calls, to arrange or re schedule trial follow-up appointments or to remind participants to return postal questionnaires. Both RNs and PIs were conscious not to harass participants with too many telephone reminders. TMs thought reminder calls to participants prior to follow-up appointments might increase follow-up attendance. A telephone reminder to a site to remind the RN that a participant is due for a follow-up appointment was also thought to potentially improve retention. PIs did not contact participants by telephone and one thought it would be "*daunting*" for a participant to receive a call from a PI. Nevertheless, PIs were involved in decisions about when to use telephone contact, especially when they thought that participants were fatigued by providing data in diaries or repeat questionnaires.

Letters

Letters were used predominantly by TMs to communicate with trial participants, but there was uncertainty about their effect. Electronic coloured signatures, franked institutional logos placed on outward envelopes, different coloured envelopes, and first class outward post were used to attract the attention of participants.

Letters were usually signed by TMs rather than PIs, the TMs thought that consistency in the signatory was more important than the status of the signatory for improving questionnaire response. Some TMs sent letters to participants in time for weekend delivery, but were unsure whether this influenced response. PIs thought that letters that were personalised, and the tone

For peer review only - http://bmjopen.bmj.com/site/about/guidelines.xhtml

of the letter were important for retention or improving questionnaire return, but that the associated large mail-outs were burdensome for trial staff. Greeting cards were used occasionally to maintain communication with participants, however, interviewees were negative about the impact of these on retention and questionnaire response, with the exception of the use of birthday cards in paediatric trials, and Christmas cards sent to sites from coordinating centres.

> *"I'm routinely suggesting that we send out birthday cards to children. We've done Christmas cards to participants on behalf of the trial team but there's no evidence that it works, but I think that it's a gesture and it's in the right direction, it's better than nothing".*
>
> ***Trial manager interview 27***

There was uncertainty about the effect on retention / response of the different types of postal delivery they use to send trial materials to participants. First class post was used to send trial materials to participants and 2nd class post for questionnaire return. While some TMs and RNs thought that 1st class post was a waste of financial resources, other TMs thought 1st class stamped letters were more likely to be opened by participants. Recorded delivery was occasionally used to send important study-related materials, or to resend questionnaires to non-responders. Some thought recorded delivery was useful to find out if a participant had changed address, but others thought recorded delivery could be inconvenient e.g. if the participant had to collect trial supplies from a post office.

Some TMs noticed that retention improved when questionnaires were sent before clinic visits as this enabled participants to complete the questionnaire at home beforehand. A prepaid envelope for return of postal questionnaires was thought to reduce the cost of postage to participants and the inconvenience of having to buy a stamp. To minimise respondent burden, clear instructions on how to return the questionnaire once it is completed was thought by TMs to contribute to improved postal questionnaire response.

Electronic communication

TMs and RNs used emails to contact participants who preferred this approach. SMS text messaging was used less so but most interviewees thought that it would be useful for communicating with young people. The use of an automated system for texting appointment reminders similar to that used by the NHS for clinic appointments was thought to be a

For peer review only - http://bmjopen.bmj.com/site/about/guidelines.xhtml

potentially useful way to improve trial retention. However one RN reported that the reminders sent via the system did not improve clinic attendance.

Newsletters

Most interviewees thought newsletters were useful for keeping participants and site clinicians informed about trial recruitment, retention, and general trial news. Others thought these were less useful and could bias trial results by contaminating and confounding the results of the *treatment as usual* group. The frequency with which newsletters were sent to participants varied from fortnightly to annually, and depended on the condition / disease, intervention, and length of follow-up. Some PIs thought having trial-specific information and answers to frequently asked questions as a resource on a website could benefit retention.

3. Use of new questionnaire formats

None of the interviewees spontaneously mentioned modifying a follow-up questionnaire to improve response or retention, although some PIs and TMs had used shorter questionnaires for non-responders. When asked specifically about the impact of questionnaire length on retention, interviewees expressed a preference for shorter questionnaires, which they thought increased response. Interviewees also thought that long questionnaires administered frequently led to "*questionnaire fatigue*", and some thought that dividing long questionnaires into several short questionnaires could be useful to reduce the burden of data collection on participants. However, some PIs thought that if the questionnaire content was of interest to the participant, then the length of the questionnaire may not affect response. TMs and PIs thought the involvement of patient representatives in the design and pre-test of questionnaires could improve retention. Many suggestions were also offered for improving questionnaire design in order to increase questionnaire response (Figure 1).

How a questionnaire was administered and by whom was also thought to affect questionnaire response and retention. Some PIs and TMs thought that if data were collected at the clinic site by a nurse rather than by post this could increase response.

4. Use of other strategies to improve retention

For peer review only

For peer review only - http://bmjopen.bmj.com/site/about/guidelines.xhtml

Other strategies identified by the Cochrane review were not routinely used or mentioned spontaneously by interviewees as ways to improve retention. Opinions were mixed about the usefulness of a) a behavioural strategy where participants are given information about goal setting and time management to facilitate successful trial completion; b) methodological strategies where a blind trial design was compared to an open / unblind trial design; c) case management where trial assistants manage participant follow-up, by arranging services to enable participants to keep trial follow-up appointments.

Behavioural strategies were described to interviewees as *"Arranging workshops to give participants information about goal setting and time management"* for example in the context of an exercise behaviour change intervention. When asked specifically about the use of such a strategy, interviewees were generally negative. Such strategies were discussed in the following ways: "*strange*", "*preachy", "condescending*", "*never came across it", "would never think of doing it*", *I'd be surprised if it worked*" and "*would need evidence that it worked*". Some thought that a behaviourr change retention strategy could not be used because it may contaminate a behaviour change trial intervention and therefore may affect the generalisability of trial results.

Although the interviewees were familiar with the concept of blind and open trials, they were not aware of the use of either design as a strategy to improve trial retention. Interviewees felt that using an open trial design to improve retention could bias results, particularly as blinding is used to reduce bias in trials. They also felt that a blind trial design could not be applied to therapist-led behavioural interventions

One TM had used case management to improve trial retention. This involved the TM arranging services to enable participants to attend trial follow-up appointments. The interviewees generally thought that case management could improve trial retention with some groups e.g. the elderly, disabled and participants with low incomes but they also thought this could create dependence on the service. The impact of this strategy on retention was thought by some to depend on the personality of the case manager.

Factors thought to contribute to trial retention

Participant's time

For peer review only

A key factor thought to contribute to retention was reducing the burden of follow-up for participants in terms of the length of time they spend in follow-up activities, and the type of data collected (e.g. biomedical specimens, long questionnaires). More convenient ways to collect outcome data, e.g. online data collection were thought to be more effective for retention.

> *"The burden of activity on the patients, how easy it is for them, how long it's going to take, how intrusive in terms of blood samples or answering huge long questionnaires, it's partly the complexity as well, if it's very simple for them to do, often you just want very simple data back from people, alive or dead, or specific complications, if it can be done either with a, click of a button by sending a text or an email to people or just …reply paid cards so you're just getting the data back from them, ..the other thing is…… to do electronic follow-up as much as possible so you don't actually have to bother the patient, you have their consent to access their electronic records and either their Primary care data can be interrogated …".*
>
> ***Principal investigator interview 4***

The time participants spend getting to, from, and at follow-up visits, giving participants adequate time at follow-up visits, and ensuring that participants were not kept waiting too long for follow-up appointments were all considered important factors for retention.

> *"The cost to the patient in time and effort to get to the surgery,…what time these appointments would be. How long they would take, how much effort it would take to get the patient… and also, if it's a research project, I always feel that patients shouldn't wait, …it's up to the nurse to make her appointments work so that she doesn't have anybody waiting there for any length of time,*
>
> ***Research nurse interview no 1***

Engaging with participants

The recruitment visit was seen as an important opportunity to engage with participants and to give key information about trial follow-up procedures.

> *"It [the recruitment visit] sets the stage for the participants relationship with the trial …………. it's important to get that right, right from the start…. that affects whether people think it's worthwhile coming back…"*
>
> ***Trial manager interview 26***

For peer review only - http://bmjopen.bmj.com/site/about/guidelines.xhtml

Establishing a rapport with participants that continues throughout follow-up was also thought to contribute to retention.

> *"At the end of the day it's how the nurse and GP …….establish a rapport and a relationship with the participant,… that is the key issue in keeping participants in a study".*
>
> ***Research nurse interview 12***

Good rapport was thought to be maintained through continuity of trial staff at trial sites for follow-up visits. Some TMs thought that having "*personal*" or "*good*" communication with participants and within the research team contributed to participants returning to sites for trial follow-up.

> *"I think it is communication at all levels; so the nurse who interfaces with the trial management team behind the coordinating centre, … listening to the participant's views …and … feeding them back, ……. a two-way process. Because we're this side doing the research, we have to listen … so yes, … communication and understanding [the] population that you're working with".*
>
> ***Trial manager interview 13***

TMs use combinations of different communication strategies to increase response to postal questionnaires e.g. combining postal and telephone methods for non-responders.

> "*We …..mak(e) sure that we have [communication] strategies in place to get as many …..responses to our postal questionnaires …we send out the questionnaire…, if we don't hear back ….we will ring up to say ….''Could you possibly send it back to us?'' If we still don't hear …we'll send .. another copy … if we don't have a response from that copy then we'll ring them again…if we still don't hear we've telephoned …again to collect a set of core outcomes to get a response…………"*
>
> ***Trial manager interview 14***

Some interviewees across all groups mentioned the impact of trial staff personalities on trial retention. Interviewees thought RNs needed to have a "*flexible approach*" and to "*be sympathetic*" toward participants, while TMs needed to be "*competent*", "*personable*", "*persistent*", "*enthusiastic*", and "*good communicators*".

> "*If you're enthusiastic, can give the information to the participants and…[are] sympathetic to …. queries, always being accommodating,[and] getting back to them if they have any queries …being absolutely spot on with, so that they don't feel they've been let down or you haven't been bothered to get back to them."*

For peer review only - http://bmjopen.bmj.com/site/about/guidelines.xhtml

Research nurse interview 5

"I think there's a paradox here because they [trial managers] need to be obsessional about detail but they also need to be relaxed and flexible in their response to different situations ....and problems that come up. That's asking quite a lot, I think. So you know it's great if you find somebody who's good at it.....Or be very kind hearted. And prepared to work out of hours!"

Principal investigator interview 25

Perceived benefit of trial participation

Most interviewees thought that if trial participants perceived an extra benefit from participating in a trial that this would contribute to retention particularly if they are being monitored for disease progression.

"In studies where (there) is ... constant monitoring ....they don't mind giving..... samples again and again as part of the trial..... But I ...think .... if they can see some benefit then they'll do it but if they can't or if they've already got that benefit from giving the sample at the first time point they might not want to do it again and again"

Trial manager interview 10

Some of the benefits identified from participation were extra health monitoring for example: "*ECG" "BP"* and *"cholesterol"* as well as the extra attention from staff. However, these perceived benefits were thought to be trial dependent.

Participant altruism

Participant altruism was also thought to improve retention where trial participants were committed to a trial for the better good of society:

"...They [participants] feel as though they want to do good because they often, a lot of them say "oh it's payback time", they think the NHS has been good to them so they want to give something back".

Trial manager interview 7

Tailoring retention strategies

Tailoring specific retention strategies to enable different trial population groups to either return questionnaires or return to sites for follow-up was thought to improve retention. In mental health trials for example, some interviewees found that participants may wish to be followed-up at home rather than at a clinical site.

For peer review only

For peer review only - http://bmjopen.bmj.com/site/about/guidelines.xhtml

"Depending on the trial and the length of the trial and your client group …you would have a completely different strategy for… the fifteen to twenty-four year olds, to something… where you're looking at the seventy-five plus….. you do sort of tailor it [the strategy] to your group."

Research Nurse interview 2

Factors thought to contribute to loss to follow-up

Several age groups were highlighted as challenging for trial retention, for example, teenagers wishing to opt out of a trial when they reach the age of consent; working mothers juggling school runs with work time, and elderly participants who have either lost their independence or are involved with extended family caring activities. Young men with other interests were also thought to be challenging to retain in trials

"A young girl whose parents had agreed to be in the trial and then, you know, she didn't want to and then decided herself she was going to withdraw. And, you know, I can see the potential for that sort of thing if it's a trial in the sort of pre-teen, teen group that,... they might well change their mind….more interesting things going on in their lives, they've got more after school activities or they've got exams or they've got whatever. <u>And</u> coupled with being more independent and wanting to assert their own ideas".

Principal investigator interview 28

"In trials with older people, people will die, people will get frailer and maybe be institutionalised and not be able to continue or not want to continue for those sort of reasons".

Principal investigator interview 28

"There are all the usual things, we assume that older people don't move, there's a astonishingly high level of mobility in the older population. So lost to follow-up can occur just because people say "right, I'm going to go and live in…………..." or "I'm going to go and live near my daughter" or "I've off to ………..for the winter". [They] do… a lot of child care, grandparental child care and sorting out their children's divorces and so on and so they get distracted and drop out completely through distraction".

Principal investigator interview 21

Some interviewees thought that if participants did not perceive a benefit from participation they might not return for their follow-up visit. Feelings of guilt and shame with not having

For peer review only - http://bmjopen.bmj.com/site/about/guidelines.xhtml

achieved positive outcomes in a trial were thought to contribute to loss to follow-up in some disease areas / conditions e.g. in treatment for obesity trials, if participants were not losing weight they might not return to be weighed perhaps due to the shame and stigma associated with not achieving their expected aims. Retention in trials involving behaviour change was also thought to be problematic if change targets were not achieved by participants. For example, interviewees were generally in agreement that retention can be problematic in trials involving participants with a mental health issue.

> *"In nutrition studies we're very much bound by looking at healthy individuals to begin with. So from that perspective they may certainly be less motivated than an individual participating in a drug trial who has a severe or debilitating condition that, you know, they want to try and do something about".*
>
> ***Trial manager interview 15***

> *"For smoking cessation or weight management or any of these things where certain behaviour is expected or wanting to be demonstrated. If they're not achieving it they are more likely to just drop out"*
>
> ***Research nurse interview 9***

> *"Bi-polar groups, a lot of them find it very, very difficult going to an NHS facility".*
>
> ***Trial manager interview 17***

> *"it depends on what area of medicine it is, for instance, if you're dealing with people with psychiatric illness, there's always a problem with attendance, whether that's within the surgery or whether that's within research".*
>
> ***Research nurse interview 1***

The interviewees felt that participants with chronic diseases may have more interest in the treatment of their condition and return for follow-up visits. Others thought healthy volunteers may drop out of trials because of lack of interest in the trial.

Some interviewees reported that the working environment at general practice trial sites might impact upon retention. It was thought to be important to have a staff member at the site to "*champion*" the study. Part-time RNs reported working in isolation and sometimes struggled to find a consulting room for follow-up visits which restricted the availability of flexible follow-up appointment times to offer to participants.

> *"The last study that I worked on, ..I struggled to find .. a room, the practice were incredibly accommodating, and really wanted me to have a room, but it just is very,*

For peer review only - http://bmjopen.bmj.com/site/about/guidelines.xhtml

very tight. …it has been, a major issue for me…It was affecting follow up for me because I was not always able to have a room when I was available, that ….would have been potentially available for participants, so it just narrowed down the opportunities for me to offer to participants to come in"

Research nurse interview 5

"*if somebody genuinely did want to make the trial work then it was possible to make it work. ………………..it needed somebody to actually manage the trial within that practice. The practices in which we had success I felt that we had a champion there who was fighting our corner for us"*

Trial manager interview 27

"*the other thing is practice awareness, you know, because you said it's a big practice and if somebody comes in and hasn't got the sort of clock-in bit straight and they go to the desk and say, I've come to see X about this trial and, you know, new receptionist knows nothing. That can be quite off-putting so as long as everybody's engaged …and everybody knows about it".*

Research nurse interview 2

Discussion

Statement of principal findings

Based on in-depth interviews with primary care trial personnel we identified that incentive, communication and questionnaire strategies are used to try to increase retention in primary care trials. Making efficient use of participant time and appropriate engagement with participants were also thought to increase retention. Interviewees thought that participant's perceptions of benefits from participation; the age of participants e.g. young men and teenagers; the disease area / condition e.g. mental illness and behavioural problems; and the research environment in which the trial was conducted e.g. availability of consulting rooms for follow-up appointments all contributed to loss to follow-up in trials.

Strengths and weaknesses

Research on strategies to improve retention of participants in primary care trials is limited and commonly grouped with recruitment strategies [14;15]. Trial retention is important because loss to follow-up can lead to incomplete data for the primary outcome, bias results and impact on the generalisability of trial findings. This study is the first to explore the use of retention

For peer review only - http://bmjopen.bmj.com/site/about/guidelines.xhtml

strategies in UK primary care trials and the factors associated with retention. Interviews were conducted with researchers experienced in design, leadership, management, and implementation of UK primary care trials, many of whom reported that prior to this study that they had not thought deeply about loss to follow-up or strategies used to overcome this. With the focus by funders on recruitment rather than retention targets, this study specifically raises awareness of the complexity of retention in primary care trials and the many strategies and factors used to maximise retention. Although the thoughts and experiences of trial participants were not captured to further explore the barriers and facilitators to retention the results show that trial personnel have an understanding of the challenges participants encounter to stay in trial follow-up. It is unclear how generalisable the results are to trials outside of primary care.

Meaning and implications for the use of retention strategies

These results enhance the interpretation and implementation of the results of our Cochrane review on ways to improve retention in trials[2]. Together, these two studies inform the future use of effective strategies to improve retention in primary care trials.

There was general agreement that small monetary incentives were likely to be viewed favourably by ethics committees and led to increased questionnaire response, in agreement with the findings of the Cochrane Review[2]. Although there was uncertainty among those interviewed about whether monetary incentives should be given up front or offered on receipt of data, the Cochrane review showed that either approach increased postal questionnaire response[2].

Scepticism among interviewees about non-monetary incentives and their value was also supported by the Cochrane Review which did not find them to be effective. Trialists may therefore need to reconsider the use of this type of incentive.

A range of different communication strategies were discussed by interviewees. Although trialists routinely preferred First class post, and the use of enhanced letters, the Cochrane Review found no evidence to support either approach. Therefore, 2nd class post and standard letters can probably be used for postal correspondence with primary care trial participants, with little impact on loss to follow-up.

For peer review only

Trialists had mixed opinions on the use of recorded delivery, nevertheless recorded delivery of questionnaires compared with a telephone reminder was found to be effective for improving questionnaire response[2;5]. To avoid any inconvenience associated with the use of this strategy, preplanning the delivery time with the participant with a reminder to expect a recorded delivery of trial materials is recommended if this strategy is to be used in future trials.

Different types of reminders and alternative questionnaires are routinely used by primary care researchers to improve trial follow-up, however there is no clear evidence that reminders and prompts in addition to the standard trial follow-up procedures are beneficial to retention[2]or to improving questionnaire response. Similarly, there is no evidence to suggest better response rates to shorter questionnaires[2].

Furthermore, interviewees were particularly negative toward the use of behavioural strategies and there was no effect found for these in our Cochrane review[2]. Case management, although seldom used, was thought to be potentially useful by interviewees, however, there is no evidence that this is effective[2]. The interviewees also thought that blinding participants to their allocated intervention would improve retention. This is in contrast to findings from the Cochrane review which found that an open trial design was more effective than a blind design in one trial[2;4]. Avenell argues that double blind trials do not reflect usual health care procedures, and that open trials, where the intervention is compared to no treatment or usual treatment, could give a better measure of the differential effects in normal care settings[4]. The open trial design, if used as a stand-alone strategy to improve retention, would need to be considered in the context of the overall aims and objectives of the trial. Further evaluation is needed in different trial contexts and settings if this retention strategy is to be adopted.

Several other factors and ways to improve retention were identified that were not covered in our Cochrane review[2]. Some of these such as relationships and communication between trial staff and trial participants are clearly more difficult to design interventions for and to evaluate. The factors identified indicate a need to train and support trial staff in ways to improve trial retention. It seems clear from this qualitative study that trial staff were oriented to patient concerns and needs (as they perceived them) and had a high level of empathy with participants.

For peer review only

Integrating qualitative research with Cochrane reviews

Integrating the results of the qualitative study and the Cochrane review helped us understand the different experiences primary care researchers have with the strategies identified by our Cochrane review[2]. The qualitative results support some of the results of our Cochrane review and demonstrate the potential transferability of the different effective retention strategies to trials conducted in primary care. For example incentives were employed in different ways to try to improve responses to postal questionnaires. The results have also helped identify other factors that might promote retention such as building a rapport with trial participants, and potential barriers like finding a consulting room for follow-up appointments. This mixed methods approach could be replicated in research contexts apart from primary care to determine how appropriate and useful the strategies identified by the Cochrane review are in practice.

While the absolute effects of effective strategies identified by the Cochrane review are modest[2], this qualitative study highlights that multiple strategies are often used to try to achieve maximum retention in primary care trials. Furthermore it suggests that clinical trialists should consider other factors that may affect retention, such as building a rapport with participants and minimising the impact of trial follow-up on their daily lives. Consideration should also be given to the characteristics of the intervention, participants and trial personnel when choosing effective strategies identified by our Cochrane review to improve trial retention[2].

Future research

It was clear from the interviews that there is a move toward contacting participants by email and a desire to use text reminders when automated facilities for these become available for use in randomised trials. These new strategies would need evaluation in future trials.

Further research is also needed to identify potential barriers and facilitators to follow-up in trials e.g. with trial participants from different social, economic, age and disease groups. The results would be useful for primary care researchers and would help target and tailor strategies to meet the needs of different population groups to keep them specifically engaged in trial follow-up[16].

Conclusion

For peer review only

For peer review only - http://bmjopen.bmj.com/site/about/guidelines.xhtml

Qualitative research can help to interpret the results of systematic reviews by telling us what researchers do or think happens in research practice. These findings provide a deeper understanding of the factors that may facilitate retention such as rapport with participants and respect for their time, and highlights the participant and environmental characteristics that can limit the implementation of effective strategies. The use of small monetary incentives to improve questionnaire response is acceptable however a reconsideration of the use of non-monetary incentives, and certain communication strategies including 1st class post is needed. A similar exploration of barriers and facilitators to retention in other research contexts would help identify ways to improve retention in trials conducted in other research contexts.

For peer review only

Article Summary

Article focus

High levels of retention in trials are important to maintain power and minimise bias. Strategies to improve retention are widely used in trials but without evidence of effect. Strategies thought to improve retention in UK primary care randomised trials have not been well documented or evaluated. This study explores the use, in UK primary care trials, of strategies to improve retention.

Key messages

Small monetary incentives, non-monetary incentives and communication strategies are used in primary care trials to improve retention. Behavioural, methodology and case management strategies were not used. Small monetary incentives and communication strategies were thought to be useful for retention and case management thought to be potentially useful. Interviewees were negative about the use of behavioural, methodology and non-monetary incentive strategies.

Factors thought to contribute to retention are: effective communication, participant altruism and perceived benefits of participation for participants, burden reduction, personality and flexibility of trial staff.

Factors thought to contribute to loss to follow-up are: participant age, disease/condition and research environment.

Strengths and limitations

This study is the first to explore the use of retention strategies in UK primary care trials and the factors associated with retention and loss to follow-up. Interviews were conducted with researchers experienced in UK primary care trials, many of whom had used strategies to improve retention.

A limitation is that the thoughts and experiences of trial participants have not been gathered to further explore the barriers and facilitators to retention in primary care trials.

For peer review only - http://bmjopen.bmj.com/site/about/guidelines.xhtml

For peer review only

Acknowledgements

We would like to thank all twenty nine interviewees who gave of their time and thoughts on the complexities of trial retention in primary care.

Statement of funding and competing interests

All authors have completed the ICMJE uniform disclosure form and declare no competing interests. This project was funded by the Medical Research Council Population Health Sciences Research Network grant number PHSRN 30

For peer review only

For peer review only - http://bmjopen.bmj.com/site/about/guidelines.xhtml

Reference List

(1) Janson SL, Alioto ME, Boushey HA. Attrition and Retention of Ethnically Diverse Subjects in a Multicenter Randomized Controlled Research Trial. *Control Clin Trials* 2001; 22(6, Supplement 1):S236-S243.

(2) Brueton VC, Tierney J, Stenning S, Nazareth I, Meredith S, Harding S et al. Strategies to improve retention in randomised trials . *Cochrane Methodology Group* 2013; in press.

(3) Sutherland HJ, Beaton M, Mazer R, Kriukov V, Boyd NF. A randomized trial of the total design method for the postal follow-up of women in a cancer prevention trial. *Eur J Cancer Prev* 1996; 5(3):165-168.

(4) Avenell A, Grant AM, McGee M, McPherson G, Campbell MK, McGee MA et al. The effects of an open design on trial participant recruitment, compliance and retention - a randomized controlled trial comparison with a blinded, placebo-controlled design. *Clinical Trials* 2004; 1(6):490-498.

(5) Tai SS, Nazareth I, Haines A, Jowett C. A randomized trial of the impact of telephone and recorded delivery reminders on the response rate to research questionnaires. *J Public Health* 1997; 19(2):219-221.

(6) McColl EM, Eccles MPM, Rousseau NSB, Steen INP, Parkin DWD, Grimshaw JMP. From the Generic to the Condition-specific?: Instrument Order Effects in Quality of Life Assessment. [Article]. *Med Care* 2003; 41(7):777-790.

(7) Cockayne S, Torgerson D. A randomised controlled trial to assess the effectiveness of offering study results as an incentive to increase response rates to postal questionnaires [ISRCTN26118436]. *BMC Medical Research Methodology* 2005; 5(1):34.

(8) Man MS, Tilbrook HE, Jayakody S, Hewitt CE, Cox H, Cross B et al. Electronic reminders did not improve postal questionnaire response rates or response times: a randomized controlled trial. *J Clin Epidemiol* 2011; 64(9):1001-1004.

(9) Dangour AD, Allen E, Elbourne D, Fasey N, Fletcher AE, Hardy P et al. Effect of 2-y n-3 long-chain polyunsaturated fatty acid supplementation on cognitive function in older people: a randomized, double-blind, controlled trial. *Am J Clin Nutr* 2010; 91(6):1725-1732.

(10) Williamson I, Benge S, Barton S, Petrou S, Letley L, Fasey N et al. Topical intranasal corticosteroids in 4-11 year old children with persistent bilateral otitis media with effusion in primary care: double blind randomised placebo controlled trial. *BMJ* 2009; 339(dec16_1):b4984.

(11) Trial Managers Network. 2013.
Ref Type: Online Source

For peer review only

(12) Pope C, Ziebland S, May N. Analysing qualitative data. In: Catherine Pope, Nicolas May, editors. Qualitative Research in Health Care. second ed. London: BMJ Publishing Group; 2000. 75-88.

(13) Farmer T, Robinson K, Elliott SJ, Eyles J. Developing and Implementing a Triangulation Protocol for Qualitative Health Research. *Qual Health Res* 2006; 16(3):377-394.

(14) Leathem C, Cupples M, Byrne M, O'Malley M, Houlihan A, Murphy A et al. Identifying strategies to maximise recruitment and retention of practices and patients in a multicentre randomised controlled trial of an intervention to optimise secondary prevention for coronary heart disease in primary care. *BMC Medical Research Methodology* 2009; 9(1):40.

(15) Graffy J, Grant J, Boase S, Ward E, Wallace P, Miller J et al. UK research staff perspectives on improving recruitment and retention to primary care research; nominal group exercise. *Fam Pract* 2009; 26(1):48-55.

(16) Kimmel SE, Troxel AB. Novel incentive-based approaches to adherence. *Clin Trials* 2012; 9(6):689-695.

For peer review only

For peer review only - http://bmjopen.bmj.com/site/about/guidelines.xhtml

Use of strategies to improve retention in primary care randomised trials: a qualitative study with in-depth interviews

Brueton V

Research Scientist, Meta-analysis Group
MRC Clinical Trials Unit
Aviation House
125 Kingsway
London WC2B 6NH
e-mail: vcb@ctu.mrc.ac.uk

Stevenson F

Senior Lecturer
UCL Research Department of Primary care and Population Health
Royal Free and University College Medical School
Rowland Hill Street
London
NW3 2PF

Vale CL

Senior Scientist Meta-analysis Group
MRC Clinical Trials Unit
Aviation House
125 Kingsway
London WC2B 6NH

Stenning SP

Senior Statistician
MRC Clinical Trials Unit
Aviation House
125 Kingsway
London WC2B 6NH

Tierney JF

Senior Scientist Meta-analysis Group
MRC Clinical Trials Unit
Aviation House
125 Kingsway
London WC2B 6NH

Harding S

Program leader: Ethnicity and Health

For peer review only

BMJ Open qualitative paper submitted Version 3 VB 4.11.13

MRC Social & Public Health Sciences Unit
University of Glasgow
4 Lilybank Gardens
Glasgow G12 8RZ

Nazareth I

Professor of Primary Care
PRIMENT Clinical Trials Unit
Research Department of Primary care and Population Health
UCL Medical School
Rowland Hill Street
London
NW3 2PF

Meredith S

Deputy Director
MRC Clinical Trials Unit
Aviation House
125 Kingsway
London WC2B 6NH

Rait G

Clinical Senior Lecturer
PRIMENT Clinical Trials Unit
Research Department of Primary care and Population Health
UCL Medical School
Rowland Hill Street
London
NW3 2PF

For peer review only

For peer review only - http://bmjopen.bmj.com/site/about/guidelines.xhtml

BMJ Open qualitative paper submitted Version 3 VB 4.11.13

Abstract

Objective

To explore strategies used to improve retention ~~identified by a Cochrane review,~~ in ~~UK~~ primary care randomised ~~controlled~~ trials.

Design

Qualitative in-depth interviews and thematic analysis.

Participants

Twenty nine UK primary care chief and principal investigators, trial managers and research nurses.

Methods

In-depth face to face interviews.

Results

Formatted: Not Highlight

Primary care researchers ~~described~~ use~~ing~~ ~~various~~ incentive~~s~~ and communication strategies to improve retention in trials, but were unsure of their effect. Small monetary incentives were used ~~in an attempt~~ to increase response to postal questionnaires. Non-monetary incentives were used although there was scepticism about the impact of these on retention. Nurses routinely used telephone communication to encourage participants to return for trial follow-up. Trial managers routinely used 1st class post to try to increase questionnaire response. Shorter questionnaires were used~~sent to non-responders~~, and suggestions offered for improving questionnaire design. Interviewees thought that an open trial design could lead to biased results and were ~~generally~~ negative about using~~the use of~~ behavioural strategies to improve retention.

There was consensus among the interviewees that effective communication and rapport with participants, participant altruism, respect for participant's time, flexibility of trial personnel and appointment schedules, and trial information improve retention. Participant age, e.g. teenagers, disease type and the work environment at trial sites were thought to contribute to loss to follow-up.

For peer review only

For peer review only - http://bmjopen.bmj.com/site/about/guidelines.xhtml

BMJ Open qualitative paper submitted Version 3 VB 4.11.13

Conclusions

Integration of the findings of this qualitative study with the results of the Cochrane review ~~in-depth exploration of the applicability of the Cochrane review~~ allowed us to reflect on the gap between the evidence from the Cochrane review and how trialists behave in practice. Trialists describe acting without evidence from the literature and from experience of what works. The use of small monetary incentives to improve questionnaire response is ~~effective and~~ acceptable. A reconsideration of the use of non-monetary incentives, and some communication strategies is needed. A similar exploration of barriers and facilitators to retention in other research contexts would help identify ways to improve retention in trials conducted in other research contexts.

Formatted: Not Highlight

Formatted: Not Highlight

For peer review only

For peer review only - http://bmjopen.bmj.com/site/about/guidelines.xhtml

BMJ Open qualitative paper submitted Version 3 VB 4.11.13

Introduction

Retention in primary care randomised controlled trials~~trials (RCTs)~~ conducted across different disease areas and communities can be challenging. Inadequate retention can reduce the power of a trial and introduce bias, particularly if drop-out differs across trial arms. Reasons for loss to follow-up can include a change in the participants' location, withdrawal from treatment and / or loss of commitment to the trial, for example due to complicated treatment regimens[1]. A Cochrane review of strategies to improve retention in trials demonstrated~~12. A Cochrane review of strategies to improve retention in RCTss demonstrated~~ that adding a monetary incentive and offering higher valued monetary incentives increased postal and electronic questionnaire response[2]. ~~21.~~ Questionnaire response was also increased by recorded delivery of questionnaires, a "package" of postal communication strategies known as the Total Design Method ~~and a~~ (TDM) [3]and an open trial design[4], although these were based on the results of single ~~RCTs~~ trials. The evidence of an effect for shorter questionnaires and questionnaires relevant to the disease / condition was less clear. Also, there was no good evidence that the following strategies improved retention: adding or offering a non-monetary incentive, communication strategies (including use of first class mail), behavioural motivational strategies, new questionnaire formats and case management.

~~Primary care RCTs often require participants to return to sites for clinical outcome measurement for example, assessment of disease progression~~[3;43,4]~~. However, in the Cochrane review, there were few evaluations of strategies to increase this type of retention, and O~~Only six of the 38 retention trials~~RCTs~~ included in the Cochrane review were embedded in UK primary care trials~~.~~ and all ~~Rather the included retention RCTs~~ targeted improving response to postal questionnaires in order to improve retention[5-85-8]. However, primary care trials also require participants to return to sites for clinical outcome measurement to assess disease progression[9;10]. There could be important factors that contribute to the use of strategies to improve questionnaire response and for participants to return to sites for follow-up in the context of primary care trials. ~~Moreover, there may be barriers to using strategies to improve retention and in particular those identified by the Cochrane review.~~

We conducted a qualitative study with ~~staff involved in~~ primary care ~~RCTs~~trial personnel to explore the ~~use of of~~ strategies ~~strategies,~~ generally used to improve retention in primary care randomised trials and to map these to the results of our Cochrane review[2] ~~including those~~

Formatted: Normal, Line spacing: 1.5 lines

Formatted: Superscript

Formatted: Superscript

Formatted: Font: 12 pt, Not Highlight

Formatted: Font: 12 pt, Not Highlight

Formatted: Font: 12 pt, Not Highlight

Formatted: Font: 12 pt, Not Highlight

For peer review only - http://bmjopen.bmj.com/site/about/guidelines.xhtml

~~identified by a Cochrane review~~. ~~, to improve RCTs . The aim was to explore the acceptability, feasibility and use of the strategies identified by the Cochrane review, in primary care RCTs. A secondary aim was to identify factors thought to contribute to retention in the context of primary care RCTs.~~

Methods

Sampling the study population

To explore the use of strategies to improve retention in primary care trials~~RCTs~~, in-depth face to face interviews were conducted with principal / chief investigators (PIs), trial managers (TMs) and research nurses (RNs) purposively sampled from a sampling frame of primary care trials~~RCTs~~ ~~. A sampling frame was established using primary care RCTs~~ published from 2000-2010. The trials ~~that~~ were identified from either the MRC General Practice Research Framework (GPRF) database of clinical research projects, websites of UK primary care research units or from hand searches of trials~~RCTs~~ published between 2009 - 2010 in *Lancet, British Medical Journal, Family Practice* and *British Journal of General Practice*. The trial~~RCT~~ publications identified spa~~n~~nned different disease areas, research units, and levels of loss to follow-up (Tables 1 and 2) and were stratified by ~~loss to follow-up~~ levels~~of~~ of loss to follow-up (<20%, ≥20%), and ~~the date of~~ publication date (2000-2004 and~~,~~ 2005-2010). All PIs, TMs and RNs were identified for recruitment ~~to the qualitative study~~ from the list of authors associated with each trial~~RCT publication~~ in the sampling frame~~. TMs and RNs listed as co-authors were also identified in this way. TMs and RNs not listed as co-authors were identified for recruitment~~ or through ~~records held by the MRC GPRF~~ records of trial staff associated with MRC GPRF primary care trial~~RCT~~ publications. In addition ~~we recruited~~ TMs were identified through the Trial Managers Network[11] ~~9.~~ TMs recruited in this way had to be associated with a primary care ~~RCT~~ trial published between 2000-2010 which~~that~~ was subsequently entered into the sampling frame.

Data collection

Ethics approval was granted by University College London Research Ethics committee (application no 2342/002). Potential interviewees were sent an information sheet and recruited via email. Prior to the interview participants were given study information and asked to sign a consent form. Face-to-face interviews explored ~~the use, , of strategies to improve retention and the use of the six types of strategies identified by the Cochrane review in primary care~~

Formatted: Font: 12 pt, Not Highlight

Formatted: Font: 12 pt, Do not check spelling or grammar, Superscript

Formatted: Font: 12 pt, Check spelling and grammar, Not Superscript/ Subscript

Formatted: Font: 12 pt, Not Highlight

For peer review only

~~RCTs (Interview schedule Appendix 1). Initially interviewees were asked about~~ everyday retention practices and the factors ~~that they~~ thought to contribute~~contributed~~ to retention in primary care trials~~RCTs~~. If retention strategies found by the Cochrane review (i.e.- incentives, communication methods, new questionnaire formats, behavioural, case management and methodological strategies[2]) were not mentioned spontaneously by interviewees, they~~They~~ were ~~then~~ asked specifically about the~~se~~m. ~~strategies six types of which strategies identified are~~~~were: incentives, different means of communication methods, new questionnaire formats, behavioural, case management and methodological strategies~~[2,21]. (Interview schedule Appendix 1) ~~(Interview schedule Appendix 1).~~ Interviewees were also asked specifically about the role of the ethics approval process on the use of monetary incentives to improve retention in primary care trials.

The ~~interviewees~~interviewee's experiences were not limited to the trial that they were ~~were asked to consider RCTs generally when answering questions rather than focusing solely on the trial that they were~~ sampled from. ~~Interviewees were also asked specifically about the role of the ethics approval process on the use of monetary incentives to improve retention in primary care RCTs.~~ The interviews were ~~approximately one hour long, were~~ conducted by one author (VB) in a place convenient to the interviewee~~, and~~ they were recorded digitally and took approximately one hour. ~~The interviews were developed iteratively.~~ Transcripts were checked following each interview ~~during data collection~~ for flow and usefulness of results and the interview schedule modified accordingly. Appropriate probes were used to allow participants to expand on issues around the use of strategies to improve retention.

Data management and analysis

We conducted a thematic content analysis. ~~All transcripts were reviewed and validated by at least two authors (CV, FS, GR, VB). They for the interview technique to: a) confirm that the interviewees understood and were able to answer the questions asked during interview b) that leading questions were avoided and c) that appropriate probes were used to allow the participants to expand on issues around the use of strategies to improve retention.~~ The textual data were managed using Atlas ti 6. A transcript review group was formed with four authors (VB, CV, GR, FS)~~,~~ and was heterogeneous in terms of the members' professional background. Each transcript was reviewed independently by at least two group members ~~of~~

Field Code Changed

Formatted: Font: 12 pt, Do not check spelling or grammar, Superscript

Formatted: Font: 12 pt, Not Highlight

Formatted: Font: 12 pt, Not Highlight

Formatted: Font: 12 pt, Not Highlight

Formatted: Line spacing: 1.5 lines

Formatted: Not Highlight

Formatted: Not Highlight

Formatted: Font color: Auto

Formatted: Font color: Auto

For peer review only - http://bmjopen.bmj.com/site/about/guidelines.xhtml

BMJ Open qualitative paper submitted Version 3 VB 4.11.13

~~the transcript review group~~ who documented the ~~. E~~emerging major themes ~~were documented independently by each member and~~which were then discussed by the group in pre-planned meetings. This group met monthly while data were collected. There was a high degree of convergence in the themes identified~~.~~.

Data were also analysed iteratively so early results could be incorporated and probed in later interviews to increase the depth of the findings. Labels were agreed and used as broad codes to label textual data associated with major themes. Sub codes were identified discussed and agreed between two authors (FS, VB) and also used to label textual data. Transcripts were subsequently coded by one author (VB). The first two transcripts were checked by another author (FS). A key consideration in coding was to mark data produced spontaneously and that which was specifically asked about, therefore data were coded to take account of the response and the question that prompted that response in order to apply appropriate emphasis on responses. Codes for the six strategies identified by the Cochrane review i.e. *"communication", "incentives", "questionnaires", " methodology", "case management"* and "*behavioural"* were used as a priori codes for the later part of the interview transcripts as participants were asked specifically about the use of Cochrane review strategies.

Textual data ~~in each transcript~~ relating to retention strategies was subsequently retrieved from a~~the~~ coded database and ~~. Coded data were~~ constantly compared within and across ~~-~~the three groups of trial personnel (i.e. PIs, TMs, RNs) ~~and within the three transcript groups~~ to ; ~~a)~~ identify the use of each strategy and ~~b)~~ to document the reported advantages and disadvantages of ~~using~~ each ~~strategy~~. Any factors thought by the interviewees to contribute to either retention or loss to follow-up were also coded and retrieved for analysis[12;13] ~~[10;1110,11]~~. ~~.~~ Relevant quotes, representing the interviewee's views, were selected to illustrate the results. Interviews were conducted between August 2010 and May 2011 until no new content or themes emerged.

Results

Fifty four trial personnel were identified for recruitment from 37 primary care trials from England and Scotland that were included in the sampling frame. ~~Ten PIs, ten TMs and nine RNs were interviewed across 23 of the 37 primary care RCTs identified for the sampling frame. tRCTs from England and Scotland were identified through the sources searched.~~ Eleven of the fifty four invitees declined to participate (PIs (n=10), TMs (n=1)). Seven of

Formatted: Not Highlight
Formatted: Font color: Auto
Formatted: Font color: Auto
Formatted: Font color: Auto
Formatted: Font color: Auto
Formatted: Do not check spelling or grammar, Superscript

For peer review only - http://bmjopen.bmj.com/site/about/guidelines.xhtml

these ~~who declined~~ recommended another ~~named~~ co-author to invite ~~for interview~~. Fourteen of the fifty four ~~invited~~ did not respond (PIs (n=10~~9~~), TMs (n=3), RNs (n=1)~~)~~. Ten PIs, ten TMs and nine RNs were recruited from 23 of the 37 primary care trials identified.

Interviewees were ~~sampled~~ from primary care trials~~RCTs~~ conducted in ~~the fields of~~ nutrition, health promotion, neurology, gynaecology, mental health, musculoskeletal, ear nose and throat (ENT), respiratory, and endocrine medicine, as well as from trials~~RCTs~~ of minor medical conditions. Collectively their experiences included trial design, implementation, coordination and data collection at GP practice sites by post, face to face and electronic methods. Trial~~RCT~~ publications reported loss to follow-up rates between 5% - 39%. Twenty interviewees were from trials~~RCTs~~ with <20% loss to follow-up reported~~.~~ (See Tables 1 and 2). Different types of follow-up were used in these trials: face-to-face follow-up at clinics (n=20), postal questionnaires (n =6), follow-up at home (n=2) or self-completion diaries (n=2). Combinations of follow-up types were also used: clinic visits or postal follow-up (n= 4), telephone and postal follow-up (n=2), register data and follow-up at home (n=1).

Formatted: Level 1

The PIs were responsible for the design and implementation of trials~~RCTs~~, including research governance and ethics approval applications. Nine PIs were GPs with academic roles, and one was a non-clinical senior academic. The TMs were based in academic research units and~~.~~ ~~They~~ managed trial data collection via telephone, post, email and SMS text message.~~;~~ TMs also~~they~~ coordinated and monitored data centrally and communicated with trial sites. Six RNs ~~were based at GP practice sites where they~~ coordinated clinical research at GP practice sites. They collected biomedical and clinical data by telephone and face-to-face interview, and ~~also~~ communicated with the coordinating centre and with PI clinicians at sites. Three RNs were based at a primary care research network coordinating centre where they answered clinical data collection queries and monitored data quality. ~~See Tables 1 and 2 for the characteristics of the study sample.~~

~~Interview~~ R~~r~~esults are presented first on the interviewee's experiences of and perspectives on using communication, incentive and new questionnaire strategies, identified by the Cochrane review, to improve primary care trial~~RCT~~ retention. This is followed by~~e~~ views on~~ -~~ other~~,~~ less frequently evaluated~~,~~ strategies identified by the Cochrane review i.e. behavioural, case management, and methodological strategies ~~are then presented~~.~~,~~ ~~followed by the results on the~~

Formatted: None

For peer review only

BMJ Open qualitative paper submitted Version 3 VB 4.11.13

F~~f~~actors thought to contribute to retention and loss to follow-up ~~-~~in primary care trials~~RCTst~~ are also presented.

The use of retention strategies identified by the Cochrane review

1. Use of i~~I~~ncentives

~~Use of~~ M~~m~~onetary incentives ~~strategies~~

Monetary incentives and offers of monetary incentives were mentioned spontaneously by most PIs and TMs as useful strategies to improve response to postal questionnaires. Over half of the PIs interviewed had used monetary incentives given in either cash or voucher format to increase questionnaire return, ~~even~~ although there was uncertainty about their effect. The different types used were~~:~~ high street vouchers and mobile phone vouchers, but vouchers were thought of as a burden to administer for trial management teams. ~~The more~~ G~~g~~eneric vouchers were thought to be more acceptable to all trial participant groups. It was felt that the incentives should be seen by trial participants as a "*thank you*" rather than as payment for participation.

The monetary value of the incentives used was between £5 and £20 and this amount was considered reasonable for adults and children ~~participants~~. Larger monetary incentives (>£50) were perceived as coercion or bribery by interviewees across the three groups interviewed. Offers of entry into a prize draw, although seldom used as a strategy to improve questionnaire response, were thought by some interviewees to be a cost effective alternative to giving a monetary incentive.

M~~The monetary incentives used in primary care RCTs were mostly given in~~ different ~~voucher formats~~ ~~rather than cash,~~. and ~~They were used to~~; a) ~~increase questionnaire return or~~r ~~at sites,~~ b) given ~~on behalf of the trial team, as a thank you to participants. The different types used were: high street vouchers and mobile phone vouchers, but vouchers were thought of as a burden to administer for trial management teams. The more generic vouchers were thought to be more acceptable to all trial participant groups.~~

There was uncertainty among TMs about ~~-~~effective ways to administer ~~-~~incentives during follow-up ~~in order~~ to optimise questionnaire response ~~for the primary outcome~~. Some ~~trial~~

For peer review only - http://bmjopen.bmj.com/site/about/guidelines.xhtml

~~managers were involved in RCTs that~~ gave a small incentive at different follow-up time points e.g. £5 at randomisation and £5 at trial completion. Others sent an~~the~~ incentive with the questionnaire, or on receipt of a completed questionnaire.

TMs and PIs sampled from trials with <20% loss to follow-up appeared to use more strategies to improve retention than those from trials with ≥20% loss to follow-up. For example those in the <20% group gave incentives at different time points to try to keep the participants motivated to return questionnaires. TMs and PIs sampled from trials with ≥20% loss to follow-up seemed more cautious about the use of incentives and some felt participants could feel coerced if incentives were used to improve follow-up.

N~~Use of n~~on-monetary incentives

Non-monetary incentive strategies or gifts i.e.~~such as;~~ pens, certificates of appreciation, key rings and mugs were sometimes used in an attempt to improve both participant recruitment and retention. These were used to market the trial, and in the case of pens, to encourage and remind participants to return completed questionnaires. Interviewees thought that these gifts could be perceived by trial participants as patronising, not useful, or associated with charity fundraising and also as a waste of public money, especially if the participant had altruistic motivations for participation~~taking part in a trial~~.

> *"I think people are very sensitive about money especially in the public sector, ….if they see something that could be considered wasteful or extravagant, I don't think it would go down too well. Because…… if you get involved in a study that's being funded from public funds and from charity, you know, it's quite an altruistic thing to do, I don't think people are looking to be rewarded for it ….then they see you wasting money on pens and mugs or, I don't think it would go down very well".*
>
> ***Trial manager interview 7***

~~Few interviewees thought that the gifts given were practical and useful to participants.~~ Some interviewees thought that the money used to finance such non-monetary / gift incentives could be better used to subsidise follow-up visits for participants. Indeed there was consensus amongst interviewees about the importance of reimbursing ~~participants'~~ travel and parking expenses associated with ~~such~~ follow-up visits. Some interviewees differentiated this from giving incentives to participants. Others recounted cases where participants did not want to be reimbursed particularly if they ~~ir~~had ~~motivation for taking part was~~ altruistic motivations for

For peer review only - http://bmjopen.bmj.com/site/about/guidelines.xhtml

participation, and others felt that ~~told of how~~ an offer of money to cover expenses could make participants feel valued.~~,~~ T~~and t~~here was a general feeling that this varied by participants' socio-economic circumstances e.g. it was thought that participants from higher socioeconomic groups were less likely to accept a monetary incentive.

> *"We actually gave them ten pounds for their travel costs, once at the beginning and once at the end.. and a lot of them didn't want it, you know, they'd say, oh... And I'd say, well just pop it in your local charity".*
>
> ***Research nurse interview 2***

Ethical approval for the use of incentives

Interviewees across all groups thought it was important that ethics committees sought justification for ~~asked questions about~~ the use of incentives in trials~~RCTs~~. They felt that ~~ethics committees still varied in their~~ opinion varied among ethics committees about the use of incentives to improve ~~response or~~ retention but o~~in RCTs. O~~verall ~~, the interviewees thought that ethics committees were~~ they were now more accepting of the use of small monetary incentives and of reimbursing participants' trial related expenses. Some PIs said that it was helpful to know who was on the ethics committee and that approval to use incentives is generally granted when a clear justification for ~~the~~ use ~~of incentives~~ is given. Others thought ~~the use of~~ incentives w~~ere~~as likely to be approved if ~~the financial incentive involved paying~~they were used to pay participant~~the~~ travel expenses ~~of the participants~~.

Formatted: Indent: Left: 0"

2. Use of communication strategies

Communication strategies, for example contact with participants by telephone, letter and email during follow-up, were spontaneously mentioned and routinely used to improve trial ~~RCT~~ retention. RNs and TMs used these as first line retention strategies.

T~~Use of t~~elephone calls ~~to participants~~

RNs frequently used telephone calls, to arrange or re schedule trial~~RCT~~ follow-up appointments or to remind participants to return postal questionnaires. Both RNs and PIs were conscious not to harass participants with too many telephone reminders. TMs thought reminder calls to participants prior to follow-up appointments might increase follow-up attendance~~-~~. A telephone reminder to a site to remind the RN that a participant is due for a follow-up appointment was also thought to potentially improve retention. PIs did not contact participants by telephone and one thought it would be "*daunting*" for a participant to receive

For peer review only - http://bmjopen.bmj.com/site/about/guidelines.xhtml

BMJ Open qualitative paper submitted Version 3 VB 4.11.13

a call from a PI. Nevertheless, PIs were involved in decisions about when to use telephone contact, especially when they thought that participants were fatigued by providing data in diaries or repeat questionnaires.

L~~Use of l~~etters ~~to trial participants~~

Letters were used predominantly by TMs to communicate with trial participants, but there was uncertainty about their effect. Electronic coloured signatures, franked institutional logos placed on outward envelopes, different coloured envelopes, and first class outward post were used to attract the attention of participants.

Letters~~, when used, ~~ were usually signed by TMs rather than PIs,~~; as~~ the TMs thought that consistency in the signatory was more important than the status of the signatory for improving ~~retention and ~~questionnaire response. Some TMs sent letters to participants in time for weekend delivery, but were unsure whether this influenced~~ed~~ response. PIs thought that letters that were personalised, and the tone of the letter were important for retention or improving questionnaire return, but that the associated large mail-outs were burdensome for trial staff. Greeting cards were used occasionally to maintain communication with participants,~~ in the hope of increasing retention.~~ h~~H~~owever~~;~~, interviewees were negative about the impact of these on retention and questionnaire response, with the exception of the use of birthday cards in paediatric trials~~RCTs~~, and~~ also~~ Christmas cards sent to sites from coordinating centres.

> *"I'm routinely suggesting that we send out birthday cards to children. We've done Christmas cards to participants on behalf of the trial team but there's no evidence that it works, but I think that it's a gesture and it's in the right direction, it's better than nothing".*
>
> ***Trial manager interview 27***

There was uncertainty ~~among the interviewees ~~about the effect on retention / response of the different types of postal delivery~~ systems~~ they use to send trial materials to participants. First class post was used to send trial materials to participants and 2nd class post for questionnaire return. While some TMs and RNs thought that 1st class post was a waste of financial resources, other TMs thought 1st class stamped letters were more likely to be opened by participants. Recorded delivery was occasionally used to send important study-related materials, or to resend questionnaires to non-responders. Some~~ interviewees~~ thought recorded

For peer review only

delivery~~this~~ was useful to find out if a participant had changed address, but others thought recorded delivery ~~was~~ could be inconvenient~~,~~ e.g. ~~for the participant~~ if the participant ~~y~~ had to collect trial supplies from a post office. ~~First class post was used to send trial materials to participants and 2nd class post for questionnaire return. While some TMs and RNs thought that 1st class post was a waste of financial resources, other TMs thought 1st class stamped letters were more likely to be opened by participants.~~

Some TMs noticed that retention improved when questionnaires were sent before clinic visits as this enabled participants to complete the questionnaire at home beforehand. A prepaid envelope for return of postal questionnaires was thought to reduce the cost of postage to participants and the inconvenience of having to buy a stamp. To minimise respondent burden, clear instructions on how to return the questionnaire once it is completed was thought by TMs to contribute to improved postal questionnaire response.

~~Use of~~ E~~e~~lectronic communication~~ontact with trial participants~~

TMs and RNs used emails to ~~-~~contact ~~with~~ participants who preferred this approach. SMS text messaging was used less so~~, but was thought to be potentially useful for retention. There were mixed views on the effect of SMS texting on retention in RCTs.~~ but m~~M~~ost interviewees thought that it would be useful for communicating with young people. The use of an automated system for texting appointment reminders similar to that used by the NHS for clinic appointments was thought to be a potentially useful way to improve trial retention. However one RN reported that the reminders sent via the system did not improve clinic attendance.

N~~Use of n~~ewsletters ~~in RCTs~~

Most interviewees thought newsletters were useful for keeping participants and site clinicians informed about trial recruitment, retention, and general trial news ~~about the trial~~. Others thought these~~y~~ were less useful and could bias trial results by contaminating and confounding the results of the *treatment as usual* group. The frequency with which newsletters were sent to participants~~,~~ varied from fortnightly to annually, and depended on the condition /~~or~~ disease, intervention, and length of follow-up. ~~TMs and RNs were less convinced of their usefulnessto participants. However~~ S~~s~~ome PIs thought~~mentioned that~~ having trial-specific information and answers to frequently asked questions as a resource on a website could benefit retention.

For peer review only - http://bmjopen.bmj.com/site/about/guidelines.xhtml

BMJ Open qualitative paper submitted Version 3 VB 4.11.13

The TMs and PIs from RCTs with loss to follow-up of <20% appeared to have usedmore strategies , to reduce loss to follow-up compared with TMs and PIs from RCTs with ≥20% loss to follow-up. For example they spoke of giving incentives at different time points in a trial to try to keep the participants motivated. TMs in this group also used the telephone to contact participants. TMs and PIs ofRCTs with ≥20% loss to follow-up e appeared to be more cautious about the use of incentives because they felt participants could be being coerced.

3. Use of new questionnaire formats strategies

None of the interviewees spontaneously mentioned using modifyingications ato follow-up questionnaires to improve response or retention, although some PIs and TMs did use had used shorter questionnaires for non-responders. When asked specifically about the impact effect of questionnaire length on retention, interviewees expressed a preference for shorter questionnaires, which they were thought to increased response. Interviewees also thought that long questionnaires administered frequently led to "*questionnaire fatigue*", and some thought that dividing long questionnaires into several short questionnaires could be useful to reduce the burden of data collection on participants. However, some PIs thought that if the questionnaire content was of interest to the participants, then the length of the questionnaire may not affect response. TMs and PIs thought the involvement of mentioned that involving patient representatives in the design and pre-test of questionnaires could improve retention. Many suggestions were also offered for improving questionnaire design in order to increase questionnaire response (Figure 1).

How athe questionnaire was administered and by whom was also thought to to also affect impact on questionnaire response and retentionretention. Some PIs and TMs thought that if data were collected at the questionnaire is administered at the the clinic site by a nurse rather thann sent by post that this could increase response.:

4. Use of other strategies to improve retention

Other strategies identified by the Cochrane –review were not routinely used or mentioned spontaneously by interviewees as ways to improve retention. Opinions were mixed about the usefulness of These were: a) a behavioural strategy where participants are given information about goal setting and time management to facilitate successful trial completion; b) methodological strategies where a blind trial design was compared to an open / unblind trial

Formatted: Not Highlight
Formatted
Formatted: Not Highlight
Formatted
Formatted: Not Highlight
Formatted
Formatted: Not Highlight
Formatted
Formatted: Not Highlight
Formatted
Formatted: Not Highlight
Formatted
Formatted: Not Highlight
Formatted
Formatted: Not Highlight
Formatted
Formatted: Not Highlight
Formatted
Formatted: Not Highlight
Formatted
Formatted: Not Highlight
Formatted
Formatted: Not Highlight

For peer review only

design; c) case management where trial assistants manage participant follow-up, by arranging services to enable participants to keep trial follow-up appointments. ~~Opinions were mixed about the usefulness of such strategies as ways to improve retention in primary care RCTs.~~

Behavioural strategies were described to interviewees as *"Arranging workshops to give participants information about goal setting and time management"* for example in the context of~~–~~ an exercise behaviour change intervention~~parenting and exercise RCTs~~. When asked specifically about the use of such a ~~these~~ strateg*y*~~ies~~, interviewees were generally negative. Such strategies were discussed in the following ways: "*strange*", "*preachy"*, "*condescending*", "*never came across it", "would never think of doing it*", *I'd be surprised if it worked*" and "*would need evidence that it worked*". Some thought that a behaviour~~behaviourral~~ change retention strategy could~~strategies should~~ not be used ~~only be used as part of a trial intervention rather than as strategies to improve retention, in RCTs and they felt that type of~~ because it ~~-~~may ~~strategy would~~ contaminate a behaviour change trial ~~the~~ intervention and ~~and could~~therefore may affect the generalisability of trial results.

Although the interviewees were familiar with the concept of blind and open trials~~RCTs~~, they were not aware of the use of either design as a strategy to improve trial retention. Interviewees felt that using an open trial design to improve retention could bias~~trial~~ results, particularly as blinding is used to reduce bias in trials ~~and also that participants may drop out of a RCT if they are not allocated to their preferred intervention~~. They also felt that ~~some interventions, for example therapist-led behavioural interventions, cannot be evaluated using~~a blind trial design~~RCT designs~~ could not be applied to therapist-led behavioural interventions~~.~~

One TM had used case management to improve trial retention. This involved the TM arranging services to enable participants to attend trial follow-up appointments. The interviewees generally thought that case management could improve trial retention with some groups e.g. the elderly, disabled and participants with low incomes but they also thought this could create dependence on the service. The impact of this strategy on retention was thought by some to depend on the personality of the case manager.

F~~Other f~~actors thought to contribute to trial retention ~~in primary care RCTs~~

P~~Use of p~~articipant's time

Formatted
Formatted: Not Highlight
Formatted
Formatted: Not Highlight
Formatted
Formatted: Not Highlight
Formatted
Formatted
Formatted: Not Highlight
Formatted
Formatted: Not Highlight
Formatted
Formatted
Formatted: Not Highlight
Formatted
Formatted

For peer review only - http://bmjopen.bmj.com/site/about/guidelines.xhtml

BMJ Open qualitative paper submitted Version 3 VB 4.11.13

A key factor thought to contribute to retention was reducing the burden of follow-up for participants in terms of the length of time they spend in follow-up activities, and ~~also~~ the type of data collected (e.g. biomedical specimens, long questionnaires). ~~,~~ ~~by using~~ M~~m~~ore convenient ways to collect outcome data, e.g. ~~such as~~ online data collection~~bases~~ were thought to be more effective for retention. ~~.~~

> *"The burden of activity on the patients, how easy it is for them, how long it's going to take, how intrusive in terms of blood samples or answering huge long questionnaires, it's partly the complexity as well, if it's very simple for them to do, often you just want very simple data back from people, alive or dead, or specific complications, if it can be done either with a, click of a button by sending a text or an email to people or just …~~a very you know,~~ reply paid cards so you're just getting the data back from them, ..~~I suppose~~ the other thing is…~~.~~…~~trying~~ to do electronic follow-up as much as possible so you don't actually have to bother the patient, you have their consent to access their electronic records and either their Primary care data can be interrogated …~~a way of linking all of those together, like with Cancer Registry, Registrar General data, so I suppose the more you can automate it the easier it becomes~~".*
>
> ***Principal investigator interview 4***

The time participants spend getting to, ~~and~~ from, and at follow-up visits, giving participants adequate time at follow-up visits, and ensuring that participants were not kept waiting too long for follow-up appointments were all considered important factors for retention.

> *"The cost to the patient in time and effort to get to the surgery,…what time these appointments would be. How long they would take, how much effort it would take to get the patient… and also, if it's a research project, I always feel that patients shouldn't wait, …it's up to the nurse to make her appointments work so that she doesn't have anybody waiting there for any length of time,*
>
> ***Research nurse interview no 1***

Engaging with participants

The recruitment visit was seen as an ~~very~~ important opportunity~~time~~ to~~for~~ engag~~e~~~~ing~~ with participants and to giv~~e~~~~ing them~~ key~~important~~ information about trial follow-up procedures.

> *"It [the recruitment visit] sets the stage for the participants relationship with the trial …………. it's important to get that right, right from the start…. that affects whether people think it's worthwhile coming back…"*
>
> ***Trial manager interview 26***

For peer review only - http://bmjopen.bmj.com/site/about/guidelines.xhtml

BMJ Open qualitative paper submitted Version 3 VB 4.11.13

Establishing a rapport with participants that continues throughout follow-up was also thought to contribute ~~-~~to retention.

> *"At the end of the day it's how the nurse and GP …….establish a rapport and a relationship with the participant,… that is the key issue in keeping participants in a study".*
>
> ***Research nurse interview 12***

Good rapport was thought to be maintained through continuity of trial staff at~~ the~~ trial sites for follow-up visits. Some TMs thought that having "*personal*" or "*good*" communication with participants and within the research team ~~itself~~ contributed to participants returning to sites for trial follow-up~~ visits~~ .

> *"I think it is communication at all levels; so the nurse who interfaces with the trial management team behind the coordinating centre, … listening to the participant's views …and … feeding them back, ……. a two-way process. Because we're this side doing the research, we have to listen … so yes, … communication and understanding [the] population that you're working with".*
>
> ***Trial manager interview 13***

TMs use combinations of different communication ~~have~~ strategies ~~in place~~ to increase response to postal questionnaires ~~using different communication methods to engage with trial participants from the coordinating centre~~ e.g. combining postal and telephone methods for non-responders.~~.~~

> "*We …..mak(e) sure that we have [communication] strategies in place to get as many …..responses to our postal questionnaires …we send out the questionnaire…, if we don't hear back ….we will ring up to say ….''Could you possibly send it back to us?'' If we still don't hear …we'll send .. another copy … if we don't have a response from that copy then we'll ring them again…if we still don't hear we've telephoned …again to collect a set of core outcomes to get a response………"*
>
> ***Trial manager interview 14***

Some interviewees across all groups mentioned the impact of trial staff~~the~~ personalities ~~of trial staff~~ on trial retention. Interviewees thought~~ trial~~ RNs needed to have a "*flexible approach*" and to "~~-~~*be sympathetic*" toward participants, while TMs needed to be "*competent*", "*personable*", "*persistent*", "*enthusiastic*", and "*good communicators*".

For peer review only

"If you're enthusiastic, can give the information to the participants and…[are] sympathetic to …. queries, always being accommodating,[and] getting back to them if they have any queries …being absolutely spot on with, so that they don't feel they've been let down or you haven't been bothered to get back to them."

Research nurse interview 5

"I think there's a paradox here because they [trial managers] need to be obsessional about detail but they also need to be relaxed and flexible in their response to different situations ….and problems that come up. That's asking quite a lot, I think. So you know it's great if you find somebody who's good at it…..Or be very kind hearted. And prepared to work out of hours!"

Principal investigator interview 25

Perceived benefit of trial participation ~~to the participant~~

Most interviewees thought that if trial participants perceived an extra benefit ~~o~~from participati~~o~~ng in a trial that this would contribute to retention ~~in the trial~~ particularly if they are being monitored for disease progression.

"In studies where (there) is … constant monitoring ....they don't mind giving….. samples again and again as part of the trial….. But I …think …. if they can see some benefit then they'll do it but if they can't or if they've already got that benefit from giving the sample at the first time point they might not want to do it again and again"

Trial manager interview 10

Some of the benefits identified from participation were extra health monitoring for example: *"ECG" "BP"* and *"cholesterol"* ~~and other *"health checks"*~~ as well as ~~having~~ the extra attention from staff. However, these perceived benefits were thought to be trial dependent.

Participant altruism

Participant~~participant~~ altruism was also thought to improve retention ~~,~~ where trial participants~~people~~ were committed to a trial for the better good of society:

"…They [participants] feel as though they want to do good because they often, a lot of them say "oh it's payback time", they think the NHS has been good to them so they want to give something back".

Trial manager interview 7

Tailoring retention strategies

Tailoring ~~tailoring~~ specific retention ~~trial retention~~ strategies to enable different trial population groups to either return questionnaires or return to sites for follow-up was thought

Formatted: Line spacing: single

For peer review only

For peer review only - http://bmjopen.bmj.com/site/about/guidelines.xhtml

BMJ Open qualitative paper submitted Version 3 VB 4.11.13

to improve retention. ~~For example I~~In mental health trials for example,~~RCTs,~~ some interviewees ~~had~~ found that ~~some~~ participants may wish to be followed-up~~seen~~ at home rather than at ~~than returning to~~ a clinical site~~s for follow-up visits~~.

Formatted: Not Highlight

Formatted: Not Highlight

Formatted: Not Highlight

"Depending on the trial and the length of the trial and your client group …you would have a completely different strategy for… the fifteen to twenty-four year olds, to something… where you're looking at the seventy-five plus….. you do sort of tailor it [the strategy] to your group."

Research Nurse interview 2

Factors thought to contribute to loss to follow-up ~~in primary care RCTs (Box 2)~~

~~Participant age was thought to affect retention in primary care RCTs.~~ Several age groups were highlighted as challenging for trial retention, for example, teenagers wishing to opt~~ing~~ out of a trial when they reach the age of consent; working mothers juggling school runs with work time~~ and follow-up visits~~,~~;~~ and elderly participants who have either lost~~ing~~ their independence or are ~~being~~ involved with extended family caring activities.~~,~~ Y~~and y~~oung men with other interests were also thought to be challenging to retain in trials~~.~~

"A young girl whose parents had agreed to be in the trial and then, you know, she didn't want to and then decided herself she was going to withdraw. And, you know, I can see the potential for that sort of thing if it's a trial in the sort of pre-teen, teen group that,... they might well change their mind….more interesting things going on in their lives, they've got more after school activities or they've got exams or they've got whatever. And coupled with being more independent and wanting to assert their own ideas".

Principal investigator interview 28

"In trials with older people, people will die, people will get frailer and maybe be institutionalised and not be able to continue or not want to continue for those sort of reasons".

Principal investigator interview 28

"There are all the usual things, we assume that older people don't move, there's a astonishingly high level of mobility in the older population. So lost to follow-up can occur just because people say "right, I'm going to go and live in…………..." or "I'm going to go and live near my daughter" or "I've off to ……….for the winter". [They] do… a lot of child care, grandparental child care and sorting out their children's

For peer review only

For peer review only - http://bmjopen.bmj.com/site/about/guidelines.xhtml

divorces and so on and so they get distracted and drop out completely through distraction".

Principal investigator interview 21

~~Trial participants' feelings of guilt and shame with not having achieved positive outcomes in a trial were thought to contribute to loss to follow-up in some disease areas / conditions.~~ Some interviewees thought that if participants did not perceive a benefit from participation they might not return for their follow-up visit. Feelings of guilt and shame with not having achieved positive outcomes in a trial were thought to contribute to loss to follow-up in some disease areas / conditions~~They thought for example~~ e.g. ~~that~~ in treatment for obesity trials~~RCTs~~, if participants were not losing weight they might not return to be weighed perhaps due to the shame and stigma associated with not achieving their expected aims. Retention in trials~~RCTs~~ involving behaviour change was also thought to be problematic if change targets were not achieved by participants. For example, interviewees were generally in agreement that retention can be problematic in trials~~RCTs~~ involving participants with a mental health issue.

"In nutrition studies we're very much bound by looking at healthy individuals to begin with. So from that perspective they may certainly be less motivated than an individual participating in a drug trial who has a severe or debilitating condition that, you know, they want to try and do something about".

Trial manager interview 15

"For smoking cessation or weight management or any of these things where certain behaviour is expected or wanting to be demonstrated. If they're not achieving it they are more likely to just drop out"

Research nurse interview 9

"Bi-polar groups, a lot of them find it very, very difficult going to an NHS facility".

Trial manager interview 17

"it depends on what area of medicine it is, for instance, if you're dealing with people with psychiatric illness, there's always a problem with attendance, whether that's within the surgery or whether that's within research".

Research nurse interview 1

The i~~I~~nterviewees ~~also~~ felt that participants with chronic diseases may have more interest in the treatment of their condition and return for follow-up visits ~~which would contribute to~~

For peer review only - http://bmjopen.bmj.com/site/about/guidelines.xhtml

~~retention~~. Others thought healthy volunteers may drop out of trials~~RCTs~~ because of lack of interest in the trial.

Some interviewees reported that the working environment at general practice trial~~RCT~~ sites might impact upon ~~-~~retention. ~~and that~~ I~~i~~t was thought to be important to have a staff member at the site to "*champion*" the study ~~locally~~. Part-time RNs reported working in isolation and~~, and~~ sometimes struggled to find a consulting room for follow-up visits which~~,~~ restrict~~ed~~ing the availability of flexible follow-up appointment times to offer to participants.

> *"The last study that I worked on, ..I struggled to find .. a room, the practice were incredibly accommodating, and really wanted me to have a room, but it just is very, very tight. ...it has been, a major issue for me…It was affecting follow up for me because I was not always able to have a room when I was available, that ….would have been potentially available for participants, so it just narrowed down the opportunities for me to offer to participants to come in"*
>
> ***Research nurse interview 5***

> "*if somebody genuinely did want to make the trial work then it was possible to make it work. ………………..it needed somebody to actually manage the trial within that practice. The practices in which we had success I felt that we had a champion there who was fighting our corner for us"*
>
> ***Trial manager interview 27***

> "*the other thing is practice awareness, you know, because you said it's a big practice and if somebody comes in and hasn't got the sort of clock-in bit straight and they go to the desk and say, I've come to see X about this trial and, you know, new receptionist knows nothing. That can be quite off-putting so as long as everybody's engaged …and everybody knows about it".*
>
> ***Research nurse interview 2***

Discussion

Statement of principal findings

Based on in-depth interviews with primary care trial~~RCT~~ personnel we identified that incentive, communication and questionnaire strategies are used to try to increase retention in primary care trials~~RCTs~~. Making efficient use of participant time and appropriate engagement~~ing~~ with participants~~them~~ were also thought to ~~promote~~ increase retention ~~as was the~~

For peer review only - http://bmjopen.bmj.com/site/about/guidelines.xhtml

~~perception by participants that they would have some benefit from participation~~. Interviewees ~~also~~ thought that participant's perceptions of benefits from participation; the age of participants e.g. young men and teenagers; the disease area / condition e.g. mental illness and behavioural problems; and the research environment in which the trial was conducted e.g. availability of consulting rooms for follow-up appointments ~~were~~ all contribut~~ed~~ting ~~factors~~ to loss to follow-up in trials~~RCTs~~.

Strengths and weaknesses

~~There is limited work on~~Research on strategies to improve retention of participants ~~retention~~ in ~~the retention of participating individuals in the area of~~ primary care trials~~RCTs. Work to date~~ is limited and commonly grouped with recruitment strategies ~~for participants and trial sites~~,[14;15] ~~[12;1312,13]~~. Trial retention is important because loss to follow-up can lead to incomplete data for the primary outcome, bias results and impact on the generalisability of trial findings. This study is the first to explore the use of retention strategies in UK primary care trials~~RCTs~~ and the factors associated with retention ~~and loss to follow-up~~. Interviews were conducted with researchers experienced in ~~the~~ design, leadership, management, and implementation ~~at site level~~ of UK primary care trials, many of whom reported that~~RCTs most of whom had used strategies to improve retention.~~ ~~P~~prior to this study ~~some of the interviewees reported~~ that they had not thought deeply about loss to follow-up or ~~the~~ strategies used to overcome this. With the focus by funders on recruitment rather than retention targets, this study specifically raises awareness of the complexity of retention in primary care trials~~RCTs among primary care researchers~~ and the many strategies and factors used to maximise retention. Although the thoughts and experiences of trial participants were not captured to further ~~A limitation of this study is that the thoughts and experiences of trial participants who have dropped out or of those who were retained in RCTs were not captured to further~~ explore the barriers and facilitators to retention ~~in primary care RCTs~~ the results show that trial personnel have an understanding of the challenges participants encounter to stay in trial follow-up. I~~Also i~~t is un~~not~~ clear how generalisable the results are to trials~~RCTs in other areas~~ outside of ~~those conducted in the context of~~ primary care.

Meaning and implications for the use of retention strategies

These results enhance the interpretation and implementation of the results of our~~the~~ Cochrane review on ways to improve retention in trials,[2] ~~RCTs[21]~~. T~~h~~ogether, these two studies~~, and used~~

Formatted: Do not check spelling or grammar, Superscript

Formatted: Do not check spelling or grammar, Superscript

For peer review only - http://bmjopen.bmj.com/site/about/guidelines.xhtml

BMJ Open qualitative paper submitted Version 3 VB 4.11.13

together, can inform the future use of effective strategies to improve retention in primary care trialsRCTs.

There was general agreement across the groups that small monetary incentives were likely to be viewed favourably by ethics committees acceptable in the context ofand led to increaseding questionnaire response, in agreememntagreement with the findings of the Cochrane Review[2]. , and were likely to be viewed favourably by ethics committees, aAlthough there was uncertainty among those interviewed about whether monetary incentives should be given up front or offered on receipt of data, . Tthe Cochrane review shows showed that both either approach increased postal questionnaire response[2221].

Field Code Changed

Formatted: Do not check spelling or grammar, Superscript

Formatted: Do not check spelling or grammar, Superscript

Scepticism among interviewees about Nnon-monetary incentives are also used in primary care trialsRCTs, but with considerable scepticism as toand their value to the extent that it was thought that these could have a negative impact on trial retention and questionnaire response. wWas also supported by the Cochrane Review, which did As this type of incentive was not found find them to be effective in the Cochrane review. , primary care trialists or indeed any Ttrialists may therefore wish need to reconsider the use of this type of incentive in trialsRCTs.

A range of different Ccommunication strategies were discussed by interviewees. Although trialists routinely appeapreferred red to be commonly used to increase return to sites for follow up and to improve return of questionnaires. First1st class post, and the use of enhanced letters, the Cochrane Review found no evidence to support either approacht. is used routinely for outgoing post. Based on two studies, Edwards systematic review found that 1st class post was effective at increasing questionnaire response but the result iswas not specific to trialsRCTs. THowever the Cochrane review of retention strategies in trialsRCTs[2] demonstrates no benefit of using 1st class post for outgoing mail[1414]. Therefore, 2nd class post and standard letters can probably be used for postal correspondence with primary care trial participants, with little impact oin loss to follow-up. Also, some trial managers did post letters to trial participants so that they would arrive on a Saturday.

Formatted: Do not check spelling or grammar, Superscript

Formatted: Do not check spelling or grammar, Superscript

Although Trialists had mixed opinions on the use of recorded delivery, was used for important study related materials and to resend questionnaires, this was thought to be potentially frustrating for participants if delivery was made while they were out.

For peer review only

BMJ Open qualitative paper submitted Version 3 VB 4.11.13

nNevertheless recorded delivery of questionnaires compared with a telephone reminder was found to be effective for improving questionnaire response[2;5] .[2;51,5]. To avoid any inconvenience associated with the use of this strategy, preplanning the delivery time with the participant with a reminders and reminding them to expect a recorded delivery of trial materials is recommended if this strategy is to be used in future trialsRCTs. The trial managers describe enhancing the letters they send. However, the Cochrane review showed no effect for using such enhanced letters[2], although the TDM, a postal communication package that encompasses sending hand prepared letters with a series of reminders to improve questionnaire response, was found to be effective based on the results of one trial reported in 1996[15]. TDM may not be applicable to all participant groups (e.g. young people) or for trials[21]. Although the TDM, a communication package that encompasses sending hand prepared letters with a series of reminders, to improve questionnaire response, was found to be effective, based on the results of one trial reported in 1996[15]. This approach may not be applicable to all participant groups (e.g. young people), or for RCTs using electronic data collection methods.

Different types of reminders and alternative questionnaires are routinely used by primary care researchers to improve trial follow-up, hHowever, there is no but our Cochrane review found no clear evidence that reminders and prompts in addition to the standard trial follow-up procedures are beneficial to retention[2]. Also, Edwards found that telephone reminders did notor to improveing questionnaire response. Similarly, there is no evidence tto suggest better response rates to shorter questionnaires [2]. Different types of reminders are routinely used by primary care researchers to improve trial follow-up, but the Cochrane review found no clear evidence that reminders and prompts in addition to the standard trial follow-up procedures are beneficial to retention. Also, Edwards found that telephone reminders did not improve questionnaire response[14]. Reminders are part of the standard trial follow-up procedures in RCTs for collection of data from non-responders and. They can play an important part in participant engagement especially if there is little face to face contact with trial participants. However, the interviewees in this study thought too many additional reminders could be perceived as harassment by trial participants and lead to loss to follow-up.

Several alternative questionnaire formats were thought to be potentially useful for increasing response, but not are used as first-line strategies. Shorter questionnaires were used by some

Formatted: Do not check spelling or grammar, Superscript

Formatted: Do not check spelling or grammar, Superscript

Formatted: Do not check spelling or grammar, Superscript

Comment [FS1]: Do you need retention strategies for this type of research?

To improve response to electronic questionnaires?

Formatted: Do not check spelling or grammar, Superscript

Field Code Changed

For peer review only - http://bmjopen.bmj.com/site/about/guidelines.xhtml

BMJ Open qualitative paper submitted Version 3 VB 4.11.13

~~interviewees to increase questionnaire response. Although Edwards found that shorter questionnaires increased questionnaire response in research generally[14], this was not borne out in trials by our Cochrane review[2]. A strategy also mentioned but not yet evaluated is to divide long questionnaires into several short questionnaires to reduce the burden for participants of data collection associated with longer questionnaires. This may not be practical in trials[14], this was not borne out by the Cochrane review in RCTs [21]. A strategy also mentioned but not yet evaluated is to divide long questionnaires into several short questionnaires to reduce the burden for participants of data collection associated with longer questionnaires. This may not be practical in RCTs where several there are a number of follow-up questionnaires are to be completed.~~

~~Behavioural and methodological strategies were not used in the context of these UK primary care RCTs.~~ Furthermore, ~~The~~ interviewees were particularly negative toward the use of behavioural strategies and there was no effect found for these in our~~the~~ Cochrane review[2] [21]. Case management, although seldom used, was thought to be potentially useful by interviewees, however, there is no evidence that this is effective[2]. The interviewees also thought that blinding participants to their allocated intervention would improve retention. This is in contrast to findings from the~~, and . However, our~~ Cochrane review which found that an open trial design was more effective than a blind design in ~~the context of secondary osteopathic fracture~~ one trial~~prevention~~[2;4] [16]. Avenell argues that double blind trials~~RCTs~~ do not reflect usual health care procedures, and that open trials~~RCTs~~, where the intervention is compared to no treatment or usual treatment, could give a better measure of the differential effects in normal care settings[4][16]. The open trial design, if used as a stand-alone strategy to improve retention, would need to be considered in the context of the overall aims and objectives of the trial. Further evaluation ~~of this strategy~~ is needed in different trial contexts and settings if this retention strategy~~it~~ is to be adopted~~ as a strategy to improve trial retention~~. ~~Although case management was seldom used, elements of this strategy were thought to be useful but there is no evidence that thisit is effective[2] [21].~~

Several other factors and ways to~~aimed at~~ improve~~ing~~ retention were identified that were not covered in our Cochrane review[2]. Some of these~~ strategies~~ such as~~ the~~ relationships and communication between trial staff and trial participants are clearly more difficult to design interventions for and to evaluate. The factors identified indicate a need to train and support trial staff in ways to improve trial retention. It seems clear from this qualitative study that trial

Formatted: Do not check spelling or grammar, Superscript

Formatted: Do not check spelling or grammar, Superscript

Formatted: Do not check spelling or grammar, Superscript

Field Code Changed

Formatted: Do not check spelling or grammar, Superscript

Formatted: Do not check spelling or grammar, Superscript

Formatted: Do not check spelling or grammar, Superscript

Formatted: Do not check spelling or grammar, Superscript

For peer review only - http://bmjopen.bmj.com/site/about/guidelines.xhtml

staff~~ ~~ were oriented to patient concerns and needs (as they perceived them) and had a high level of empathy with participants.

Integrating qualitative research with Cochrane reviews

Integrating the results of the qualitative study and the Cochrane review helped us understand the different experiences ~~of~~ primary care researchers have with the ~~in the use of the retention~~ strategies identified by our Cochrane review[2] ~~-~~. The qualitative results support some of the results of our~~the~~ Cochrane review and demonstrate the potential transferability of the different effective retention strategies to trials~~RCTs~~ conducted in primary care. For example incentives were employed in different~~in different~~ ways to try to improve responses to postal questionnaires ~~used in primary care RCTs~~. The results have also helped identify other factors that might promote retention such as building a rapport with trial participants, and potential barriers like finding a consulting room for follow-up appointments. This mixed methods approach could be replicated in ~~other~~ research contexts apart from primary care to determine how appropriate and useful the strategies identified by the Cochrane review are in practice.

While the absolute effects of effective strategies identified by the Cochrane review are modest[2] ~~.~~ this qualitative study highlights that multiple strategies are often used to try to achieve maximum retention in primary care trials~~RCTs~~. Furthermore~~Also~~ it suggests that clinical trialists~~primary care researchers~~ should consider other factors that may affect retention, such as building a rapport with participants and minimising the impact of trial follow-up on their daily lives. Consideration should also be given to the characteristics of the intervention, participants and trial personnel when choosing effective strategies identified by our ~~the~~ Cochrane review ~~are being considered~~ to improve trial retention[2].

Future research

It was clear from the interviews that there is a move toward contacting participants by email and a desire to use text reminders when automated facilities for these become available for use in randomised trials~~RCTs~~. These new strategies would need evaluation in future trials~~RCTs~~.

Further research is also needed to identify potential barriers and facilitators to follow-up in trials~~RCTs~~ e.g. with trial participants from different social, economic, age and disease groups. The results would be useful for primary care researchers and would help target and tailor

Formatted: Justified

Formatted: Do not check spelling or grammar, Superscript

Formatted: Do not check spelling or grammar, Superscript

Formatted: Do not check spelling or grammar, Superscript

For peer review only - http://bmjopen.bmj.com/site/about/guidelines.xhtml

strategies to meet the needs of different population groups to keep them specifically engaged in trial follow-up[16 17]. Incorporating the views of trial participants, including those who have withdrawn, in future research is challenging but would enhance understanding what works best and why.

Formatted: Do not check spelling or grammar, Superscript

Conclusion

Qualitative research can help to interpret the results of systematic reviews by telling us what researchers do or think happens in research practice. These findings provide a deeper understanding of the factors that may facilitate retention such as rapport with participants and respect for their time, and highlights the participant and environmental characteristics that can limit the implementation of effective strategies. The use of small monetary incentives to improve questionnaire response is effective and acceptable however a. A reconsideration of the use of non-monetary incentives, and certain communication strategies including 1st class post is needed. A similar exploration of barriers and facilitators to retention in other research contexts would help identify ways to improve retention in trialsRCTs conducted in other research contexts.

For peer review only - http://bmjopen.bmj.com/site/about/guidelines.xhtml

BMJ Open qualitative paper submitted Version 3 VB 4.11.13

Article Summary

Article focus

High levels of retention in trials~~RCTs~~ are important ~~in order~~ to maintain power and minimise bias. Strategies to improve retention are widely used in trials~~RCTs~~ but without evidence of effect.

Strategies thought to improve retention in UK primary care randomised ~~controlled~~ trials ~~(RCTs)~~ have not been well documented or evaluated. This study explores the use, in UK primary care trials~~RCTs~~, of strategies to improve retention.~~, identified by a Cochrane review. It also explored factors associated with retention and attrition.~~

Key messages

Small monetary incentives, non-monetary incentives and communication strategies are used in primary care trials~~RCTs~~ to improve retention. Behavioural, methodology and case management strategies were not used. Small monetary incentives and communication strategies were thought to be useful for retention and case management thought to be potentially useful. Interviewees were negative about the use of behavioural, methodology and non-monetary incentive strategies.

Factors thought to contribute to retention are: effective communication, participant altruism and perceived benefits of participation for participants, burden reduction, personality and flexibility of trial staff.

Factors thought to contribute to loss to follow-up are: participant age, disease/condition and research environment.

Strengths and limitations

This study is the first to explore the use of retention strategies in UK primary care trials and~~RCTsand~~ the factors associated with retention and loss to follow-up. Interviews were conducted with researchers experienced in UK primary care trials~~RCTs~~, many of whom had used strategies to improve retention.

A limitation is that the thoughts and experiences of trial participants have not been gathered to further explore the barriers and facilitators to retention in primary care trials~~RCTs~~.

For peer review only

For peer review only - http://bmjopen.bmj.com/site/about/guidelines.xhtml

Acknowledgements

We would like to thank all twenty nine interviewees who gave of their time and thoughts on the complexities of trial retention in primary care.

Statement of funding and competing interests

All authors have completed the ICMJE uniform disclosure form and declare no competing interests. This project was funded by the Medical Research Council Population Health Sciences Research Network grant number PHSRN 30

For peer review only - http://bmjopen.bmj.com/site/about/guidelines.xhtml

Reference List

(1) Janson SL, Alioto ME, Boushey HA. Attrition and Retention of Ethnically Diverse Subjects in a Multicenter Randomized Controlled Research Trial. *Control Clin Trials* 2001; 22(6, Supplement 1):S236-S243.

(2) Brueton VC, Tierney J, Stenning S, Nazareth I, Meredith S, Harding S et al. Strategies to improve retention in randomised trials . *Cochrane Methodology Group* 2013; in press.

(3) Sutherland HJ, Beaton M, Mazer R, Kriukov V, Boyd NF. A randomized trial of the total design method for the postal follow-up of women in a cancer prevention trial. *Eur J Cancer Prev* 1996; 5(3):165-168.

(4) Avenell A, Grant AM, McGee M, McPherson G, Campbell MK, McGee MA et al. The effects of an open design on trial participant recruitment, compliance and retention - a randomized controlled trial comparison with a blinded, placebo-controlled design. *Clinical Trials* 2004; 1(6):490-498.

(5) Tai SS, Nazareth I, Haines A, Jowett C. A randomized trial of the impact of telephone and recorded delivery reminders on the response rate to research questionnaires. *J Public Health* 1997; 19(2):219-221.

(6) McColl EM, Eccles MPM, Rousseau NSB, Steen INP, Parkin DWD, Grimshaw JMP. From the Generic to the Condition-specific?: Instrument Order Effects in Quality of Life Assessment. [Article]. *Med Care* 2003; 41(7):777-790.

(7) Cockayne S, Torgerson D. A randomised controlled trial to assess the effectiveness of offering study results as an incentive to increase response rates to postal questionnaires [ISRCTN26118436]. *BMC Medical Research Methodology* 2005; 5(1):34.

(8) Man MS, Tilbrook HE, Jayakody S, Hewitt CE, Cox H, Cross B et al. Electronic reminders did not improve postal questionnaire response rates or response times: a randomized controlled trial. *J Clin Epidemiol* 2011; 64(9):1001-1004.

(9) Dangour AD, Allen E, Elbourne D, Fasey N, Fletcher AE, Hardy P et al. Effect of 2-y n-3 long-chain polyunsaturated fatty acid supplementation on cognitive function in older people: a randomized, double-blind, controlled trial. *Am J Clin Nutr* 2010; 91(6):1725-1732.

(10) Williamson I, Benge S, Barton S, Petrou S, Letley L, Fasey N et al. Topical intranasal corticosteroids in 4-11 year old children with persistent bilateral otitis media with effusion in primary care: double blind randomised placebo controlled trial. *BMJ* 2009; 339(dec16_1):b4984.

(11) Trial Managers Network. 2013.
Ref Type: Online Source

Formatted: Space After: 12 pt

Formatted: Font: Italic, Do not check spelling or grammar

Formatted: Font: Italic, Do not check spelling or grammar

Formatted: Font: Italic, Do not check spelling or grammar

Formatted: Font: Italic, Do not check spelling or grammar

Formatted: Font: Italic, Do not check spelling or grammar

Formatted: Font: Italic, Do not check spelling or grammar

Formatted: Font: Italic, Do not check spelling or grammar

Formatted: Font: Italic, Do not check spelling or grammar

Formatted: Font: Italic, Do not check spelling or grammar

Formatted: Font: Italic, Do not check spelling or grammar

Formatted: Space After: 12 pt

For peer review only

Qualitative retention paper BMJ submission 18.06.13

(12) Pope C, Ziebland S, May N. Analysing qualitative data. In: Catherine Pope, Nicolas May, editors. Qualitative Research in Health Care. second ed. London: BMJ Publishing Group; 2000. 75-88.

(13) Farmer T, Robinson K, Elliott SJ, Eyles J. Developing and Implementing a Triangulation Protocol for Qualitative Health Research. *Qual Health Res* 2006; 16(3):377-394.

(14) Leathem C, Cupples M, Byrne M, O'Malley M, Houlihan A, Murphy A et al. Identifying strategies to maximise recruitment and retention of practices and patients in a multicentre randomised controlled trial of an intervention to optimise secondary prevention for coronary heart disease in primary care. *BMC Medical Research Methodology* 2009; 9(1):40.

(15) Graffy J, Grant J, Boase S, Ward E, Wallace P, Miller J et al. UK research staff perspectives on improving recruitment and retention to primary care research; nominal group exercise. *Fam Pract* 2009; 26(1):48-55.

(16) Kimmel SE, Troxel AB. Novel incentive-based approaches to adherence. *Clin Trials* 2012; 9(6):689-695.

Formatted: Font: Italic, Do not check spelling or grammar

Formatted: Font: Italic, Do not check spelling or grammar

Formatted: Font: Italic, Do not check spelling or grammar

Formatted: Font: Italic, Do not check spelling or grammar

Formatted: Indent: Left: 0", Hanging: 0.5", Tab stops: 0.38", Right + 0.5", Left

Formatted: Normal

For peer review only - http://bmjopen.bmj.com/site/about/guidelines.xhtml

Table 1 Characteristics of interviewees

**Characteristics**	**Number of interviewees**
Male	10
Female	19
**Role**	
Research nurse	9
Principal investigator	10
Trial manager	10
**Location**	
London	8
Midlands	8
Northeast England	3
Southwest England	3
East of England	5
Scotland	2
**Unit**	
University/Research organisation based	23
GP practice site based	6

Table 2 Other sample characteristics

**Other characteristics**	**Number of interviewees**
No of interviewees from trials published between 2000-2004	6
No of interviewees from trials published between 2005-2010	23
No of interviewees from trials conducted through the MRC GPRF	19
No of interviewees from trials conducted through other research units	10
No of interviewees recruited from trials with loss-to–follow-up levels*:	
>20%	10
<20%	17

* Two nurses not linked to a sample frame trial

For peer review only

For peer review only - http://bmjopen.bmj.com/site/about/guidelines.xhtml

Figure 1 Questionnaire design features reported by interviewees to improve questionnaire return

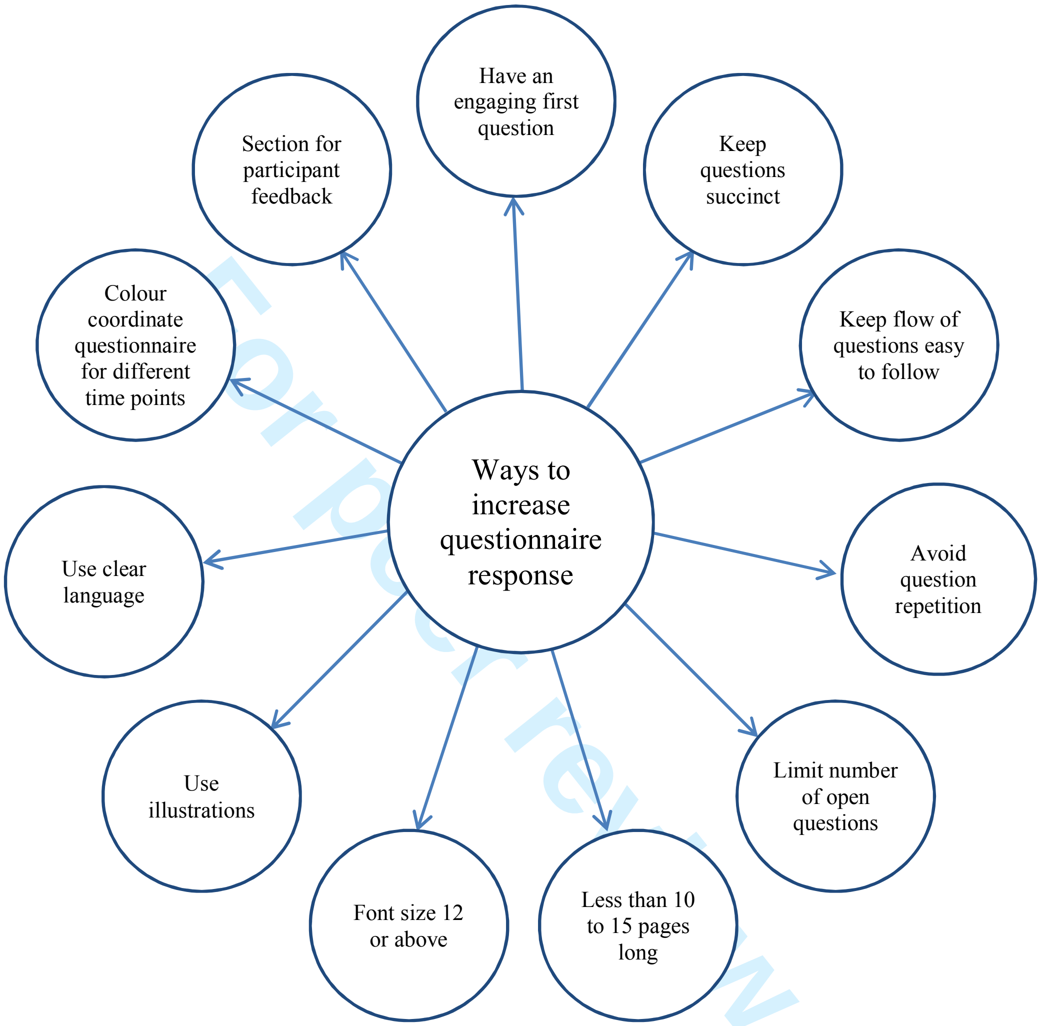

For peer review only

For peer review only - http://bmjopen.bmj.com/site/about/guidelines.xhtml

Web Appendix 1: Interview schedule

Background / introduction to the interview

I am interested in attrition from randomised trials. We define attrition as incomplete ascertainment of the primary outcome, but will subsequently use the term "loss to follow-up". I want to explore trialist's opinions on if and when loss to follow-up has been a problem in their experience and the strategies they may have used to deal with or to prevent loss to follow-up. I also want to find out more about trialists preferred strategies for preventing loss to follow-up.

Topic guide questions

1. **Can you tell me about recent trials you have worked on?**

    Were there any with high follow-up?

    Were there any with low follow-up?

2. **What do you think are the factors that lead to loss to follow-up in trials?**

    Why do you think it can be difficult to keep participants in randomised trials?

3. **What are the factors that lead to retention in randomised trials?**

4. **What strategies to increase follow-up have been successful for you in trials you have worked on?**

    Why do you think these have worked?

    What strategies have been unsuccessful?

    Why have these not worked?

Decision making around strategies used to reduce loss to follow-up

1. When do you perceive loss to follow-up to be a problem?
2. How do you decide which strategies to improve follow-up work best?
3. How did/ do you implement the strategies to improve follow-up that worked best?
4. How is loss to follow-up monitored in trials you have worked on?
5. Who deals with loss to follow-up when it presents?

Impact of research governance

1. What do you feel about using incentives to keep participants in trials?
2. Have you had any experience with ethics committees?
3. What do you feel about ethics committees asking about payments or giving other incentives to participants?
4. Has ethics committees approach to payments affected any trials you have worked on in anyway?

Ask this next question at the end of the interview

These are the strategies to reduce attrition/ improve retention identified by the Cochrane review.

For peer review only

Show participant each card separately (See below). Then ask the following questions for each strategy discussed:

1. Have you used these?
2. What do you think about using this strategy?
3. Might you have considered using this strategy in your trial?
4. What could be the advantages of using the strategy?
5. What could be the disadvantages of using this strategy?

Each card shown individually

Card no 1

Communication strategies. e.g. email, telephone, text messages, letters signed by different study personnel, type of delivery- e.g. post 1st 2nd class, or recorded delivery, type of envelope used for response.

Card no 2

Incentives to either participants or trialists e.g. gifts pens, pins, monetary incentives, offers of incentives, vouchers.

Card no 3

Methodological strategies blind versus un blind trials.

Card no 4

Different length of questionnaire: Short versus long.

Card no 5

Using case management. Having trial assistants manage participant follow-up, for example arranging transport and services to enable participants to keep trial follow-up appointments.

Card no 6

Motivational/educational strategies. Such as arranging workshops to give participants information about goal setting and time management.

For peer review only

Table 1 Characteristics of interviewees

**Characteristics**	**Number of interviewees**
Male	10
Female	19
**Role**	
Research nurse	9
Principal investigator	10
Trial manager	10
**Location**	
London	8
Midlands	8
Northeast England	3
Southwest England	3
East of England	5
Scotland	2
**Unit**	
University/Research organisation based	23
GP practice site based	6

Table 2 Other sample characteristics

**Other characteristics**	**Number of interviewees**
No of interviewees from trials published between 2000-2004	6
No of interviewees from trials published between 2005-2010	23
No of interviewees from trials conducted through the MRC GPRF	19
No of interviewees from trials conducted through other research units	10
No of interviewees recruited from trials with loss-to–follow-up levels*:	
>20%	10
<20%	17

* Two nurses not linked to a sample frame trial

For peer review only

Table 3 Use of retention strategies identified by the Cochrane review by primary care research team members

Strategy	Principal investigators	Trial managers	Research nurses
**Incentives**			
Monetary incentives	One PI had never used incentives	Used to increase response to postal questionnaires. Some split the incentive and gave participants a small incentive at different times e.g. £5 at randomisation and £5 at completion. Some send the incentive with the questionnaire; others on receipt of the questionnaire.	Mostly in pharmaceutical trials. Sometimes in academic trials.
Vouchers	Used in trials they lead	For those that had used vouchers, types used included: high street stores (M&S, ASDA, Tesco) online gift tokens (Amazon, Waterstones and WHS), or fresh fruit vouchers. Some had tailored the type of voucher to participant requests e.g. one interviewee used B&Q home improvement vouchers. These were sent by TMs with questionnaires or with a promissory element that on completion of questionnaires the participant would receive a voucher. Mobile phone top up vouchers used in trial to retain young people.	Administered at site level by research nurse. Used as a thank you from the trial team.
Gifts	One PI used gifts. Given for practical reasons i.e. carrying study materials	Used gifts. Seldom mentioned spontaneously. Used as reminders to keep trial at the forefront of participants and clinicians' minds or as a thank you for participants. Uncertainty about the effectiveness of gifts given on behalf of the team to either participants or clinicians.	RNs used gifts to retain people in randomised trials if prescribed. Seldom mentioned spontaneously. Used to increase recruitment rather than retention.
**Communication**			
Letter	Used in trials they lead	Uncertain of effectiveness. Trial managers signed letters sent from coordinating centre. Electronic and hand signatures used. Name of CI mentioned. Sometimes used letters as a stand-alone communication method.	Only used if specified in trial protocol/ study procedure manual.
Post	Used in trials they lead	2nd class used for pre-paid reply envelopes to reduce cost. 1st class used to send letters. Recorded delivery for second copy of questionnaire. Would confirm patient had moved. Hand delivered letters if participant difficult to contact.	Only used if specified in trial protocol/ study procedure manual. Sometimes hand delivered.
Telephone contact	Used in trials they lead	Used sometimes	Preferred method. Used for follow-up contact.
SMS texting	Used in trials they lead	Used when all other methods fail or if prescribed for follow-up	Used if prescribed. Seldom used.

For peer review only

Strategy	Principal investigators	Trial managers	Research nurses
Emails	Used in trials they lead	Used when email address given	Used when email address given.
New questionnaire formats	Some used shorter follow-up questionnaire with the second reminder.	Used a briefer follow-up questionnaire with a second reminder.	Short ones used for telephone interviews for non responders. Different lengths used for face to face interviews at site.
Behavioural	Not used	Not familiar with this	Not used
Case management	Not used	Used by one	Not used but aware of this
Methodology	Not used	Not used	Not used

For peer review only

Table 4 Speculation about the use of retention strategies identified by the Cochrane review in primary care randomised trials

Strategy	Principal investigators	Trial managers	Research nurses
**Monetary incentives**	Participation in pharmaceutical company trials paid. Payment could be perceived as bribery used to coerce participants, if the amount given is excessive e.g. >£50 acceptable to increase questionnaire response. Young people motivated by money. Older people participate for altruistic reasons. Some felt "cash in the hand" was a useful motivator. One suggested increasing the value of incentives for each questionnaire returned. Incentive values used £5 - £20 cash or vouchers. Considered reasonable for adults and children.	Could be perceived as bribery used to coerce participants, if the amount excessive >£50. Incentives are acceptable to increase questionnaire response. Uncertainty about when to administer incentives during follow-up. £5 - £20 considered reasonable for adults and children.	Could be perceived as bribery used to coerce participants, if amount is excessive i.e. >£50. Small incentives acceptable to increase questionnaire response. Participants should not be out of pocket. £5 - £20 considered reasonable for adults and children.
Vouchers	Some never use vouchers as incentives. Unclear if these were successful. Some thought vouchers might not be appropriate for some social groups. Generic vouchers most appropriate.	One TM never used vouchers. Unclear if these were successful. Vouchers thought to be less open to corruption, but a burden. Some TMs thought some vouchers might not be appropriate for some social groups. Generic vouchers are more appropriate.	Vouchers good for retention. Generic vouchers more have more universal application. Do not have to deal with cash at sites.
Gifts	Gifts not given on behalf of the study team by PIs. Interested to know what participants thought about gifts. Some thought these were patronising, a waste of money and associated with charity fundraising and participants might question the use of public money for this purpose. Some thought participants would prefer monetary alternatives. Participants would not participate in a trial for the gifts alone	For altruistic participants gifts could be perceived as "insulting" or "uninteresting". Some thought participants might be suspicious about the use of public money on gifts. This might be interpreted as wasteful or extravagant use of public money especially if motivated by altruism to participate. Could be associated with Charities and fund raising.	Gifts, offered or given, should be useful, appropriate, not patronising and tailored toward the disease/condition and participant group. One thought pens were "incidentals" not gifts. Felt gifts given would not retain participants in trials.
**Communication**			

For peer review only

Strategy	Principal investigators	Trial managers	Research nurses
Letter	Few comments on usefulness. Important factors tone and personalisation. Participant input important. Don't want to be seen as interfering in trial management. Mail outs boring and burdensome for trial managers.	Consistency of signatory more important than who signs. Name of CI mentioned. Some participants respond to authority. Franking with institutional logo used. Effect unknown. "Hate" brown envelopes, perceived as "bills" "junk mail" "letter from tax man". One thought no evidence that envelope colour mattered. Uncertain about hand written envelopes over preaddressed.	May not be opened by participants. Could get lost/ opened by someone else. Makes it look different with Logo. Might work.
Post	Not raised spontaneously by PIs	1st class used to send letters. More likely to be opened.	1st class post waste of money
Telephone	Involved in decisions about use of telephone contact. Calls should be conversational to build rapport with participant. Would be "daunting" if they heard from a PI. Young people change telephone numbers. Difficult to get hold of. Be careful not to harass participants.	Nurses use this, should use it more to keep in touch with participants, might increase retention. Reminder call before due to attend would increase retention. Calls may not be answered fear of telemarketing.	Prefer telephone to letters. Engage in dialogue. One thought telephone contact more intrusive letters. Be careful not to harass participants.
SMS texting	Good for young people. A system based on the automated NHS clinic appointments system would be useful. Need evidence it works.	Used for young people. Young people change numbers.	Good for young people. Utilising similar system to automated NHS clinic appointments system might be useful. Limited to people that can text
Email	Every mode should be considered	Used for people with busy lives	Good for people with busy lives
**New questionnaire formats**	One thought getting the balance right between length, readability, content and the acceptability of the topic were important. If this could be done without compromising what the questionnaire intends to measure. If questionnaire administered at clinic by nurse rather than post response could increase.	Long questionnaires considered off putting, no matter how engaged they were in a trial. Adding extra questions was another barrier to completion and non return. Having several short questionnaires could encourage return. Not so short that participants would not see the point of completion. Questionnaires administered at the clinic by a nurse rather than by post could increase response. The time at which questionnaires are posted was felt to be a factor associated with improved response. Questionnaires sent before a clinic visit enables the participant to complete a	Shorter questionnaires of 30-50 pages were thought to be long and off putting for participants to complete, no matter how engaged they were in a trial. The addition of extra questions that were not often analysed was another barrier to completion and non return. Having several short questionnaires was thought to encourage return, these should not be so short that trial participants would not see the point of completing them. People get used to questionnaires.

For peer review only

Strategy	Principal investigators	Trial managers	Research nurses
		questionnaire at home before their clinic visit.	
Behavioural	Don't understand it. May contaminate intervention. Generalisability of results questionable. "*Preachy*". Need evidence. Sounds like giving them a bit of the intervention. Could lessen effects of the treatment intervention.	Work shops about motivation condescending to participants. Don't know if it would work. Time consuming and expensive. May work with some groups. Don't like it and don't know much about it. Could not imagine using it. May be a burden for coordinators and participants.	Not heard of this. Workshop potential problems: non attendance, burden for participants and organisers, time commitment.
Case management	Where the intervention has to be reproduced could be problematic. Useful if there is not much benefit for participants. Takes time to arrange. Expensive. Annoying if participant does not turn up. Would not have thought of doing it. We do some of this.	Important for elderly. Costly. Should be done, rarely have luxury to do it. Should not be a substitute for good communication. Degrees of case management used. Logistics need working out.	Could be useful. Some heard about this. "*Cannot fail*", expensive, logistics need to be worked out. Useful for elderly. Some do elements of this. Personality of case manager important.
Methodology	Could not blind some participants to e.g. therapist lead interventions. Depends on study. Could bias results, blinding should be done where possible. Not suitable as a strategy to reduce attrition.	Do not know enough and never thought about it. Risks: would drop out if open and did not get allocation to preferred treatment.	Don't know enough about this. Participants could drop out if not in preferred allocation group.

For peer review only

Box 1 Factors thought to contribute to retention in primary care trials

Reducing the burden of participation for participants

"The burden of activity on the patients, how easy it is for them, how long it's going to take, how intrusive in terms of blood samples or answering huge long questionnaires, it's partly the complexity as well, if it's very simple for them to do, often you just want very simple data back from people, alive or dead, or specific complications, if it can be done either with a, increasingly with a click of a button by sending a text or an email to people or just a very you know, reply paid cards so you're just getting the data back from them, I suppose the other thing is….trying to do electronic follow-up as much as possible so you don't actually have to bother the patient, you have their consent to access their electronic records and either their Primary care data can be interrogated …a way of linking all of those together, like with Cancer Registry, Registrar General data, so I suppose the more you can automate it the easier it becomes".

Principal investigator interview 4

Establishing a rapport with participants

"I think with a lot of studies the rapport with whoever, the sort of teams that they're engaging with, you know, be that the GP and the practice nurse or be it a consultant and a research nurse, if they show interest and passion and enthusiasm for the study that's really, really helpful and then if they're sort of treated well when they come for the follow-up visits……"

Principal investigator interview 28

Effective communication with participants

"I think it is communication, communication at all levels; so the nurse who interfaces with the trial management team behind the coordinating centre, …but her or him listening to the participant's views as well and … feeding them back, so ……. a two-way process. Because we're this side doing the research, we don't know best, we have to listen to… which is, I guess, why we involve consumers in our work as well, so yes, just communication and understanding your population that you're working with".

Trial manager interview 13

"We …..mak(e) sure that we have [communication] strategies in place to get as many …..responses to our postal questionnaires …we send out the questionnaire…, if we don't hear back ….we will ring up to say …."Could you possibly send it back to us?" If we still don't hear …we'll send .. another copy … if we don't have a response from that copy then we'll ring them again…if we still don't hear we've telephoned …again to collect a set of core outcomes to get a response………..".

Trial manager interview 14

Personality of trial research staff

"[Being] friendly is very important. And I just think having the right approach to things in general. So

For peer review only

you have a 'can do' attitude rather than a inflexible attitude. You have to have somebody who... Or people who... people who are prepared to go out of their way to do things. And have a very person focused way of doing things."

Trial manager interview 26

"If you're enthusiastic, and can give the information to the participants with,…interest and,… also sympathetic to their, …. queries, always being accommodating, always getting back to them if they have any queries if, there are messages left, being absolutely spot on with, so that they don't feel they've been let down or you haven't been bothered to get back to them."

Research nurse interview 5

"I think there's a paradox here because they [trial managers] need to be obsessional about detail but they also need to be relaxed and flexible in their response to different situations ….that come up, different problems that come up. That's asking quite a lot, I think. So you know it's great if you find somebody who's good at it…..Or be very kind hearted. And prepared to work out of hours!"

Principal investigator interview 25

Flexibility of trial research staff

"They're [participants] do[you] a favour if they are taking part in a clinical trial so we need to try to be flexible, come very early morning …or stay later or they can come after work. Lunch time if it is, you know near to their work but of course like sometimes people work in the city and is away from where they live or it's a long journey. ….If a patient sees that they're going sit there for an hour and a half he says "I can't, I can't afford the time", ….. you get that, you know, quite often…"

Research nurse interview 12

Altruism of participants

"I've always been of the opinion that (…..)healthy volunteers, … they have what can be considered a less of a kind of vested interest in the study (…) they're doing it all out of the goodness of their hearts, there's not a lot in it for them"

Trial manager interview 26

Tailoring strategies to different follow-up groups

"Depending on the trial and the length of the trial and your client group and all those sort of things…you would have a completely different strategy for, you know, …..thinking of Chlamydia, the fifteen to twenty-four year olds, [compared] to something like [a trial involving the elderly] where you're looking at the seventy-five plus….. you do sort of tailor it [the strategy] to your group."

Research Nurse interview 2

\

For peer review only

Box 2 Factors thought to contribute to loss to follow-up in primary care trials

Participant age

Younger participants

"A young girl whose parents had agreed to be in the trial and then, you know, she didn't want to and then decided herself she was going to withdraw. And, you know, I can see the potential for that sort of thing if it's a trial in the sort of pre-teen, teen group that,... they might well change their mind….more interesting things going on in their lives, they've got more after school activities or they've got exams or they've got whatever. And coupled with being more independent and wanting to assert their own ideas".

Principal investigator interview 28

Older participants

"In trials with older people, people will die, people will get frailer and maybe be institutionalised and not be able to continue or not want to continue for those sort of reasons".

Principal investigator interview 28

"There are all the usual things, we assume that older people don't move, there's a astonishingly high level of mobility in the older population. So lost to follow-up can occur just because people say "right, I'm going to go and live in…………." or "I'm going to go and live near my daughter" or "I've off to ……….for the winter". [They] do… a lot of child care, grandparental child care and sorting out their children's divorces and so on and so they get distracted and drop out completely through distraction".

Principal investigator interview 21

Disease/condition health status

"Bi-polar groups, a lot of them find it very, very difficult going to an NHS facility".

Trial manager interview 17

"it depends on what area of medicine it is, for instance, if you're dealing with people with psychiatric illness, there's always a problem with attendance, whether that's within the surgery or whether that's within research".

Research nurse interview 1

"In nutrition studies we're very much bound by looking at healthy individuals to begin with. So from that perspective they may certainly be less motivated than an individual participating in a drug trial who has a severe or debilitating condition that, you know, they want to try and do something about".

Trial manager interview 15

Research environment at the general practice

"if somebody genuinely did want to make the trial work then it was possible to make it work. ……………….it needed somebody to actually manage the trial within that practice. The practices in which we had success I felt that we had a champion there who was fighting our corner for us"

Trial manager interview 27

For peer review only

"the other thing is practice awareness, you know, because you said it's a big practice and if somebody comes in and hasn't got the sort of clock-in bit straight and they go to the desk and say, I've come to see X about this trial and, you know, new receptionist knows nothing. That can be quite off-putting so as long as everybody's engaged …and everybody knows about it".

Research nurse interview 2

For peer review only

Figure 1 Questionnaire design features reported by interviewees to improve questionnaire return

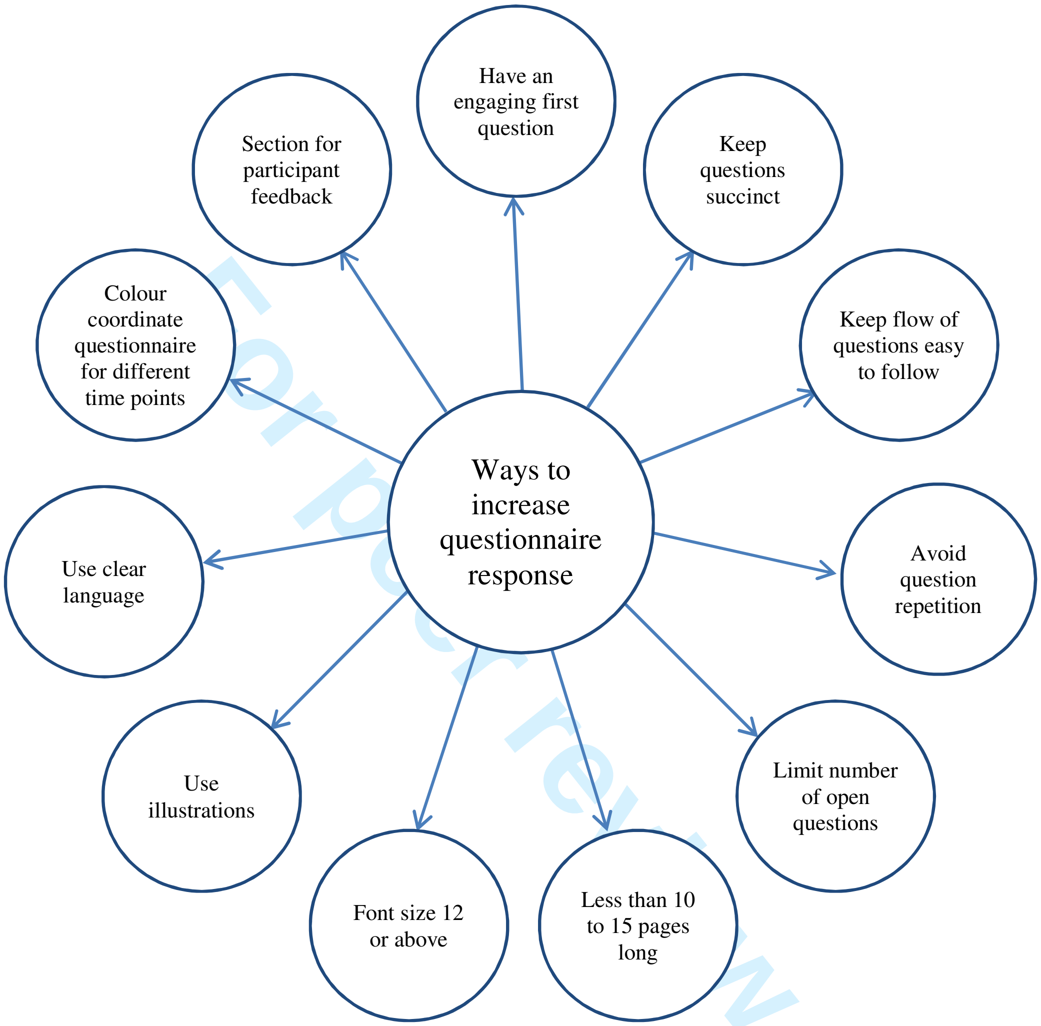

For peer review only

Web Appendix 1: Interview schedule

Background / introduction to the interview

I am interested in attrition from randomised trials. We define attrition as incomplete ascertainment of the primary outcome, but will subsequently use the term "loss to follow-up". I want to explore trialist's opinions on if and when loss to follow-up has been a problem in their experience and the strategies they may have used to deal with or to prevent loss to follow-up. I also want to find out more about trialists preferred strategies for preventing loss to follow-up.

Topic guide questions

1. **Can you tell me about recent trials you have worked on?**

    Were there any with high follow-up?

    Were there any with low follow-up?

2. **What do you think are the factors that lead to loss to follow-up in trials?**

    Why do you think it can be difficult to keep participants in randomised trials?

3. **What are the factors that lead to retention in randomised trials?**

4. **What strategies to increase follow-up have been successful for you in trials you have worked on?**

    Why do you think these have worked?

    What strategies have been unsuccessful?

    Why have these not worked?

Decision making around strategies used to reduce loss to follow-up

1. When do you perceive loss to follow-up to be a problem?
2. How do you decide which strategies to improve follow-up work best?
3. How did/ do you implement the strategies to improve follow-up that worked best?
4. How is loss to follow-up monitored in trials you have worked on?
5. Who deals with loss to follow-up when it presents?

Impact of research governance

1. What do you feel about using incentives to keep participants in trials?
2. Have you had any experience with ethics committees?
3. What do you feel about ethics committees asking about payments or giving other incentives to participants?
4. Has ethics committees approach to payments affected any trials you have worked on in anyway?

Ask this next question at the end of the interview

These are the strategies to reduce attrition/ improve retention identified by the Cochrane review.

For peer review only

Show participant each card separately (See below). Then ask the following questions for each strategy discussed:

1. Have you used these?
2. What do you think about using this strategy?
3. Might you have considered using this strategy in your trial?
4. What could be the advantages of using the strategy?
5. What could be the disadvantages of using this strategy?

Each card shown individually

**Card no 1**<br>Communication strategies. e.g. email, telephone, text messages, letters signed by different study personnel, type of delivery- e.g. post 1st 2nd class, or recorded delivery, type of envelope used for response.

**Card no 2**<br>Incentives to either participants or trialists e.g. gifts pens, pins, monetary incentives, offers of incentives, vouchers.

**Card no 3**<br>Methodological strategies blind versus un blind trials.

**Card no 4**<br>Different length of questionnaire: Short versus long.

**Card no 5**<br>Using case management. Having trial assistants manage participant follow-up, for example arranging transport and services to enable participants to keep trial follow-up appointments.

**Card no 6**<br>Motivational/educational strategies. Such as arranging workshops to give participants information about goal setting and time management.

For peer review only

Use of strategies to improve retention in primary care randomised trials: a qualitative study with in-depth interviews

Journal:	*BMJ Open*
Manuscript ID:	bmjopen-2013-003835.R2
Article Type:	Research
Date Submitted by the Author:	29-Nov-2013
Complete List of Authors:	Brueton, Valerie; MRC Clinical Trials Unit, Meta-analysis Group<br>Stevenson, Fiona; University College London, Research Department of Primary Care and Population Health<br>Vale, Claire; MRC Clinical Trials Unit, Meta-analysis Group<br>Stenning, Sally; MRC Clinical Trials Unit, Cancer and non infections Group<br>Tierney, Jayne; MRC Clinical Trials Unit, Meta-analysis Group<br>Harding, Seeromanie; MRC Social and Public Health Sciences Unit,<br>Nazareth, Irwin; UCL, Primary Care and Population Health<br>Meredith, Sarah; MRC Clinical Trials Unit,<br>Rait, Greta; University College London, Primary Care and Population Health
**Primary Subject Heading**:	Research methods
Secondary Subject Heading:	General practice / Family practice, Qualitative research
Keywords:	Retention strategies, Randomised trials, PRIMARY CARE

SCHOLARONE™ Manuscripts

For peer review only

Use of strategies to improve retention in primary care randomised trials: a qualitative study with in-depth interviews

Brueton V

Research Scientist, Meta-analysis Group
MRC Clinical Trials Unit at UCL
Aviation House
125 Kingsway
London WC2B 6NH
e-mail: vcb@ctu.mrc.ac.uk

Stevenson F

Senior Lecturer
UCL Research Department of Primary care and Population Health
Royal Free and University College Medical School
Rowland Hill Street
London
NW3 2PF

Vale CL

Senior Scientist Meta-analysis Group
MRC Clinical Trials Unit at UCL
Aviation House
125 Kingsway
London WC2B 6NH

Stenning SP

Senior Statistician
MRC Clinical Trials Unit at UCL
Aviation House
125 Kingsway
London WC2B 6NH

Tierney JF

Senior Scientist Meta-analysis Group
MRC Clinical Trials Unit at UCL
Aviation House
125 Kingsway
London WC2B 6NH

Harding S

Program leader: Ethnicity and Health

For peer review only

BMJ Open qualitative paper submitted Version 3 VB 26.11.13

MRC Social & Public Health Sciences Unit
University of Glasgow
4 Lilybank Gardens
Glasgow G12 8RZ

Nazareth I

Professor of Primary Care
PRIMENT Clinical Trials Unit
Research Department of Primary care and Population Health
UCL Medical School
Rowland Hill Street
London
NW3 2PF

Meredith S

Deputy Director
MRC Clinical Trials Unit at UCL
Aviation House
125 Kingsway
London WC2B 6NH

Rait G

Clinical Senior Lecturer
PRIMENT Clinical Trials Unit
Research Department of Primary care and Population Health
UCL Medical School
Rowland Hill Street
London
NW3 2PF

For peer review only

BMJ Open qualitative paper submitted Version 3 VB 26.11.13

Abstract

Objective

To explore strategies used to improve retention in primary care randomised trials.

Design

Qualitative in-depth interviews and thematic analysis.

Participants

Twenty nine UK primary care chief and principal investigators, trial managers and research nurses.

Methods

In-depth face to face interviews.

Results

Primary care researchers use incentive and communication strategies to improve retention in trials, but were unsure of their effect. Small monetary incentives were used to increase response to postal questionnaires. Non-monetary incentives were used although there was scepticism about the impact of these on retention. Nurses routinely used telephone communication to encourage participants to return for trial follow-up. Trial managers used 1st class post, shorter questionnaires and improved questionnaire designs with the aim of improving questionnaire response. Interviewees thought an open trial design could lead to biased results and were negative about using behavioural strategies to improve retention.

There was consensus among the interviewees that effective communication and rapport with participants, participant altruism, respect for participant's time, flexibility of trial personnel and appointment schedules, and trial information improve retention. Interviewees noted particular challenges with retention in mental health trials and those involving teenagers.

Conclusions

The findings of this qualitative study have allowed us to reflect on research practice around retention and highlight a gap between such practice and current evidence. Interviewees describe acting from experience without evidence from the literature, which supports the use

For peer review only

of small monetary incentives to improve questionnaire response. No such evidence exists for non-monetary incentives or first class post, use of which may need reconsideration. An exploration of barriers and facilitators to retention in other research contexts may be justified.

Article Summary

Article focus

High levels of retention in trials are important to maintain power and minimise bias. Strategies to improve retention are widely used in trials but without evidence of effect. Strategies thought to improve retention in UK primary care randomised trials have not been well documented or evaluated. This study explores the use, in UK primary care trials, of strategies to improve retention.

Key messages

Small monetary incentives, non-monetary incentives and communication strategies are used in primary care trials to improve retention. Behavioural, methodology and case management strategies were not used. Small monetary incentives and communication strategies were thought to be useful for retention and case management thought to be potentially useful. Interviewees were negative about the use of behavioural, methodology and non-monetary incentive strategies.

Factors thought to contribute to retention are: effective communication, participant altruism and perceived benefits of participation for participants, burden reduction, personality and flexibility of trial staff.

Factors thought to contribute to loss to follow-up are: lack of time and interest in the trial, physical constraints, and limitations of research facilities.

Strengths and limitations

This study is the first to explore the use of retention strategies in UK primary care trials and the factors associated with retention and loss to follow-up. Interviews were conducted with researchers experienced in UK primary care trials, many of whom had used strategies to improve retention.

For peer review only

A limitation is that the thoughts and experiences of trial participants have not been gathered to further explore the barriers and facilitators to retention in primary care trials.

For peer review only

For peer review only - http://bmjopen.bmj.com/site/about/guidelines.xhtml

Introduction

Retention in primary care randomised controlled trials conducted across different disease areas and communities can be challenging. Inadequate retention can reduce the power of a trial and introduce bias, particularly if drop-out differs across trial arms. Reasons for loss to follow-up can include a change in the participants' location, withdrawal from treatment and / or loss of commitment to the trial, for example due to complicated treatment regimens[1]. A Cochrane review of strategies to improve retention in trials demonstrated that adding a monetary incentive and offering higher valued monetary incentives increased postal and electronic questionnaire response[2]. Questionnaire response was also increased by recorded delivery of questionnaires, a "package" of postal communication strategies known as the Total Design Method (TDM)[3] and an open trial design[4], although these were based on the results of single trials. The evidence of an effect for shorter questionnaires and questionnaires relevant to the disease / condition was less clear. Also, there was no good evidence that the following strategies improved retention: adding or offering a non-monetary incentive, communication strategies (including use of first class mail), behavioural motivational strategies, new questionnaire formats and case management.

Only six of the 38 retention trials included in the Cochrane review were embedded in UK primary care trials and all targeted improving response to postal questionnaires in order to improve retention[5-8]. However, primary care trials also require participants to return to sites for clinical outcome measurement to assess disease progression[9;10]. There could be important factors that contribute to the use of strategies to improve questionnaire response and for participants to return to sites for follow-up in the context of primary care trials.

We conducted a qualitative study with primary care trial personnel to explore the strategies generally used to improve retention in primary care randomised trials and to map these to the results of our Cochrane review[2].

Methods

Sampling the study population

To explore the use of strategies to improve retention in randomised primary care trials, in-depth face to face interviews were conducted with principal / chief investigators (PIs), trial managers (TMs) and research nurses (RNs) purposively sampled from a sampling frame of

For peer review only

primary care randomised trials published from 2000 - 2010. The trials were identified from either the MRC General Practice Research Framework (GPRF) database of clinical research projects, websites of UK primary care research units or from hand searches of trials published between 2009 - 2010 in *Lancet, British Medical Journal, Family Practice* and *British Journal of General Practice*. The trial publications identified spanned different disease areas, research units, and levels of loss to follow-up (Tables 1 and 2) and were stratified by levels of loss to follow-up (≤20%, ≥20%), and publication date (2000 - 2004 and 2005 - 2010). All PIs, TMs and RNs were identified for recruitment from the list of authors associated with each trial in the sampling frame or through records of trial staff associated with MRC GPRF primary care trial publications. In addition, TMs were identified through the Trial Managers Network[11]. TMs recruited in this way had to be associated with a randomised primary care trial published between 2000 - 2010 which was subsequently entered into the sampling frame.

Data collection

Ethics approval was granted by University College London Research Ethics committee (application no 2342/002). Potential interviewees were sent an information sheet and recruited via email. Prior to the interview participants were given study information and asked to sign a consent form. Face-to-face interviews explored everyday retention practices and the factors thought to contribute to retention in primary care trials. If retention strategies found by the Cochrane review[2] (i.e. incentives, communication methods, new questionnaire formats, behavioural, case management and methodological strategies) were not mentioned spontaneously by interviewees, they were asked specifically about these (Interview schedule Appendix 1). Interviewees were also asked specifically about the role of the ethics approval process on the use of monetary incentives to improve retention in primary care trials.

The interviews were conducted by one author (VB) in a place convenient to the interviewee they were recorded digitally and took approximately one hour. Transcripts were checked following each interview for flow and usefulness of results and the interview schedule modified accordingly. Appropriate probes were used to allow participants to expand on issues around the use of strategies to improve retention.

Data management and analysis

For peer review only

We conducted a thematic content analysis. The textual data were managed using Atlas ti 6. A transcript review group was formed with four authors (VB, CV, GR, FS) and was heterogeneous in terms of the members' professional background. Each transcript was reviewed independently by at least two group members who documented the emerging major themes which were then discussed by the group in pre-planned meetings. This group met monthly while data were collected. There was a high degree of convergence in the themes identified.

Data were also analysed iteratively so early results could be incorporated and probed in later interviews to increase the depth of the findings. Labels were agreed and used as broad codes to label textual data associated with major themes. Sub codes were identified discussed and agreed between two authors (FS, VB) and also used to label textual data. Transcripts were subsequently coded by one author (VB). The first two transcripts were checked by another author (FS). A key consideration in coding was to mark data produced spontaneously and those which were specifically asked about, therefore data were coded to take account of the response and the question that prompted that response in order to apply appropriate emphasis on responses. Codes for the six strategies identified by the Cochrane review i.e. *"communication", "incentives", "questionnaires", " methodology", "case management"* and "*behavioural"* were used as a priori codes for the later part of the interview transcripts as participants were asked specifically about the use of Cochrane review strategies.

Textual data relating to retention strategies were subsequently retrieved from a coded database and constantly compared within and across the three groups of trial personnel (i.e. PIs, TMs, RNs) to identify the use of each strategy and to document the reported advantages and disadvantages of each. Any factors thought by the interviewees to contribute to either retention or loss to follow-up were also coded and retrieved for analysis[12;13]. Relevant quotes, representing the interviewee's views, were selected to illustrate the results. Interviews were conducted between August 2010 and May 2011 until no new content or themes emerged.

Results

Fifty four trial personnel were identified for recruitment from 37 randomised primary care trials from England and Scotland included in the sampling frame. Eleven of the fifty four invitees declined to participate (PIs (n=10), TMs (n=1)). Seven of these PIs recommended another co-author to invite. Fourteen of the fifty four did not respond (PIs (n=1010), TMs

For peer review only - http://bmjopen.bmj.com/site/about/guidelines.xhtml

(n=3), RNs (n=1. Ten PIs, ten TMs and nine RNs were recruited from 23 of the 37 primary care trials identified.

Interviewees were involved in running randomised primary care trials conducted in nutrition, health promotion, neurology, gynaecology, mental health, musculoskeletal, ear nose and throat (ENT), respiratory, endocrine medicine and minor medical conditions. Collectively their experiences, which were not limited to the trial from which they were sampled, included trial design, implementation, coordination and data collection at GP practice sites by post, face to face and electronic methods. Trial publications reported loss to follow-up rates between 5% - 39%. Twenty interviewees were from trials with ≤20% loss to follow-up reported (See Tables 1 and 2). Different types of follow-up were used in these trials: face-to-face follow-up at clinics (n=20), postal questionnaires (n=6), follow-up at home (n=2) or self-completion diaries (n=2). Combinations of follow-up types were also used: clinic visits or postal follow-up (n= 4), telephone and postal follow-up (n=2), registry data and follow-up at home (n=1).

The PIs were responsible for the design and implementation of trials, including research governance and ethics approval applications. Nine PIs were GPs with academic roles, and one was a non-clinical senior academic. The TMs were based in academic research units and managed trial data collection via telephone, post, email and SMS text message. TMs also coordinated and monitored data centrally and communicated with trial sites. Six RNs coordinated clinical research at GP practice sites. They collected biomedical and clinical data by telephone and face-to-face interview, and communicated with the coordinating centre and with PI clinicians at sites. Three RNs were based at a primary care research network coordinating centre where they answered clinical data collection queries and monitored data quality.

Results are presented first on the interviewee's experiences of and perspectives on using communication, incentive and new questionnaire strategies, identified by the Cochrane review[2], to improve primary care trial retention. This is followed by views on other less frequently evaluated strategies identified by the Cochrane review[2] i.e. behavioural, case management, and methodological strategies. Factors thought to contribute to retention and loss to follow-up in primary care trials are also presented.

For peer review only - http://bmjopen.bmj.com/site/about/guidelines.xhtml

The use of retention strategies identified by the Cochrane review

1. Use of incentives

Monetary incentives

Monetary incentives and offers of monetary incentives were mentioned spontaneously by most PIs and TMs as useful strategies to improve response to postal questionnaires. Over half of the PIs interviewed had used monetary incentives given in either cash or voucher format to increase questionnaire return, although there was uncertainty about their effect. The different types used were high street vouchers and mobile phone vouchers, but vouchers were thought of as a burden to administer for trial management teams. Generic vouchers were thought to be more acceptable to all trial participant groups. It was felt that the incentives should be seen by trial participants as a "*thank you*" rather than as payment for participation.

The monetary value of the incentives used was between £5 and £20 and this amount was considered reasonable for adults and children. Larger monetary incentives (>£50) were perceived as coercion or bribery by interviewees across the three groups interviewed. Offers of entry into a prize draw, although seldom used as a strategy to improve questionnaire response, were thought by some interviewees to be a cost effective alternative to giving a monetary incentive.

There was uncertainty among TMs about effective ways to administer incentives during follow-up to optimise questionnaire response. Some gave a small incentive at different follow-up time points e.g. £5 at randomisation and £5 at trial completion. Others sent an incentive with the questionnaire, or on receipt of a completed questionnaire.

TMs and PIs sampled from trials with ≤20% loss to follow-up appeared to use more strategies to improve retention than those from trials with ≥20% loss to follow-up. For example those in the ≤20% group gave incentives at different time points to try to keep the participants motivated to return questionnaires. TMs and PIs sampled from trials with ≥20% loss to follow-up seemed more cautious about the use of incentives and some felt participants could feel coerced if incentives were used to improve follow-up.

Non-monetary incentives

For peer review only - http://bmjopen.bmj.com/site/about/guidelines.xhtml

Non-monetary incentive strategies or gifts i.e. pens, certificates of appreciation, key rings and mugs were sometimes used in an attempt to improve both participant recruitment and retention. These were used to market the trial, and in the case of pens, to encourage and remind participants to return completed questionnaires. Interviewees thought that these gifts could be perceived by trial participants as patronising, not useful, or associated with charity fundraising and also as a waste of public money, especially if the participant had altruistic motivations for participation.

> *"I think people are very sensitive about money especially in the public sector, ...if they see something that could be considered wasteful or extravagant, I don't think it would go down too well. Because...... if you get involved in a study that's being funded from public funds and from charity, you know, it's quite an altruistic thing to do, I don't think people are looking to be rewarded for it ....then they see you wasting money on pens and mugs or, I don't think it would go down very well".*
>
> ***Trial manager interview 7***

Some interviewees thought that the money used to finance such non-monetary / gift incentives could be better used to subsidise follow-up visits for participants. Indeed there was consensus amongst interviewees about the importance of reimbursing travel and parking expenses associated with follow-up visits. Some interviewees differentiated this from giving incentives to participants. Others recounted cases where participants did not want to be reimbursed particularly if they had altruistic motivations for participation, and others felt that an offer of money to cover expenses could make participants feel valued. There was a general feeling that this varied by participants' socio-economic circumstances e.g. it was thought that participants from higher socioeconomic groups were less likely to accept a monetary incentive.

> *"We actually gave them ten pounds for their travel costs, once at the beginning and once at the end.. and a lot of them didn't want it, you know, they'd say, oh... And I'd say, well just pop it in your local charity".*
>
> ***Research nurse interview 2***

Ethical approval for the use of incentives

Interviewees across all groups thought it was important that ethics committees sought justification for the use of incentives in trials. They felt that opinion varied among ethics committees about the use of incentives to improve retention but overall they were now more

For peer review only - http://bmjopen.bmj.com/site/about/guidelines.xhtml

accepting of the use of small monetary incentives and of reimbursing participants' trial related expenses. Some PIs said that it was helpful to know who was on the ethics committee and that approval to use incentives is generally granted when a clear justification for use is given. Others thought incentives were likely to be approved if they were used to pay participant travel expenses.

2. Use of communication strategies

Communication strategies, for example contact with participants by telephone, letter and email during follow-up, were spontaneously mentioned and routinely used to improve trial retention. RNs and TMs used these as first line retention strategies.

Telephone calls

RNs frequently used telephone calls, to arrange or re schedule trial follow-up appointments or to remind participants to return postal questionnaires. Both RNs and PIs were conscious not to harass participants with too many telephone reminders. TMs thought reminder calls to participants prior to follow-up appointments might increase follow-up attendance. A telephone reminder to a site to remind the RN that a participant is due for a follow-up appointment was also thought to potentially improve retention. PIs did not contact participants by telephone and one thought it would be "*daunting*" for a participant to receive a call from a PI. Nevertheless, PIs were involved in decisions about when to use telephone contact, especially when they thought that participants were fatigued by providing data in diaries or repeat questionnaires.

Letters

Letters were used predominantly by TMs to communicate with trial participants, but there was uncertainty about their effect. Electronic coloured signatures, franked institutional logos placed on outward envelopes, different coloured envelopes, and first class outward post were used to attract the attention of participants.

Letters were usually signed by TMs rather than PIs, the TMs thought that consistency in the signatory was more important than the status of the signatory for improving questionnaire response. Some TMs sent letters to participants in time for weekend delivery, but were unsure whether this influenced response. PIs thought that letters that were personalised, and the tone of the letter were important for retention or improving questionnaire return, but that the

For peer review only

For peer review only - http://bmjopen.bmj.com/site/about/guidelines.xhtml

associated large mail-outs were burdensome for trial staff. Greeting cards were used occasionally to maintain communication with participants, however, interviewees were negative about the impact of these on retention and questionnaire response, with the exception of the use of birthday cards in paediatric trials, and Christmas cards sent to sites from coordinating centres.

> *"I'm routinely suggesting that we send out birthday cards to children. We've done Christmas cards to participants on behalf of the trial team but there's no evidence that it works, but I think that it's a gesture and it's in the right direction, it's better than nothing".*
>
> ***Trial manager interview 27***

There was uncertainty about the effect on retention / response of the different types of postal delivery they use to send trial materials to participants. First class post was used to send trial materials to participants and 2nd class post for questionnaire return. While some TMs and RNs thought that 1st class post was a waste of financial resources, other TMs thought 1st class stamped letters were more likely to be opened by participants. Recorded delivery was occasionally used to send important study-related materials, or to resend questionnaires to non-responders. Some thought recorded delivery was useful to find out if a participant had changed address, but others thought recorded delivery could be inconvenient e.g. if the participant had to collect trial supplies from a post office.

Some TMs noticed that retention improved when questionnaires were sent before clinic visits as this enabled participants to complete the questionnaire at home beforehand. A prepaid envelope for return of postal questionnaires was thought to reduce the cost of postage to participants and the inconvenience of having to buy a stamp. To minimise respondent burden, clear instructions on how to return the questionnaire once it is completed was thought by TMs to contribute to improved postal questionnaire response.

Electronic communication

TMs and RNs used emails to contact participants who preferred this approach. SMS text messaging was used less so but most interviewees thought that it would be useful for communicating with young people. The use of an automated system for texting appointment reminders similar to that used by the NHS for clinic appointments was thought to be a

For peer review only - http://bmjopen.bmj.com/site/about/guidelines.xhtml

potentially useful way to improve trial retention. However one RN reported that the reminders sent via the system did not improve clinic attendance.

Newsletters

Most interviewees thought newsletters were useful for keeping participants and site clinicians informed about trial recruitment, retention, and general trial news. Others thought these were less useful and could bias trial results by contaminating and confounding the results of the *treatment as usual* group. The frequency with which newsletters were sent to participants varied from fortnightly to annually, and depended on the condition / disease, intervention, and length of follow-up. Some PIs thought having trial-specific information and answers to frequently asked questions as a resource on a website could benefit retention.

3. Use of new questionnaire formats

None of the interviewees spontaneously mentioned modifying a follow-up questionnaire to improve response or retention, although some PIs and TMs had used shorter questionnaires for non-responders. When asked specifically about the impact of questionnaire length on retention, interviewees expressed a preference for shorter questionnaires, which they thought increased response. Interviewees also thought that long questionnaires administered frequently led to "*questionnaire fatigue*", and some thought that dividing long questionnaires into several short questionnaires could be useful to reduce the burden of data collection on participants. However, some PIs thought that if the questionnaire content was of interest to the participant, then the length of the questionnaire may not affect response. TMs and PIs thought the involvement of patient representatives in the design and pre-test of questionnaires could improve retention. Many suggestions were also offered for improving questionnaire design in order to increase questionnaire response (Figure 1).

How a questionnaire was administered and by whom was also thought to affect questionnaire response and retention. Some PIs and TMs thought that if data were collected at the clinic site by a nurse rather than by post that this could increase response.

4. Use of other strategies to improve retention

Other strategies identified by the Cochrane review[2] were not routinely used or mentioned spontaneously by interviewees as ways to improve retention. Opinions were mixed about the usefulness of a behavioural strategy where participants are given information about goal

For peer review only

setting and time management to facilitate successful trial completion; methodological strategies where a blind trial design was compared to an open / unblind trial design; and case management where trial assistants manage participant follow-up, by arranging services to enable participants to keep trial follow-up appointments.

Behavioural strategies were described to interviewees as *"Arranging workshops to give participants information about goal setting and time management"* for example in the context of an exercise behaviour change intervention. When asked specifically about the use of such a strategy, interviewees were generally negative. Such strategies were discussed in the following ways: "*strange*", "*preachy", "condescending*", "*never came across it", "would never think of doing it*", *I'd be surprised if it worked*" and "*would need evidence that it worked*". Some thought that a behaviour change retention strategy could not be used because it may contaminate a behaviour change trial intervention and therefore may affect the generalisability of trial results.

Although the interviewees were familiar with the concept of blind and open trials, they were not aware of the use of either design as a strategy to improve trial retention. Interviewees felt that using an open trial design to improve retention could bias results, particularly as blinding is used to reduce bias in trials. They also felt that a blind trial design could not be applied to therapist-led behavioural interventions

One TM had used case management to improve trial retention. This involved the TM arranging services to enable participants to attend trial follow-up appointments. The interviewees generally thought that case management could improve trial retention with some groups e.g. the elderly, disabled and participants with low incomes but they also thought this could create dependence on the service. The impact of this strategy on retention was thought by some to depend on the personality of the case manager.

Factors thought to influence trial retention

In addition to the strategies employed to improve retention, interviewees also mentioned other factors that had both positive and negative influence on retention:

1. Participants

For peer review only - http://bmjopen.bmj.com/site/about/guidelines.xhtml

A key factor thought to optimise retention was to reduce the burden of follow-up for participants in terms of the length of time they spend in follow-up activities and the type of data collected (e.g. long questionnaires, biomedical specimens). More convenient ways to collect outcome data, e.g. online data collection were thought to be more effective for retention. Reducing the burden of follow-up visits by streamlining appointments or finding more convenient ways to collect key data (e.g. on line or by telephone) were also considered important for retention.

> *"The burden of activity on the patients, how easy it is for them, how long it's going to take, how intrusive in terms of blood samples or answering huge long questionnaires, it's partly the complexity as well, if it's very simple for them to do, often you just want very simple data back from people, alive or dead, or specific complications, if it can be done either with a, click of a button by sending a text or an email to people or just …reply paid cards so you're just getting the data back from them, ..the other thing is……… to do electronic follow-up as much as possible so you don't actually have to bother the patient, you have their consent to access their electronic records and either their Primary care data can be interrogated …".*
>
> ***Principal investigator interview 4***

The time participants spend getting to, from, and at follow-up visits, giving participants adequate time at follow-up visits, and ensuring that participants were not kept waiting too long for follow-up appointments were all considered important factors for retention.

> *"The cost to the patient in time and effort to get to the surgery,…what time these appointments would be. How long they would take, how much effort it would take to get the patient… and also, if it's a research project, I always feel that patients shouldn't wait, …it's up to the nurse to make her appointments work so that she doesn't have anybody waiting there for any length of time,*
>
> ***Research nurse interview no 1***

Altruism

Many interviewees also perceived that where participants entered a trial for altruistic reasons they were more likely to remain in the trial.

> *"…They [participants] feel as though they want to do good because .. often a lot of them say "oh it's payback time", they think the NHS has been good to them so they want to give something back".*
>
> ***Trial manager interview 7***

Perceived benefit of trial participation

For peer review only - http://bmjopen.bmj.com/site/about/guidelines.xhtml

Most interviewees thought that if trial participants perceived an extra benefit from participating in a trial that this would contribute to retention particularly if they are being monitored for disease progression.

"In studies where (there) is … constant monitoring ……..they don't mind giving….. samples again and again as part of the trial….. But I …think …. if they can see some benefit then they'll do it but if they can't or if they've already got that benefit from giving the sample at the first time point they might not want to do it again and again"

Trial manager interview 10

Some of the benefits identified from participation were extra health monitoring for example: "*ECG*" "*BP*" and "*cholesterol*" as well as the extra attention from staff. However, these perceived benefits were thought to be trial dependent.

Age

Several age groups were highlighted as challenging for trial retention, for example, teenagers wishing to opt out of a trial when they reach the age of consent; working mothers juggling school runs with work time, and elderly participants who have either lost their independence or are involved with extended family caring activities. Young men with other interests were also thought to be challenging to retain in trials

"A young girl whose parents had agreed to be in the trial and then, you know, she didn't want to and then decided herself she was going to withdraw. And, you know, I can see the potential for that sort of thing if it's a trial in the sort of pre-teen, teen group that,… they might well change their mind….more interesting things going on in their lives, they've got more after school activities or they've got exams or they've got whatever. And coupled with being more independent and wanting to assert their own ideas".

Principal investigator interview 28

"In trials with older people, people will die, people will get frailer and maybe be institutionalised and not be able to continue or not want to continue for those sort of reasons".

Principal investigator interview 28

"There are all the usual things, we assume that older people don't move, there's a astonishingly high level of mobility in the older population. So lost to follow-up can occur just because people say "right, I'm going to go and live in…………..." or "I'm going to go and live near my daughter" or "I've off to ……….for the winter". [They]

For peer review only - http://bmjopen.bmj.com/site/about/guidelines.xhtml

do… a lot of child care, grandparental child care and sorting out their children's divorces and so on and so they get distracted and drop out completely through distraction".

Principal investigator interview 21

Other participant characteristics

Feelings of guilt and shame with not having achieved positive outcomes in a trial were thought to contribute to loss to follow-up in some disease areas / conditions e.g. in treatment for obesity trials, if participants were not losing weight they might not return to be weighed perhaps due to the shame and stigma associated with not achieving their expected aims. Retention in trials involving behaviour change was also thought to be problematic if change targets were not achieved by participants. For example, interviewees were generally in agreement that retention can be problematic in trials involving participants with a mental health issue.

"In nutrition studies we're very much bound by looking at healthy individuals to begin with. So from that perspective they may certainly be less motivated than an individual participating in a drug trial who has a severe or debilitating condition that, you know, they want to try and do something about".

Trial manager interview 15

"For smoking cessation or weight management or any of these things where certain behaviour is expected or wanting to be demonstrated. If they're not achieving it they are more likely to just drop out"

Research nurse interview 9

"Bi-polar groups, a lot of them find it very, very difficult going to an NHS facility".

Trial manager interview 17

"it depends on what area of medicine it is, for instance, if you're dealing with people with psychiatric illness, there's always a problem with attendance, whether that's within the surgery or whether that's within research".

Research nurse interview 1

The interviewees felt that participants with chronic diseases may have more interest in the treatment of their condition and return for follow-up visits. Others thought healthy volunteers may drop out of trials because of lack of interest in the trial.

2. Engaging with participants

For peer review only - http://bmjopen.bmj.com/site/about/guidelines.xhtml

Interviewees thought that engaging with the participants during trial recruitment and maintaining a good rapport was key to retention.

> *"It [the recruitment visit] sets the stage for the participants relationship with the trial …………. it's important to get that right, right from the start…. that affects whether people think it's worthwhile coming back…"*
>
> ***Trial manager interview 26***

> *"At the end of the day it's how the nurse and GP …….establish a rapport and a relationship with the participant,… that is the key issue in keeping participants in a study".*
>
> ***Research nurse interview 12***

> *"I think it is communication at all levels; so the nurse who interfaces with the trial management team behind the coordinating centre, … listening to the participant's views …and … feeding them back, ……. a two-way process. Because we're this side doing the research, we have to listen … so yes, … communication and understanding [the] population that you're working with".*
>
> ***Trial manager interview 13***

Some interviewees across all groups mentioned the impact of trial staff personalities on trial retention. Interviewees thought RNs needed to have a "*flexible approach*" and to "*be sympathetic*" toward participants, while TMs needed to be "*competent*", "*personable*", "*persistent*", "*enthusiastic*", and "*good communicators*".

> "*If you're enthusiastic, can give the information to the participants and…[are] sympathetic to …. queries, always being accommodating,[and] getting back to them if they have any queries …being absolutely spot on with, so that they don't feel they've been let down or you haven't been bothered to get back to them.*"
>
> ***Research nurse interview 5***

> "*I think there's a paradox here because they [trial managers] need to be obsessional about detail but they also need to be relaxed and flexible in their response to different situations ….and problems that come up. That's asking quite a lot, I think. So you know it's great if you find somebody who's good at it…..Or be very kind hearted. And prepared to work out of hours!*"
>
> ***Principal investigator interview 25***

3. Tailoring retention strategies

For peer review only - http://bmjopen.bmj.com/site/about/guidelines.xhtml

Tailoring specific retention strategies to enable different trial population groups to either return questionnaires or return to sites for follow-up was thought to improve retention. In mental health trials for example, some interviewees found that participants may wish to be followed-up at home rather than at a clinical site.

> *"Depending on the trial and the length of the trial and your client group …you would have a completely different strategy for… the fifteen to twenty-four year olds, to something… where you're looking at the seventy-five plus….. you do sort of tailor it [the strategy] to your group."*
>
> ***Research nurse interview 2***

4. Working environment

Some interviewees reported that the working environment at general practice trial sites might impact upon retention. It was thought to be important to have a staff member at the site to "*champion*" the study. Part-time RNs reported working in isolation and sometimes struggled to find a consulting room for follow-up visits which restricted the availability of flexible follow-up appointment times to offer to participants.

> *"The last study that I worked on, ..I struggled to find .. a room, the practice were incredibly accommodating, and really wanted me to have a room, but it just is very, very tight. …it has been, a major issue for me…It was affecting follow up for me because I was not always able to have a room when I was available, that ….would have been potentially available for participants, so it just narrowed down the opportunities for me to offer to participants to come in"*
>
> ***Research nurse interview 5***

> "*if somebody genuinely did want to make the trial work then it was possible to make it work. ………………..it needed somebody to actually manage the trial within that practice. The practices in which we had success I felt that we had a champion there who was fighting our corner for us"*
>
> ***Trial manager interview 27***

> "*the other thing is practice awareness, you know, because you said it's a big practice and if somebody comes in and hasn't got the sort of clock-in bit straight and they go to the desk and say, I've come to see X about this trial and, you know, new receptionist knows nothing. That can be quite off-putting so as long as everybody's engaged …and everybody knows about it".*
>
> ***Research nurse interview 2***

Discussion

Statement of principal findings

Based on in-depth interviews with primary care trial personnel we identified that incentive, communication and questionnaire strategies are used to try to increase retention in primary care trials. Participant characteristics e.g. interest in the trial, altruism and time, encourage retention. Interviewees thought that participant's perceptions of benefits from participation; the age of participants e.g. young men and teenagers; the disease area / condition e.g. mental illness and behavioural problems; and the research environment in which the trial was conducted e.g. availability of consulting rooms for follow-up appointments all contributed to loss to follow-up in trials.

Strengths and weaknesses

Research on strategies to improve retention of participants in primary care trials is limited and commonly grouped with recruitment strategies[14;15]. Trial retention is important because loss to follow-up can lead to incomplete data for the primary outcome, bias results and impact on the generalisability of trial findings. This study is the first to explore the use of retention strategies in a broad spectrum of randomised UK primary care trials and to identify factors associated with retention separate from recruitment strategies. Interviews were conducted with researchers experienced in design, leadership, management, and implementation of randomised UK primary care trials, many of whom reported that prior to this study that they had not thought deeply about loss to follow-up or strategies used to overcome this. With the focus by funders on recruitment rather than retention targets, this study specifically raises awareness of the complexity of retention in primary care trials and the many strategies and factors used to maximise retention. Although the thoughts and experiences of trial participants were not captured to further explore the barriers and facilitators to retention the results show that trial personnel have an understanding of the challenges participants encounter to stay in trial follow-up. It is unclear how generalisable the results are to randomised trials outside of primary care.

Meaning and implications for the use of retention strategies

For peer review only - http://bmjopen.bmj.com/site/about/guidelines.xhtml

These results enhance the interpretation and provide insight around the implementation of the results of our Cochrane review on ways to improve retention in trials[2]. Together, these two studies inform the future use of effective strategies to improve retention in primary care trials.

There was general agreement that small monetary incentives were likely to be viewed favourably by ethics committees and led to increased questionnaire response, in agreement with the findings of the Cochrane Review[2;2]. Although there was uncertainty among those interviewed about whether monetary incentives should be given up front or offered on receipt of data, the Cochrane review showed that either approach increased postal questionnaire response[2].

Scepticism among interviewees about non-monetary incentives and their value was also supported by the Cochrane Review which did not find them to be effective. Trialists may therefore need to reconsider the use of this type of incentive.

A range of different communication strategies were discussed by interviewees. Although trialists routinely preferred 1st class post, and the use of enhanced letters, the Cochrane Review found no evidence to support either approach. Therefore, 2nd class post and standard letters can probably be used for postal correspondence with primary care trial participants, with little impact on loss to follow-up.

Trialists had mixed opinions on the use of recorded delivery, nevertheless recorded delivery of questionnaires compared with a telephone reminder was found to be effective for improving questionnaire response[2;5]. To avoid any inconvenience associated with the use of this strategy, preplanning the delivery time with the participant with a reminder to expect a recorded delivery of trial materials is recommended if this strategy is to be used in future trials.

Different types of reminders and alternative questionnaires are routinely used by primary care researchers to improve trial follow-up, however there is no clear evidence that reminders and prompts in addition to the standard trial follow-up procedures are beneficial to retention or to improving questionnaire response[2]. Similarly, there is no evidence to suggest better response rates to shorter questionnaires[2].

For peer review only - http://bmjopen.bmj.com/site/about/guidelines.xhtml

Furthermore, interviewees were particularly negative toward the use of behavioural strategies and there was no effect found for these in our Cochrane review[2]. Case management, although seldom used, was thought to be potentially useful by interviewees, however, there is no evidence that this is effective[2]. The interviewees also thought that blinding participants to their allocated intervention would improve retention. This is in contrast to findings from the Cochrane review which found that an open trial design was more effective than a blind design in one trial[2;4]. Avenell argues that double blind trials do not reflect usual health care procedures, and that open trials, where the intervention is compared to no treatment or usual treatment, could give a better measure of the differential effects in normal care settings[4]. The open trial design, if used as a stand-alone strategy to improve retention, would need to be considered in the context of the overall aims and objectives of the trial. Further evaluation is needed in different trial contexts and settings if this retention strategy is to be adopted.

Several other factors and ways to improve retention were identified that were not covered in our Cochrane review[2]. Some of these such as relationships and communication between trial staff and trial participants are clearly more difficult to design interventions for and to evaluate. The factors identified indicate a need to train and support trial staff in ways to improve trial retention. It seems clear from this qualitative study that trial staff were oriented to participant concerns and needs (as they perceived them) and had a high level of empathy with participants.

Integrating qualitative research with Cochrane reviews

Integrating the results of the qualitative study and the Cochrane review helped us understand the different experiences primary care researchers have with the strategies identified by our Cochrane review[2]. The qualitative results support some of the results of our Cochrane review and demonstrate the potential transferability of the different effective retention strategies to trials conducted in primary care. For example incentives were employed in different ways to try to improve responses to postal questionnaires. The results have also helped identify other factors that might promote retention such as building a rapport with trial participants, and potential barriers like finding a consulting room for follow-up appointments. This mixed methods approach could be replicated in research contexts apart from primary care to determine how appropriate and useful the strategies identified by the Cochrane review[2] are in practice.

For peer review only

For peer review only - http://bmjopen.bmj.com/site/about/guidelines.xhtml

While the absolute effects of effective strategies identified by the Cochrane review are modest[2], this qualitative study highlights that multiple strategies are often used to try to achieve maximum retention in primary care trials. Furthermore it suggests that clinical trialists should consider other factors that may affect retention, such as building a rapport with participants and minimising the impact of trial follow-up on their daily lives. Consideration should also be given to the characteristics of the intervention, participants and trial personnel when choosing effective strategies identified by our Cochrane review to improve trial retention[2].

Future research

It was clear from the interviews that there is a move toward contacting participants by email and a desire to use text reminders when automated facilities for these become available for use in randomised trials. These new strategies would need evaluation in future trials.

Further research is also needed to identify potential barriers and facilitators to follow-up in trials e.g. with trial participants from different social, economic, age and disease groups. The results would be useful for primary care researchers and would help target and tailor strategies to meet the needs of different population groups to keep them specifically engaged in trial follow-up[16].

Conclusion

Qualitative research can help to interpret the results of systematic reviews by telling us what researchers do or think happens in research practice. These findings provide a deeper understanding of the factors that may facilitate retention such as rapport with participants and respect for their time, and highlights the participant and environmental characteristics that can limit the implementation of effective strategies. The use of small monetary incentives to improve questionnaire response is acceptable however a reconsideration of the use of non-monetary incentives, and certain communication strategies including 1st class post is needed. A similar exploration of barriers and facilitators to retention in other research contexts would help identify ways to improve retention in trials conducted in other research contexts.

For peer review only

Acknowledgements

We would like to thank all twenty nine interviewees who gave of their time and thoughts on the complexities of trial retention in primary care.

Statement of funding and competing interests

All authors have completed the ICMJE uniform disclosure form and declare no competing interests. This project was funded by the Medical Research Council Population Health Sciences Research Network grant number PHSRN 30

Contributorship Statement

Contributors VB, FS, GR, SM, SH and IN conceived the study idea. VB applied for ethics approval and collected the interview data.

VB, GR, FS and CV directed the analyses, which were carried out by VB. All authors participated in the discussion and interpretation of the results. VB wrote the initial manuscript draft. All authors critically revised the manuscript for intellectual content and approved the final version.

Data Sharing Statement

None

For peer review only

Reference List

(1) Janson SL, Alioto ME, Boushey HA. Attrition and Retention of Ethnically Diverse Subjects in a Multicenter Randomized Controlled Research Trial. *Control Clin Trials* 2001; 22(6, Supplement 1):S236-S243.

(2) Brueton VC, Tierney J, Stenning S, Nazareth I, Meredith S, Harding S et al. Strategies to improve retention in randomised trials . *Cochrane Methodology Group* 2013; in press.

(3) Sutherland HJ, Beaton M, Mazer R, Kriukov V, Boyd NF. A randomized trial of the total design method for the postal follow-up of women in a cancer prevention trial. *Eur J Cancer Prev* 1996; 5(3):165-168.

(4) Avenell A, Grant AM, McGee M, McPherson G, Campbell MK, McGee MA et al. The effects of an open design on trial participant recruitment, compliance and retention - a randomized controlled trial comparison with a blinded, placebo-controlled design. *Clinical Trials* 2004; 1(6):490-498.

(5) Tai SS, Nazareth I, Haines A, Jowett C. A randomized trial of the impact of telephone and recorded delivery reminders on the response rate to research questionnaires. *J Public Health* 1997; 19(2):219-221.

(6) McColl EM, Eccles MPM, Rousseau NSB, Steen INP, Parkin DWD, Grimshaw JMP. From the Generic to the Condition-specific?: Instrument Order Effects in Quality of Life Assessment. [Article]. *Med Care* 2003; 41(7):777-790.

(7) Cockayne S, Torgerson D. A randomised controlled trial to assess the effectiveness of offering study results as an incentive to increase response rates to postal questionnaires [ISRCTN26118436]. *BMC Medical Research Methodology* 2005; 5(1):34.

(8) Man MS, Tilbrook HE, Jayakody S, Hewitt CE, Cox H, Cross B et al. Electronic reminders did not improve postal questionnaire response rates or response times: a randomized controlled trial. *J Clin Epidemiol* 2011; 64(9):1001-1004.

(9) Dangour AD, Allen E, Elbourne D, Fasey N, Fletcher AE, Hardy P et al. Effect of 2-y n-3 long-chain polyunsaturated fatty acid supplementation on cognitive function in older people: a randomized, double-blind, controlled trial. *Am J Clin Nutr* 2010; 91(6):1725-1732.

(10) Williamson I, Benge S, Barton S, Petrou S, Letley L, Fasey N et al. Topical intranasal corticosteroids in 4-11 year old children with persistent bilateral otitis media with effusion in primary care: double blind randomised placebo controlled trial. *BMJ* 2009; 339(dec16_1):b4984.

(11) Trial Managers Network. 2013.
Ref Type: Online Source

For peer review only

(12) Pope C, Ziebland S, May N. Analysing qualitative data. In: Catherine Pope, Nicolas May, editors. Qualitative Research in Health Care. second ed. London: BMJ Publishing Group; 2000. 75-88.

(13) Farmer T, Robinson K, Elliott SJ, Eyles J. Developing and Implementing a Triangulation Protocol for Qualitative Health Research. *Qual Health Res* 2006; 16(3):377-394.

(14) Leathem C, Cupples M, Byrne M, O'Malley M, Houlihan A, Murphy A et al. Identifying strategies to maximise recruitment and retention of practices and patients in a multicentre randomised controlled trial of an intervention to optimise secondary prevention for coronary heart disease in primary care. *BMC Medical Research Methodology* 2009; 9(1):40.

(15) Graffy J, Grant J, Boase S, Ward E, Wallace P, Miller J et al. UK research staff perspectives on improving recruitment and retention to primary care research; nominal group exercise. *Fam Pract* 2009; 26(1):48-55.

(16) Kimmel SE, Troxel AB. Novel incentive-based approaches to adherence. *Clin Trials* 2012; 9(6):689-695.

For peer review only

Use of strategies to improve retention in primary care randomised trials: a qualitative study with in-depth interviews

Brueton V

Research Scientist, Meta-analysis Group
MRC Clinical Trials Unit at UCL
Aviation House
125 Kingsway
London WC2B 6NH
e-mail: vcb@ctu.mrc.ac.uk

Stevenson F

Senior Lecturer
UCL Research Department of Primary care and Population Health
Royal Free and University College Medical School
Rowland Hill Street
London
NW3 2PF

Vale CL

Senior Scientist Meta-analysis Group
MRC Clinical Trials Unit at UCL
Aviation House
125 Kingsway
London WC2B 6NH

Stenning SP

Senior Statistician
MRC Clinical Trials Unit at UCL
Aviation House
125 Kingsway
London WC2B 6NH

Tierney JF

Senior Scientist Meta-analysis Group
MRC Clinical Trials Unit at UCL
Aviation House
125 Kingsway
London WC2B 6NH

Harding S

Program leader: Ethnicity and Health

For peer review only

BMJ Open qualitative paper submitted Version 3 VB 26.11.13

MRC Social & Public Health Sciences Unit
University of Glasgow
4 Lilybank Gardens
Glasgow G12 8RZ

Nazareth I

Professor of Primary Care
PRIMENT Clinical Trials Unit
Research Department of Primary care and Population Health
UCL Medical School
Rowland Hill Street
London
NW3 2PF

Meredith S

Deputy Director
MRC Clinical Trials Unit at UCL
Aviation House
125 Kingsway
London WC2B 6NH

Rait G

Clinical Senior Lecturer
PRIMENT Clinical Trials Unit
Research Department of Primary care and Population Health
UCL Medical School
Rowland Hill Street
London
NW3 2PF

For peer review only

For peer review only - http://bmjopen.bmj.com/site/about/guidelines.xhtml

BMJ Open qualitative paper submitted Version 3 VB 26.11.13

Abstract

Objective

To explore strategies used to improve retention ~~identified by a Cochrane review,~~ in ~~UK~~ primary care randomised ~~controlled~~ trials.

Design

Qualitative in-depth interviews and thematic analysis.

Participants

Twenty nine UK primary care chief and principal investigators, trial managers and research nurses.

Methods

In-depth face to face interviews.

Results

Primary care researchers ~~described using various incentives~~use incentive and communication strategies to improve retention in trials, but were unsure of their effect. Small monetary incentives were used ~~in an attempt~~ to increase response to postal questionnaires. Non-monetary incentives were used although there was scepticism about the impact of these on retention. Nurses routinely used telephone communication to encourage participants to return for trial follow-up. Trial managers ~~routinely~~ used 1st class post~~, to try to increase questionnaire response.~~ ~~Shorter~~shorter questionnaires and improved questionnaire designs with the aim of improving questionnaire response ~~were sent to non-responders, and suggestions offered for improving questionnaire design.~~. Interviewees thought ~~that~~ an open trial design could lead to biased results and were ~~generally~~ negative about using~~the use of~~ behavioural strategies to improve retention.

There was consensus among the interviewees that effective communication and rapport with participants, participant altruism, respect for participant's time, flexibility of trial personnel and appointment schedules, and trial information improve retention. Interviewees noted particular challenges with retention in ~~teenagers, disease type~~ mental health trials and those

Formatted: Not Highlight

Formatted: Not Highlight

Formatted: Not Highlight

Formatted: Not Highlight

For peer review only

For peer review only - http://bmjopen.bmj.com/site/about/guidelines.xhtml

involving teenagers ~~and the work environment at trial sites were thought to contribute to loss to follow up~~.

Conclusions

The findings of this qualitative study ~~Integration of the findings of this qualitative study with the results of the Cochrane review in depth exploration of the applicability of the Cochrane review~~ have allowed us to reflect on research practice around retention and highlight a gap between such practice and current evidence. Interviewees describe acting from experience without evidence from the literature, which supports ~~The~~the use of small monetary incentives to improve questionnaire response ~~is effective and acceptable.~~. No such evidence exists for non-monetary incentives or first class post, use of which may need ~~A~~ reconsideration ~~of the use of non-monetary incentives, and some communication strategies is needed. A similar~~. An exploration of barriers and facilitators to retention in other research contexts ~~–~~ may be justified~~would help identify ways to improve retention in trials conducted in other research contexts~~.

Formatted: Not Highlight

Formatted: Not Highlight

Formatted: Not Highlight

Formatted: Not Highlight

Formatted: Not Highlight

Formatted: Not Highlight

For peer review only

BMJ Open qualitative paper submitted Version 3 VB 26.11.13

Introduction

Retention in primary care randomised controlled ~~trials (RCTs)~~trials ~~-~~conducted across different disease areas and communities can be challenging. Inadequate retention can reduce the power of a trial and introduce bias, particularly if drop-out differs across trial arms. Reasons for loss to follow-up can include a change in the participants' location, withdrawal from treatment and / or loss of commitment to the trial, for example due to complicated treatment regimens[1]. A Cochrane review of strategies to improve retention in trials demonstrated~~12. A Cochrane review of strategies to improve retention in RCTss demonstrated~~ that adding a monetary incentive and offering higher valued monetary incentives increased postal and electronic questionnaire response[2] ~~,21.~~ Questionnaire response was also increased by recorded delivery of questionnaires, a "package" of postal communication strategies known as the Total Design Method ~~and a~~ (TDM~~)~~)[3] and an open trial design[4], although these were based on the results of single ~~RCTs~~ trials. The evidence of an effect for shorter questionnaires and questionnaires relevant to the disease / condition was less clear. Also, there was no good evidence that the following strategies improved retention: adding or offering a non-monetary incentive, communication strategies (including use of first class mail), behavioural motivational strategies, new questionnaire formats and case management.

~~Primary care RCTs often require participants to return to sites for clinical outcome measurement for example, assessment of disease progression[3;43,4]. However, in the Cochrane review, there were few evaluations of strategies to increase this type of retention, and only~~Only six of the 38 retention ~~RCTs~~trials included in the Cochrane review were embedded in UK primary care trials~~.~~ and all ~~Rather the included retention RCTs~~ targeted improving response to postal questionnaires in order to improve retention[5-8] ~~5-8~~ ~~.~~. However, primary care trials also require participants to return to sites for clinical outcome measurement to assess disease progression[9;10]. There could be important factors that contribute to the use of strategies to improve questionnaire response and for participants to return to sites for follow-up in the context of primary care trials. ~~Moreover, there may be barriers to using strategies to improve retention and in particular those identified by the Cochrane review.~~

We conducted ~~-~~a qualitative study with ~~staff involved in~~ primary care ~~RCTs~~trial personnel to explore the ~~use of of~~ strategies ~~strategies,~~ generally used to improve retention in primary

Formatted: Do not check spelling or grammar

Field Code Changed

Field Code Changed

Field Code Changed

Field Code Changed

Field Code Changed

Field Code Changed

Field Code Changed

Field Code Changed

Field Code Changed

Formatted: Not Highlight

Formatted: Not Highlight

Formatted: Not Highlight

Formatted: Not Highlight

For peer review only

For peer review only - http://bmjopen.bmj.com/site/about/guidelines.xhtml

BMJ Open qualitative paper submitted Version 3 VB 26.11.13

care randomised trials and to map these to the results of our Cochrane review[2] ~~including those identified by a Cochrane review.~~ , ~~to improve RCTs . The aim was to explore the acceptability, feasibility and use of the strategies identified by the Cochrane review, in primary care RCTs. A secondary aim was to identify factors thought to contribute to retention in the context of primary care RCTs.~~

Methods

Sampling the study population

To explore the use of strategies to improve retention in randomised primary care ~~randomised~~ trials~~RCTs~~, in-depth face to face interviews were conducted with principal / chief investigators (PIs), trial managers (TMs) and research nurses (RNs) purposively sampled from a sampling frame of primary care randomised trials~~RCTs~~ ~~. A sampling frame was established using primary care RCTs~~ published from 2000- - 2010. The trials ~~that~~ were identified from either the MRC General Practice Research Framework (GPRF) database of clinical research projects, websites of UK primary care research units or from hand searches of trials~~RCTs~~ published between 2009 - 2010 in *Lancet, British Medical Journal, Family Practice* and *British Journal of General Practice*. The trial~~RCT~~ publications identified ~~spannned~~spanned different disease areas, research units, and levels of ~~-~~loss to follow-up (Tables 1 and 2) and were stratified by ~~loss to follow-up~~ levels~~of~~ of loss to follow-up (≤~~<~~20%, ≥20%), and ~~the date of~~ publication date (2000 - 2004 and~~,~~ 2005 - 2010). All PIs, TMs and RNs were identified for recruitment ~~to the qualitative study~~ from the list of authors associated with each trial~~RCT publication~~ in the sampling frame ~~. TMs and RNs listed as co-authors were also identified in this way. TMs and RNs not listed as co-authors were identified for recruitment~~ or through ~~records held by the MRC GPRF~~ records ~~-~~ of trial staff associated with MRC GPRF primary care trial~~RCT~~ publications. In addition ~~we recruited,~~ -TMs were identified through the Trial Managers Network[11] ~~.9.~~ TMs recruited in this way had to be associated with a randomised primary care ~~- RCT~~ trial published ~~-~~between 2000 - 2010 which~~that~~ was subsequently entered into the sampling frame.

Data collection

~~Ethics approval was granted by University College London Research Ethics committee (application no 2342/002). Potential interviewees were sent an information sheet and recruited via email. Participants were given information and asked to sign a consent formFace-to-face~~

6

Field Code Changed

Formatted: Not Highlight

Formatted: Not Highlight

Formatted: Not Highlight

Field Code Changed

Field Code Changed

Formatted: Check spelling and grammar, Not Superscript/ Subscript

Formatted: Not Highlight

For peer review only

BMJ Open qualitative paper submitted Version 3 VB 26.11.13

interviews explored the use, of strategies to improve retention and the use of the six types of strategies identified by the Cochrane review in primary care RCTs (Interview schedule Appendix 1). Initially interviewees were asked about the factors that they thought contributed to retention in primary careRCTs. (i.e. incentives, communication methods, new questionnaire formats, behavioural, case management and methodological strategies[2]) spontaneously by, theyThey were then asked specifically about them. six types of strategies identified were: incentives, different means of communication, new questionnaire formats, behavioural, case management and methodological strategies[21]. (Interview schedule Appendix 1) Ethics approval was granted by University College London Research Ethics committee (application no 2342/002). Potential interviewees were sent an information sheet and recruited via email. Prior to the interview participants were given study information and asked to sign a consent form. Face-to-face interviews explored everyday retention practices and the factors thought to contribute to retention in primary care trials. If retention strategies found by the Cochrane review[2] (i.e. incentives, communication methods, new questionnaire formats, behavioural, case management and methodological strategies) were not mentioned spontaneously by interviewees, they were asked specifically about these. (Interview schedule Appendix 1). Interviewees were also asked specifically about the role of the ethics approval process on the use of monetary incentives to improve retention in primary care trials.

The interviewees were asked to consider RCTs generally when answering questions rather than focusing solely on the trial that they were sampled from. Interviewees were also asked specifically about the role of the ethics approval process on the use of monetary incentives to improve retention in primary care RCTs. The interviews were approximately one hour long, were conducted by one author (VB) in a place convenient to the interviewee, and they were recorded digitally and took approximately one hour. The interviews were developed iteratively. Transcripts were checked following each interview during data collection for flow and usefulness of results and the interview schedule modified accordingly. Appropriate probes were used to allow participants to expand on issues around the use of strategies to improve retention.

Data management and analysis

Formatted: Not Highlight

Field Code Changed

Formatted: Not Highlight

Formatted: Not Highlight

Formatted: Not Highlight

Formatted: Not Highlight

Formatted: Not Highlight

For peer review only - http://bmjopen.bmj.com/site/about/guidelines.xhtml

BMJ Open qualitative paper submitted Version 3 VB 26.11.13

We conducted a thematic content analysis. All transcripts were reviewed and validated by at least two authors (CV, FS, GR, VB). They for the interview technique to: a) confirm that the interviewees understood and were able to answer the questions asked during interview b) that leading questions were avoided and c) that appropriate probes were used to allow the participants to expand on issues around the use of strategies to improve retention. The textual data were managed using Atlas ti 6. A transcript review group was formed with four authors (VB, CV, GR, FS),) and —was heterogeneous in terms of the members' professional background. Each transcript was reviewed independently by at least two group members of the transcript review group who documented the . Eemergingemerging major themes were documented independently by each member andwhichh were then discussed by the group in pre-planned meetings. This group met monthly while data were collected. There was a high degree of convergence in the themes identified-.

Data were also analysed iteratively so early results could be incorporated and probed in later interviews to increase the depth of the findings. Labels were agreed and used as broad codes to label textual data associated with major themes. Sub codes were identified discussed and agreed between two authors (FS, VB) and also used to label textual data. Transcripts were subsequently coded by one author (VB). The first two transcripts were checked by another author (FS). A key consideration in coding was to mark data produced spontaneously and those which were specifically asked about, therefore data were coded to take account of the response and the question that prompted that response in order to apply appropriate emphasis on responses. Codes for the six strategies identified by the Cochrane review i.e. *"communication"*, *"incentives"*, *"questionnaires"*, *" methodology"*, *"case management"* and *"behavioural"* were used as a priori codes for the later part of the interview transcripts as participants were asked specifically about the use of Cochrane review strategies.

Textual data in each transcript relating to retention strategies wwereas subsequently retrieved from athe coded database and . Coded data were constantly compared within and across -the three groups of trial personnel (i.e. PIs, TMs, RNs) and within the three transcript groups to ; a)to –identify the use of each strategy and b) to document the reported advantages and disadvantages of using each strategy. Any factors thought by the interviewees to contribute to either retention or loss to follow-up were also coded and retrieved for analysis[12;13] [10;1110,11]. Relevant quotes, representing the interviewee's views, were selected to illustrate the results. Interviews were conducted between August 2010 and May 2011 until no new content or

Formatted: Not Highlight
Formatted: Font color: Auto
Formatted: Not Highlight
Formatted: Font color: Auto
Formatted: Font color: Auto
Formatted: Font color: Auto
Formatted: Font color: Auto
Formatted: Not Highlight
Formatted: Not Highlight
Formatted: Not Highlight
Field Code Changed
Field Code Changed

For peer review only - http://bmjopen.bmj.com/site/about/guidelines.xhtml

~~themes emerged.~~ Relevant quotes, representing the interviewee's views, were selected to illustrate the results. Interviews were conducted between August 2010 and May 2011 until no new content or themes emerged.

Results

Fifty four trial personnel were identified for recruitment from 37 randomised primary care trials from England and Scotland ~~that were~~ included in the sampling frame. ~~Ten PIs, ten TMs and nine RNs were interviewed across 23 of the 37 primary care RCTs identified for the sampling frame. tRCTs from England and Scotland were identified through the sources searched.~~ Eleven of the fifty four invitees declined to participate (PIs (n=10), TMs (n=1~~)~~)). Seven of these PIs ~~who declined~~ recommended another ~~named~~ co-author to invite ~~for interview.~~. Fourteen of the fifty four ~~invited~~ did not respond (PIs (n=10~~9~~10), TMs (n=3), RNs (n=1~~)~~). Ten PIs, ten TMs and nine RNs were recruited from 23 of the 37 primary care trials identified.

Formatted: Not Highlight

Formatted: Not Highlight

Interviewees were involved in running ~~were sampled from primary care~~ randomised primary care trials~~RCTs~~ ~~–~~conducted in ~~the fields of~~ nutrition, health promotion, neurology, gynaecology, mental health, musculoskeletal, ear nose and throat (ENT), respiratory, ~~and~~ endocrine medicine and ~~, as well as from RCTs of~~ minor medical conditions. Collectively their experiences, which were not limited to the trial from which they were sampled, included trial design, implementation, coordination and data collection at GP practice sites by post, face to face and electronic methods. Trial~~RCT~~ publications reported loss to follow-up rates between 5% - 39%. Twenty interviewees were from trials~~RCTs~~ with ≤~~<~~20% loss to follow-up reported~~.~~ (See Tables 1 and 2). Different types of follow-up were used in these trials: face-to-face follow-up at clinics (n=20), postal questionnaires (n~~=~~=6), follow-up at home (n=2) or self-completion diaries (n=2). Combinations of follow-up types were also used: clinic visits or postal follow-up (n= 4), telephone and postal follow-up (n=2), registry data and follow-up at home (n=1).

Formatted: Not Highlight

The PIs were responsible for the design and implementation of trials~~RCTs~~, including research governance and ethics approval applications. Nine PIs were GPs with academic roles, and one was a non-clinical senior academic. The TMs were based in academic research units and~~.~~ ~~They~~ managed trial data collection via telephone, post, email and SMS text message.~~;~~ TMs also~~they~~ coordinated and monitored data centrally and communicated with trial sites. Six RNs

For peer review only - http://bmjopen.bmj.com/site/about/guidelines.xhtml

BMJ Open qualitative paper submitted Version 3 VB 26.11.13

~~were based at GP practice sites where they~~ coordinated clinical research at GP practice sites. They collected biomedical and clinical data by telephone and face-to-face interview, and ~~also~~ communicated with the coordinating centre and with PI clinicians at sites. Three RNs were based at a primary care research network coordinating centre where they answered clinical data collection queries and monitored data quality. ~~See Tables 1 and 2 for the characteristics of the study sample.~~

~~Interview results~~Results are presented first on the interviewee's experiences of and perspectives on ~~–~~using communication, incentive and new questionnaire strategies, identified by the Cochrane review[2], to improve primary care trial~~RCT~~ retention. This is followed ~~e~~by views on ~~–~~ other~~,~~ less frequently evaluated~~,~~ strategies identified by the Cochrane review[2] i.e. behavioural, case management, and methodological strategies~~ are then presented~~.~~, followed by the results on the factors~~ Factors thought to contribute to retention and loss to follow-up in primary care trials~~RCTst~~ are also presented.

Formatted: None

Field Code Changed

Formatted: Do not check spelling or grammar

Field Code Changed

Formatted: Do not check spelling or grammar

Formatted: None

The use of retention strategies identified by the Cochrane review

1. Use of ~~Incentives~~incentives

~~Use of monetary~~Monetary incentives ~~strategies~~

Monetary incentives and offers of monetary incentives were mentioned spontaneously by most PIs and TMs as useful strategies to improve response to postal questionnaires. Over half of the PIs interviewed had used monetary incentives given in either cash or voucher format to increase questionnaire return, ~~even~~ although there was uncertainty about their effect. The different types used were~~:~~ high street vouchers and mobile phone vouchers, but vouchers were thought of as a burden to administer for trial management teams. ~~The more generic~~ Generic vouchers were thought to be more acceptable to all trial participant groups. It was felt that the incentives should be seen by trial participants as a "*thank you*" rather than as payment for participation.

The monetary value of the incentives used was between £5 and £20 and this amount was considered reasonable for adults and children~~ participants.~~. Larger monetary incentives (>£50) were perceived as coercion or bribery by interviewees across the three groups

For peer review only - http://bmjopen.bmj.com/site/about/guidelines.xhtml

interviewed. Offers of entry into a prize draw, although seldom used as a strategy to improve questionnaire response, were thought by some interviewees to be a cost effective alternative to giving a monetary incentive.

~~MThe monetary incentives used in primary care RCTs were mostly given in different voucher formats rather than cash,. and They were used to; a) increase questionnaire return orr at sites, b) given on behalf of the trial team, as a thank you to participants. The different types used were: high street vouchers and mobile phone vouchers, but vouchers were thought of as a burden to administer for trial management teams. The more generic vouchers were thought to be more acceptable to all trial participant groups.~~

There was uncertainty among TMs about -effective ways to administer -incentives during follow-up ~~in order~~ to optimise questionnaire response ~~for the primary outcome. Some trial managers were involved in RCTs that~~. Some, -gave a small incentive at different follow-up time points e.g. £5 at randomisation and £5 at trial completion. Others sent an~~the~~ incentive with the questionnaire, or on receipt of a completed questionnaire.

TMs and PIs sampled from trials with ≤20% loss to follow-up appeared to use more strategies to improve retention than those from trials with ≥20% loss to follow-up. For example those in the ≤20% group gave -incentives at different time points to try to keep the participants motivated to return questionnaires. TMs and PIs sampled from trials with ≥20% loss to follow-up seemed more cautious about the use of incentives and some felt participants could feel coerced if incentives were used to improve follow-up.

~~Use of non~~Non-monetary incentives

Non-monetary incentive strategies or gifts i.e.~~such as;~~ pens, certificates of appreciation, key rings and mugs were sometimes used in an attempt to improve both participant recruitment and retention. These were used to market the trial, and in the case of pens, to encourage and remind participants to return completed questionnaires. Interviewees thought that these gifts could be perceived by trial participants as patronising, not useful, or associated with charity fundraising and also as a waste of public money, especially if the participant had altruistic motivations for ~~taking part in a trial~~participation.

Formatted: Not Highlight

Formatted: Not Highlight

Formatted: Not Highlight

For peer review only - http://bmjopen.bmj.com/site/about/guidelines.xhtml

"I think people are very sensitive about money especially in the public sector, ...if they see something that could be considered wasteful or extravagant, I don't think it would go down too well. Because...... if you get involved in a study that's being funded from public funds and from charity, you know, it's quite an altruistic thing to do, I don't think people are looking to be rewarded for it ....then they see you wasting money on pens and mugs or, I don't think it would go down very well".

Trial manager interview 7

Formatted: Not Highlight

~~Few interviewees thought that the gifts given were practical and useful to participants.~~ Some interviewees thought that the money used to finance such non-monetary / gift incentives could be better used to subsidise follow-up visits for participants. Indeed there was consensus amongst interviewees about the importance of reimbursing ~~participants'~~ travel and parking expenses associated with ~~such~~ follow-up visits. Some interviewees differentiated this from giving incentives to participants. Others recounted cases where participants did not want to be reimbursed particularly if they ~~it~~had ~~motivation for taking part was~~ altruistic motivations for participation, and others felt that ~~told of how~~ an offer of money to cover expenses could make participants feel valued. ~~, and there~~ There was a general feeling that this varied by participants' socio-economic circumstances e.g. it was thought that participants from higher socioeconomic groups were less likely to accept a monetary incentive.

"We actually gave them ten pounds for their travel costs, once at the beginning and once at the end.. and a lot of them didn't want it, you know, they'd say, oh... And I'd say, well just pop it in your local charity".

Research nurse interview 2

Ethical approval for the use of incentives

Interviewees across all groups thought it was important that ethics committees sought justification for ~~asked questions about~~ the use of incentives in trials~~RCTs~~. They felt that ~~ethics committees still varied in their~~ opinion varied among ethics committees about the use of incentives to improve ~~response or~~ retention but ~~in RCTs. Overall , the interviewees thought that ethics committees were~~overall – they were now more accepting of the use of small monetary incentives and of reimbursing participants' trial related expenses. Some PIs said that it was helpful to know who was on the ethics committee and that approval to use incentives is generally granted when a clear justification for ~~the~~ use ~~of incentives~~ is given. Others thought ~~the use of~~ incentives ~~was~~were likely to be approved if ~~the financial incentive involved paying~~they were used to pay participant~~the~~ travel expenses~~ of the participants~~.

For peer review only - http://bmjopen.bmj.com/site/about/guidelines.xhtml

BMJ Open qualitative paper submitted Version 3 VB 26.11.13

2. Use of communication strategies

Communication strategies, for example contact with participants by telephone, letter and email during follow-up, were spontaneously mentioned and routinely used to improve trial ~~RCT~~ retention. RNs and TMs used these as first line retention strategies.

~~Use of telephone~~Telephone calls ~~to participants~~

RNs frequently used telephone calls, to arrange or re schedule trial~~RCT~~ follow-up appointments or to remind participants to return postal questionnaires. Both RNs and PIs were conscious not to harass participants with too many telephone reminders. TMs thought reminder calls to participants prior to follow-up appointments might increase follow-up attendance~~ ~~. A telephone reminder to a site to remind the RN that a participant is due for a follow-up appointment was also thought to potentially improve retention. PIs did not contact participants by telephone and one thought it would be "*daunting"* for a participant to receive a call from a PI. Nevertheless, PIs were involved in decisions about when to use telephone contact, especially when they thought that participants were fatigued by providing data in diaries or repeat questionnaires.

~~Use of letters to trial participants~~Letters

Letters were used predominantly by TMs to communicate with trial participants, but there was uncertainty about their effect. Electronic coloured signatures, franked institutional logos placed on outward envelopes, different coloured envelopes, and first class outward post were used to attract the attention of participants.

Letters~~, when used,~~ were usually signed by TMs rather than PIs~~; as~~, the TMs thought that consistency in the signatory was more important than the status of the signatory for improving ~~retention and~~ questionnaire response. Some TMs sent letters to participants in time for weekend delivery, but were unsure whether this ~~influenceded~~influenced response. PIs thought that letters that were personalised, and the tone of the letter were important for retention or improving questionnaire return, but that the associated large mail-outs were burdensome for trial staff. Greeting cards were used occasionally to maintain communication with participants~~, in the hope of increasing retention. However;~~ however, interviewees were negative about the impact of these on retention and questionnaire response, with the exception

Formatted: Not Highlight

For peer review only - http://bmjopen.bmj.com/site/about/guidelines.xhtml

BMJ Open qualitative paper submitted Version 3 VB 26.11.13

of the use of birthday cards in paediatric trials~~RCTs~~, and ~~also~~ Christmas cards sent to sites from coordinating centres.

> *"I'm routinely suggesting that we send out birthday cards to children. We've done Christmas cards to participants on behalf of the trial team but there's no evidence that it works, but I think that it's a gesture and it's in the right direction, it's better than nothing".*
>
> ***Trial manager interview 27***

There was uncertainty ~~among the interviewees~~ about the effect on retention / response of the different types of postal delivery ~~systems~~ they use to send trial materials to participants. First class post was used to send trial materials to participants and 2nd class post for questionnaire return. While some TMs and RNs thought that 1st class post was a waste of financial resources, other TMs thought 1st class stamped letters were more likely to be opened by participants. Recorded delivery was occasionally used to send important study-related materials, or to resend questionnaires to non-responders. Some ~~interviewees~~ thought recorded delivery~~this~~ was useful to find out if a participant had changed address, but others thought recorded delivery ~~was~~ could be inconvenient~~,~~ e.g. ~~for the participant~~ if the participant ~~y~~ had to collect trial supplies from a post office. ~~First class post was used to send trial materials to participants and 2nd class post for questionnaire return. While some TMs and RNs thought that 1st class post was a waste of financial resources, other TMs thought 1st class stamped letters were more likely to be opened by participants.~~

Some TMs noticed that retention improved when questionnaires were sent before clinic visits as this enabled participants to complete the questionnaire at home beforehand. A prepaid envelope for return of postal questionnaires was thought to reduce the cost of postage to participants and the inconvenience of having to buy a stamp. To minimise respondent burden, clear instructions on how to return the questionnaire once it is completed was thought by TMs to contribute to improved postal questionnaire response.

~~Use of~~ E~~electronic contact with trial participants~~lectronic communication

TMs and RNs used emails to ~~-~~contact ~~with~~ participants who preferred this approach. SMS text messaging was used less so~~, but was thought to be potentially useful for retention. There were mixed views on the effect of SMS texting on retention in RCTs.~~ but ~~Most~~most interviewees thought that it would be useful for communicating with young people. The use of an

For peer review only - http://bmjopen.bmj.com/site/about/guidelines.xhtml

BMJ Open qualitative paper submitted Version 3 VB 26.11.13

automated system for texting appointment reminders similar to that used by the NHS for clinic appointments was thought to be a potentially useful way to improve trial retention. However one RN reported that the reminders sent via the system did not improve clinic attendance.

~~Use of newsletters in RCTs~~ Newsletters

Most interviewees thought newsletters were useful for keeping participants and site clinicians informed about trial recruitment, retention, and general trial news ~~about the trial.~~. Others thought ~~they~~these were less useful and could bias trial results by contaminating and confounding the results of the *treatment as usual* group. The frequency with which newsletters were sent to participants~~,~~ varied from fortnightly to annually, and depended on the condition /~~or~~ disease, intervention, and length of follow-up. ~~TMs and RNs were less convinced of their usefulnessto participants. However some~~Some PIs thought~~mentioned that~~ having trial-specific information and answers to frequently asked questions as a resource on a website could benefit retention.

3. Use of new questionnaire formats ~~strategies~~

None of the interviewees spontaneously mentioned ~~using modifications to~~ modifying a follow-up ~~questionnaires~~ questionnaire to improve response or retention, although some PIs and TMs ~~did use~~ had used shorter questionnaires for non-responders. When asked specifically about the impact ~~effect~~ of questionnaire length on retention, interviewees expressed a preference for shorter questionnaires, which they ~~were~~ thought ~~to~~ increased response. Interviewees also thought that long questionnaires administered frequently led to "*questionnaire fatigue*", and some thought that dividing long questionnaires into several short questionnaires could be useful to reduce the burden of data collection on participants. However, some PIs thought that if the questionnaire content was of interest to the ~~participants~~ participant, then the length of the questionnaire may not affect response. TMs and PIs thought the involvement of ~~mentioned that involving~~ patient representatives in the design and pre-test of questionnaires could improve retention. Many suggestions were also offered for improving questionnaire design in order to increase questionnaire response (Figure 1).

How ~~the~~a questionnaire was administered and by whom was also thought to ~~to also~~ affect ~~impact on~~ questionnaire response and retention~~retention~~. Some PIs and TMs thought that if

Formatted: Not Highlight

For peer review only

data were collected at ~~the questionnaire is administered at the~~ the clinic site by a nurse rather ~~than sent~~than by post that ~~that~~ this could increase response~~:~~.

4. Use of other strategies to improve retention

Other strategies identified by the Cochrane review[2] were not routinely used or mentioned spontaneously by interviewees as ways to improve retention. Opinions were mixed about the usefulness of ~~These were: a)~~ a behavioural strategy where participants are given information about goal setting and time management to facilitate successful trial completion; ~~b)~~ methodological strategies where a blind trial design was compared to an open / unblind trial design; and~~c)~~ case management where trial assistants manage participant follow-up, by arranging services to enable participants to keep trial follow-up appointments. ~~Opinions were mixed about the usefulness of such strategies as ways to improve retention in primary care RCTs.~~

Behavioural strategies were described to interviewees as *"Arranging workshops to give participants information about goal setting and time management"* for example in the context of~~–~~ an exercise behaviour change ~~parenting and exercise RCTs.~~intervention. When asked specifically about the use of such a ~~these strategies~~ strategy, interviewees were generally negative. Such strategies were discussed in the following ways: "*strange*", "*preachy",* "*condescending*", "*never came across it",* "*would never think of doing it*", *I'd be surprised if it worked*" and "*would need evidence that it worked*". Some thought that a behaviour~~behavioural~~ change retention strategy could~~strategies should~~ not be used ~~only be used as part of a trial intervention rather than as strategies to improve retention, in RCTs and they felt that type of~~ because it may ~~strategy would~~ contaminate a behaviour change trial ~~the~~ intervention and ~~and could~~therefore may affect the generalisability of trial results.

Although the interviewees were familiar with the concept of blind and open ~~RCTs~~trials, they were not aware of the use of either design as a strategy to improve trial retention. Interviewees felt that using an open trial design to improve retention could bias ~~trial~~ results, particularly as blinding is used to reduce bias in trials ~~and also that participants may drop out of a RCT if they are not allocated to their preferred intervention.~~. They also felt that ~~some interventions, for example therapist-led behavioural interventions, cannot be evaluated using~~ a blind trial design~~RCT designs~~ could not be applied to therapist-led behavioural interventions~~.~~

Formatted: Not Highlight

Formatted: Do not check spelling or grammar, Superscript

Formatted: Not Highlight

Formatted: Not Highlight

Formatted: Not Highlight

Formatted: Not Highlight

Formatted

Formatted: Not Highlight

Formatted: Not Highlight

Formatted

Formatted: Not Highlight

Formatted

For peer review only - http://bmjopen.bmj.com/site/about/guidelines.xhtml

One TM had used case management to improve trial retention. This involved the TM arranging services to enable participants to attend trial follow-up appointments. The interviewees generally thought that case management could improve trial retention with some groups e.g. the elderly, disabled and participants with low incomes but they also thought this could create dependence on the service. The impact of this strategy on retention was thought by some to depend on the personality of the case manager.

~~Other factors~~Factors thought to influence~~contribute to~~ trial retention ~~in primary care RCTs~~

In addition to the strategies employed to improve retention, interviewees also mentioned other factors that had both positive and negative influence on retention:

~~Use of participant's time~~

1. Participants

A key factor thought to optimise ~~contribute to~~ retention was to ~~reducing~~reduce the burden of follow-up for participants in terms of the length of time they spend in follow-up activities~~,~~ and ~~also~~ the type of data collected (e.g. ~~biomedical specimens,~~ long questionnaires, biomedical specimens~~), by using more~~). More convenient ways to collect outcome data, e.g. ~~such as~~ online data collection ~~bases~~ were thought to be more effective for retention. ~~.~~ Reducing the burden of follow-up visits by streamlining appointments or finding more convenient ways to collect key data (e.g. on line or by telephone) were also considered important for retention.

> *"The burden of activity on the patients, how easy it is for them, how long it's going to take, how intrusive in terms of blood samples or answering huge long questionnaires, it's partly the complexity as well, if it's very simple for them to do, often you just want very simple data back from people, alive or dead, or specific complications, if it can be done either with a, click of a button by sending a text or an email to people or just …~~a very you know,~~ reply paid cards so you're just getting the data back from them, ..~~I suppose~~ the other thing is~~….~~…~~trying~~…… to do electronic follow-up as much as possible so you don't actually have to bother the patient, you have their consent to access their electronic records and either their Primary care data can be interrogated ~~…a way of linking all of those together, like with Cancer Registry, Registrar General data, so I suppose the more you can automate it the easier it becomes".~~…".*
>
> ***Principal investigator interview 4***

The time participants spend getting to, ~~and~~ from, and at follow-up visits, giving participants adequate time at follow-up visits, and ensuring that participants were not kept waiting too long for follow-up appointments were all considered important factors for retention.

Formatted: Font: Bold

Formatted: Not Highlight

For peer review only - http://bmjopen.bmj.com/site/about/guidelines.xhtml

> *"The cost to the patient in time and effort to get to the surgery,…what time these appointments would be. How long they would take, how much effort it would take to get the patient… and also, if it's a research project, I always feel that patients shouldn't wait, …it's up to the nurse to make her appointments work so that she doesn't have anybody waiting there for any length of time,*

Formatted: Font: Italic

Research nurse interview no 1

Altruism

Many interviewees also perceived that where participants entered a trial for altruistic reasons they were more likely to remain in the trial.

> *"…They [participants] feel as though they want to do good because .. often a lot of them say "oh it's payback time", they think the NHS has been good to them so they want to give something back".*
>
> ***Trial manager interview 7***

Perceived benefit of trial participation

Most interviewees thought that if trial participants perceived an extra benefit from participating in a trial that this would contribute to retention particularly if they are being monitored for disease progression.

> *"In studies where (there) is … constant monitoring ....they don't mind giving..... samples again and again as part of the trial..... But I …think …. if they can see some benefit then they'll do it but if they can't or if they've already got that benefit from giving the sample at the first time point they might not want to do it again and again"*
>
> ***Trial manager interview 10***

Some of the benefits identified from participation were extra health monitoring for example: *"ECG" "BP"* and *"cholesterol"* as well as the extra attention from staff. However, these perceived benefits were thought to be trial dependent.

A~~Participant age~~

Several age groups were highlighted as challenging for trial retention, for example, teenagers wishing to opt out of a trial when they reach the age of consent; working mothers juggling school runs with work time, and elderly participants who have either lost their independence or are involved with extended family caring activities. Young men with other interests were also thought to be challenging to retain in trials

For peer review only - http://bmjopen.bmj.com/site/about/guidelines.xhtml

"A young girl whose parents had agreed to be in the trial and then, you know, she didn't want to and then decided herself she was going to withdraw. And, you know, I can see the potential for that sort of thing if it's a trial in the sort of pre-teen, teen group that,... they might well change their mind….more interesting things going on in their lives, they've got more after school activities or they've got exams or they've got whatever. And coupled with being more independent and wanting to assert their own ideas".

Principal investigator interview 28

"In trials with older people, people will die, people will get frailer and maybe be institutionalised and not be able to continue or not want to continue for those sort of reasons".

Principal investigator interview 28

"There are all the usual things, we assume that older people don't move, there's a astonishingly high level of mobility in the older population. So lost to follow-up can occur just because people say "right, I'm going to go and live in…………..." or "I'm going to go and live near my daughter" or "I've off to ……….for the winter". [They] do… a lot of child care, grandparental child care and sorting out their children's divorces and so on and so they get distracted and drop out completely through distraction".

Principal investigator interview 21

Other participant characteristics

Feelings of guilt and shame with not having achieved positive outcomes in a trial were thought to contribute to loss to follow-up in some disease areas / conditions e.g. in treatment for obesity trials, if participants were not losing weight they might not return to be weighed perhaps due to the shame and stigma associated with not achieving their expected aims. Retention in trials involving behaviour change was also thought to be problematic if change targets were not achieved by participants. For example, interviewees were generally in agreement that retention can be problematic in trials involving participants with a mental health issue.

"In nutrition studies we're very much bound by looking at healthy individuals to begin with. So from that perspective they may certainly be less motivated than an individual participating in a drug trial who has a severe or debilitating condition that, you know, they want to try and do something about".

Trial manager interview 15

For peer review only - http://bmjopen.bmj.com/site/about/guidelines.xhtml

BMJ Open qualitative paper submitted Version 3 VB 26.11.13

"For smoking cessation or weight management or any of these things where certain behaviour is expected or wanting to be demonstrated. If they're not achieving it they are more likely to just drop out"

Research nurse interview 9

"Bi-polar groups, a lot of them find it very, very difficult going to an NHS facility".

Trial manager interview 17

"it depends on what area of medicine it is, for instance, if you're dealing with people with psychiatric illness, there's always a problem with attendance, whether that's within the surgery or whether that's within research".

Research nurse interview 1

The interviewees felt that participants with chronic diseases may have more interest in the treatment of their condition and return for follow-up visits. Others thought healthy volunteers may drop out of trials because of lack of interest in the trial.

~~1.~~2. Engaging with participants

Interviewees thought that engaging with the participants during trial recruitment and maintaining a good rapport was key to retention~~The recruitment visit was seen as a very important time for engaging participants and giving them important information about trial follow up procedures~~.

Formatted: Not Highlight

"It [the recruitment visit] sets the stage for the participants relationship with the trial ………… it's important to get that right, right from the start…. that affects whether people think it's worthwhile coming back…"

Trial manager interview 26

~~Establishing a rapport with participants that continues throughout follow-up was also thought to contribute to retention.~~

"At the end of the day it's how the nurse and GP …….establish a rapport and a relationship with the participant,… that is the key issue in keeping participants in a study".

Research nurse interview 12

~~Good rapport was thought to be maintained through continuity of trial staff at the trial site for follow up visits. Some TMs thought that having "*personal*" or "*good*" communication with~~

For peer review only - http://bmjopen.bmj.com/site/about/guidelines.xhtml

participants and within the research team itself contributed to participants returning to sites for follow-up visits .

> *"I think it is communication at all levels; so the nurse who interfaces with the trial management team behind the coordinating centre, ... listening to the participant's views ...and ... feeding them back, ... a two-way process. Because we're this side doing the research, we have to listen ... so yes, ... communication and understanding [the] population that you're working with".*
>
> ***Trial manager interview 13***

TMs have strategies in place to increase response to postal questionnaires using different communication methods to engage with trial participants from the coordinating centre e.g. postal and telephone methods.

> *"We .....mak(e) sure that we have [communication] strategies in place to get as many .....responses to our postal questionnaires ...we send out the questionnaire..., if we don't hear back ....we will ring up to say ...."Could you possibly send it back to us?" If we still don't hear ...we'll send .. another copy ... if we don't have a response from that copy then we'll ring them again...if we still don't hear we've telephoned ...again to collect a set of core outcomes to get a response............"*
>
> ***Trial manager interview 14***

Some interviewees across all groups mentioned the impact of trial staffthe personalities of trial staff on trial retention. Interviewees thought trial RNs needed to have a "*flexible approach*" and to "*be sympathetic*" toward participants, while TMs needed to be "*competent*", "*personable*", "*persistent*", "*enthusiastic*", and "*good communicators*".

> "*If you're enthusiastic, can give the information to the participants and...[are] sympathetic to .... queries, always being accommodating,[and] getting back to them if they have any queries ...being absolutely spot on with, so that they don't feel they've been let down or you haven't been bothered to get back to them."*
>
> ***Research nurse interview 5***

> "*I think there's a paradox here because they [trial managers] need to be obsessional about detail but they also need to be relaxed and flexible in their response to different situations ....and problems that come up. That's asking quite a lot, I think. So you know it's great if you find somebody who's good at it.....Or be very kind hearted. And prepared to work out of hours!"*
>
> 2. ***Principal investigator interview 25***

Formatted: Indent: Left: 0.25", Line spacing: single, No bullets or numbering

For peer review only - http://bmjopen.bmj.com/site/about/guidelines.xhtml

BMJ Open qualitative paper submitted Version 3 VB 26.11.13

Perceived benefit of participation to the participant

Most interviewees thought that if trial participants perceived an extra benefit of participation this would contribute to retention in the trial particularly if they are being monitored for disease progression.

> *"In studies where (there) is ... constant monitoring ....they don't mind giving..... samples again and again as part of the trial..... But I ...think .... if they can see some benefit then they'll do it but if they can't or if they've already got that benefit from giving the sample at the first time point they might not want to do it again and again"*
>
> ***Trial manager interview 10***

Some of the benefits identified were extra health monitoring for example: *"ECG" "BP" "cholesterol"* and other *"health checks"* as well as having the extra attention from staff. However, these perceived benefits were thought to be trial dependent.

participant altruism, where people :

> *"...They [participants] feel as though they want to do good because they often, a lot of them say "oh it's payback time", they think the NHS has been good to them so they want to give something back".*
>
> ***Trial manager interview 7***

3. Tailoring retention strategies

Tailoring tailoring specific retention trial retention strategies to enable different trial population groups to either return questionnaires or return to sites for follow-up was thought to improve retention. For example inIn mental health trials for example,RCTs, some interviewees had found that some participants may wish to be followed-upseen at home rather than at than returning to a clinical sites for follow-up visitssite.

> *"Depending on the trial and the length of the trial and your client group ...you would have a completely different strategy for... the fifteen to twenty-four year olds, to something... where you're looking at the seventy-five plus..... you do sort of tailor it [the strategy] to your group."*
>
> ***Research nNurse interview 2***

Factors thought to contribute to loss to follow-up in primary care RCTs (Box 2)

Formatted: Font: Bold

Formatted: List Paragraph, Numbered + Level: 1 + Numbering Style: 1, 2, 3, … + Start at: 1 + Alignment: Left + Aligned at: 0" + Indent at: 0.25"

Formatted: Font: Bold

Formatted: List Paragraph, Numbered + Level: 1 + Numbering Style: 1, 2, 3, … + Start at: 1 + Alignment: Left + Aligned at: 0" + Indent at: 0.25"

Formatted: Not Highlight

Formatted: Not Highlight

Formatted: Not Highlight

For peer review only - http://bmjopen.bmj.com/site/about/guidelines.xhtml

BMJ Open qualitative paper submitted Version 3 VB 26.11.13

Participant age was thought to affect retention in primary care RCTs. Several age groups were highlighted as challenging, for example, teenagers opting out of a trial when they reach the age of consent; working mothers juggling school runs with work time and follow-up visits; elderly participants either losing their independence or being involved with extended family caring activities, and young men with other interests.

"A young girl whose parents had agreed to be in the trial and then, you know, she didn't want to and then decided herself she was going to withdraw. And, you know, I can see the potential for that sort of thing if it's a trial in the sort of pre-teen, teen group that,... they might well change their mind....more interesting things going on in their lives, they've got more after school activities or they've got exams or they've got whatever. And coupled with being more independent and wanting to assert their own ideas".

Principal investigator interview 28

"In trials with older people, people will die, people will get frailer and maybe be institutionalised and not be able to continue or not want to continue for those sort of reasons".

Principal investigator interview 28

"There are all the usual things, we assume that older people don't move, there's a astonishingly high level of mobility in the older population. So lost to follow-up can occur just because people say "right, I'm going to go and live in………..." or "I'm going to go and live near my daughter" or "I've off to ……....for the winter". [They] do… a lot of child care, grandparental child care and sorting out their children's divorces and so on and so they get distracted and drop out completely through distraction".

Principal investigator interview 21

Trial participants' feelings of guilt and shame with not having achieved positive outcomes in a trial were thought to contribute to loss to follow-up in some disease areas / conditions. Some interviewees thought that if participants did not perceive a benefit from participation they might not return for their follow-up visit. They thought for example that in treatment for obesity RCTs, if participants were not losing weight they might not return to be weighed perhaps due to the shame and stigma associated with not achieving their expected aims. Retention in RCTs involving behaviour change was thought to be problematic if change targets were not achieved by participants. For example, interviewees were generally in

For peer review only - http://bmjopen.bmj.com/site/about/guidelines.xhtml

~~agreement that retention can be problematic in RCTs involving participants with a mental health issue.~~

> ~~*"In nutrition studies we're very much bound by looking at healthy individuals to begin with. So from that perspective they may certainly be less motivated than an individual participating in a drug trial who has a severe or debilitating condition that, you know, they want to try and do something about".*~~
>
> ~~***Trial manager interview 15***~~

> ~~*"For smoking cessation or weight management or any of these things where certain behaviour is expected or wanting to be demonstrated. If they're not achieving it they are more likely to just drop out"*~~
>
> ~~***Research nurse interview 9***~~

> ~~*"Bi-polar groups, a lot of them find it very, very difficult going to an NHS facility".*~~
>
> ~~***Trial manager interview 17***~~

> ~~*"it depends on what area of medicine it is, for instance, if you're dealing with people with psychiatric illness, there's always a problem with attendance, whether that's within the surgery or whether that's within research".*~~
>
> ~~***Research nurse interview 1***~~

~~Interviewees also felt that participants with chronic diseases may have more interest in the treatment of their condition and return for follow-up visits which would contribute to retention. Others thought healthy volunteers may drop out of RCTs because of lack of interest.~~

4. Working environment

Some interviewees reported that the working environment at general practice trial~~RCT~~ sites might impact upon ~~-~~retention. ~~and that it~~ It was thought to be important to have a staff member at the site to "*champion*" the study~~ locally~~. Part-time RNs reported working in isolation and~~, and~~ sometimes struggled to find a consulting room for follow-up visits which~~, restricting~~ restricted the availability of flexible follow-up appointment times to offer to participants.

> *"The last study that I worked on, ..I struggled to find .. a room, the practice were incredibly accommodating, and really wanted me to have a room, but it just is very, very tight. ...it has been, a major issue for me...It was affecting follow up for me because I was not always able to have a room when I was available, that ....would*

For peer review only

have been potentially available for participants, so it just narrowed down the opportunities for me to offer to participants to come in"

Research nurse interview 5

"if somebody genuinely did want to make the trial work then it was possible to make it work. ………………..it needed somebody to actually manage the trial within that practice. The practices in which we had success I felt that we had a champion there who was fighting our corner for us"

Trial manager interview 27

"the other thing is practice awareness, you know, because you said it's a big practice and if somebody comes in and hasn't got the sort of clock-in bit straight and they go to the desk and say, I've come to see X about this trial and, you know, new receptionist knows nothing. That can be quite off-putting so as long as everybody's engaged …and everybody knows about it".

Research nurse interview 2

Discussion

Statement of principal findings

Based on in-depth interviews with primary care trialRCT personnel we identified that incentive, communication and questionnaire strategies are used to try to increase retention in primary care trialsRCTs. Participant characteristics e.g. interest in the trial, altruism and time, encourage retention. Making efficient use of participant time and appropriate engaging with them were also thought to promote increase retention as was the perception by participants that they would have some benefit from participation.. Interviewees also thought that participant's perceptions of benefits from participation; the age of participants —e.g. young men and teenagers; the disease area / condition e.g. mental illness and behavioural problems; and the research environment in which the trial was conducted e.g. availability of consulting rooms for follow-up appointments were all contributed contributing factors to loss to follow-up in trialsRCTs.

Formatted: Not Highlight

Strengths and weaknesses

There is limited work onResearchResearch on strategies to improve retention of participants retention in the retention of participating individuals in the area of primary care trialsRCTs.

For peer review only - http://bmjopen.bmj.com/site/about/guidelines.xhtml

BMJ Open qualitative paper submitted Version 3 VB 26.11.13

~~Work to date~~ ~~-~~is limited and commonly grouped with recruitment strategies~~ for participants and trial sites~~[14;15]. ~~[12;1312,13]~~. Trial retention is important because loss to follow-up can lead to incomplete data for the primary outcome, bias results and impact on the generalisability of trial findings. This study is the first to explore the use of retention strategies in a broad spectrum of randomised UK primary care trials~~RCTs~~ and to identify~~the~~ factors associated with retention~~ and loss to follow-up~~ separate from recruitment strategies. Interviews were conducted with researchers experienced in ~~the~~ design, leadership, management, and implementation ~~at site level~~ of randomised UK primary care trials, many of whom reported ~~that~~~~RCTs most of whom had used strategies to improve retention. Pprior~~that prior to this study ~~some of the interviewees reported~~ that they had not thought deeply about loss to follow-up or ~~the~~ strategies used to overcome this. With the focus by funders on recruitment rather than retention targets, this study specifically raises awareness of the complexity of retention in primary care ~~RCTs among primary care researchers~~trials and the many strategies and factors used to maximise retention. Although the thoughts and experiences of trial participants were not captured to further ~~A limitation of this study is that the thoughts and experiences of trial participants who have dropped out or of those who were retained in RCTs were not captured to further~~ explore the barriers and facilitators to retention~~ in primary care RCTs~~ the results show that trial personnel have an understanding of the challenges participants encounter to stay in trial follow-up. ~~Also it~~It is ~~not clear~~unclear how generalisable the results are to randomised trials~~RCTs in other areas~~ outside of ~~those conducted in the context of~~ primary care~~-~~.

Meaning and implications for the use of retention strategies

These results enhance the interpretation and provide insight around the implementation of the results of ~~the~~our Cochrane review on ways to improve retention in trials[2]~~RCTs~~[21] ~~ogether, thes, and used together, can inform the future use of effective strategies to improve retention in primary care RCTs.~~. Together, these two studies inform the future use of effective strategies to improve retention in primary care trials.

Field Code Changed

There was general agreement ~~across the groups~~ that small monetary incentives were likely to be viewed favourably by ethics committees ~~acceptable in the context of~~and led to ~~increaseding~~increased questionnaire response, in ~~agreememnt~~agreement with the findings of the Cochrane Review[2;22]. ~~, and were likely to be viewed favourably by ethics committees,~~ ~~a~~~~Although~~Although there was uncertainty among those interviewed about whether monetary

Field Code Changed

For peer review only - http://bmjopen.bmj.com/site/about/guidelines.xhtml

BMJ Open qualitative paper submitted Version 3 VB 26.11.13

incentives should be given up front or offered on receipt of data, ~~. T~~t~~he~~he Cochrane review ~~shows~~ showed that ~~both~~ either approach increased postal questionnaire response[22 ~~21~~] ~~.~~.

Field Code Changed

Formatted: Do not check spelling or grammar, Superscript

Scepticism among interviewees about ~~N~~~~non~~non-monetary incentives ~~are also used in primary care trialsRCTs, but with considerable scepticism as to~~and their value ~~to the extent that it was thought that these could have a negative impact on trial retention and questionnaire response.~~ w~~W~~as also supported by the Cochrane Review~~,~~ which did ~~As this type of incentive was~~ not ~~found~~ find them to be effective ~~in the Cochrane review.~~ ~~, primary care trialists or indeed any Ttrialists.~~ Trialists may therefore ~~wish~~ need to reconsider the use of this type of incentive ~~in trialsRCTs~~.

~~A range of different Ccommunication strategies were discussed by interviewees. Although trialists routinely appeapreferred red to be commonly used to increase return to sites for follow up and to improve return of questionnaires. 1st class post, and the use of enhanced letters, the Cochrane Review found no evidence to support either approact. is used routinely for outgoing post. Based on two studies, Edwards systematic review found that 1st class post was effective at increasing questionnaire response but was not specific to RCTs. However the Cochrane review of strategies in RCTs demonstrates no benefit of using 1st class post for outgoing mail[14]. Therefore, 2nd class post and standard letters can probably be used for postal correspondence with primary care trial participants, with little impact in loss to follow up. Also, some trial managers did post letters to trial participants so that they would arrive on a Saturday.~~ A range of different communication strategies were discussed by interviewees. Although trialists routinely preferred 1st class post, and the use of enhanced letters, the Cochrane Review found no evidence to support either approach. Therefore, 2nd class post and standard letters can probably be used for postal correspondence with primary care trial participants, with little impact on loss to follow-up.

Formatted: Superscript

~~Although~~ Trialists had mixed opinions on the use of recorded delivery, ~~was used for important study related materials and to resend questionnaires, this was thought to be potentially frustrating for participants if delivery was made while they were out.~~ ~~nNevertheless~~nevertheless recorded delivery of questionnaires compared with a telephone reminder was found to be effective for improving questionnaire response[2;5] ~~2;51,5~~. To avoid any inconvenience associated with the use of this strategy, preplanning the delivery time with the

For peer review only - http://bmjopen.bmj.com/site/about/guidelines.xhtml

participant with a s and reminding themreminder to expect a recorded delivery of trial materials is recommended if this strategy is to be used in future trialsRCTs. The trial managers describe enhancing the letters they send. However, the Cochrane review showed no effect for using such enhanced letters[2], although the TDM, a postal communication package that encompasses sending hand prepared letters with a series of reminders to improve questionnaire response, was found to be effective based on the results of one trial reported in 1996[15]. TDM may not be applicable to all participant groups (e.g. young people) or for trials[21]. Although the TDM, a communication package that encompasses sending hand prepared letters with a series of reminders, to improve questionnaire response, was found to be effective, based on the results of one trial reported in 1996[15]. This approach may not be applicable to all participant groups (e.g. young people), or for RCTs using electronic data collection methods.

Different types of reminders and alternative questionnaires are routinely used by primary care researchers to improve trial follow-up, hHowever, there is no but our Cochrane review found noclear evidence that reminders and prompts in addition to the standard trial follow-up procedures are beneficial to retention. Also, Edwards found that telephone reminders did notor to improveing questionnaire response[2]. Similarly, there is no evidence tto suggest better response rates to shorter questionnaires[2]. Different types of reminders are routinely used by primary care researchers to improve trial follow-up, but the Cochrane review found no clear evidence that reminders and prompts in addition to the standard trial follow-up procedures are beneficial to retention. Also, Edwards found that telephone reminders did not improve questionnaire response[14]. Reminders are part of the standard trial follow-up procedures in RCTs for collection of data from non-responders and. They can play an important part in participant engagement especially if there is little face to face contact with trial participants. However, the interviewees in this study thought too many additional reminders could be perceived as harassment by trial participants and lead to loss to follow-up.

Several alternative questionnaire formats were thought to be potentially useful for increasing response, but not are used as first-line strategies. Shorter questionnaires were used by some interviewees to increase questionnaire response. Although Edwards found that shorter questionnaires increased questionnaire response in research generally[14], this was not borne out in trials by our Cochrane review[2]. A strategy also mentioned but not yet evaluated is to divide

Formatted: Do not check spelling or grammar, Superscript

Formatted: Do not check spelling or grammar, Superscript

Comment [FS1]: Do you need retention strategies for this type of research?

To improve response to electronic questionnaires?

Field Code Changed

Formatted: Do not check spelling or grammar, Superscript

Formatted: Do not check spelling or grammar, Superscript

For peer review only - http://bmjopen.bmj.com/site/about/guidelines.xhtml

~~long questionnaires into several short questionnaires to reduce the burden for participants of data collection associated with longer questionnaires. This may not be practical in trials[14], this was not borne out by the Cochrane review in RCTs [21]. A strategy also mentioned but not yet evaluated is to divide long questionnaires into several short questionnaires to reduce the burden for participants of data collection associated with longer questionnaires. This may not be practical in RCTs where several there are a number of follow-up questionnaires are to be completed.~~

~~Behavioural and methodological strategies were not used in the context of these UK primary care RCTs.~~ Furthermore, ~~The~~ interviewees were particularly negative toward the use of behavioural strategies and there was no effect found for these in our~~the~~ Cochrane review[2]. ~~[21].~~ ~~Case management, although seldom used, was thought to be potentially useful by interviewees, however, there is no evidence that this is effective[2]. The interviewees also thought that blinding participants to their allocated intervention would improve retention. This is in contrast to findings from the. However, our Cochrane review which found that an open trial design was more effective than a blind design in the context of secondary osteopathic fracture one trialprevention[16]. Avenell argues that double blind RCTs do not reflect usual health care procedures, and that open RCTs, where the intervention is compared to no treatment or usual treatment, could give a better measure of the differential effects in normal care settings[16]. The open trial design, if used as a stand alone strategy to improve retention, would need to be considered in the context of the overall aims and objectives of the trial. Further evaluation of this strategy is needed in different trial contexts and settings if it is to be adopted as a strategy to improve trial retention. Although case management was seldom used, elements of this strategy were thought to be useful but there is no evidence that it is effective[21].~~ Case management, although seldom used, was thought to be potentially useful by interviewees, however, there is no evidence that this is effective[2]. The interviewees also thought that blinding participants to their allocated intervention would improve retention. This is in contrast to findings from the Cochrane review which found that an open trial design was more effective than a blind design in one trial[2;4]. Avenell argues that double blind trials do not reflect usual health care procedures, and that open trials, where the intervention is compared to no treatment or usual treatment, could give a better measure of the differential effects in normal care settings[4]. The open trial design, if used as a stand-alone strategy to improve retention, would need to be considered in the context of the overall aims and

Field Code Changed

For peer review only - http://bmjopen.bmj.com/site/about/guidelines.xhtml

BMJ Open qualitative paper submitted Version 3 VB 26.11.13

objectives of the trial. Further evaluation is needed in different trial contexts and settings if this retention strategy is to be adopted.

Several other factors and ways to ~~aimed at improving~~improve retention were identified that were not covered in our Cochrane review[2]. Some of these ~~strategies~~ such as ~~the~~ relationships and communication between trial staff and trial participants are clearly more difficult to design interventions for and to evaluate. The factors identified indicate a need to train and support trial staff in ways to improve trial retention. It seems clear from this qualitative study that trial staff were oriented to participant~~tient~~ concerns and needs (as they perceived them) and had a high level of empathy with participants.

Integrating qualitative research with Cochrane reviews

Integrating the results of the qualitative study and the Cochrane review helped us understand the different experiences ~~of~~ primary care researchers have with the ~~in the use of the retention~~ strategies identified by our Cochrane review[2]~~–~~. The qualitative results support some of the results of our~~the~~ Cochrane review and demonstrate the potential transferability of the different effective retention strategies to trials~~RCTs~~ conducted in primary care. For example incentives were employed in different~~in different~~ ways to try to improve responses to postal questionnaires ~~used in primary care RCTs.~~. The results have also helped identify other factors that might promote retention such as building a rapport with trial participants, and potential barriers like finding a consulting room for follow-up appointments. This mixed methods approach could be replicated in ~~other~~ research contexts apart from primary care to determine how appropriate and useful the strategies identified by the Cochrane review[2] are in practice.

While the absolute effects of effective strategies identified by the Cochrane review are modest[2]~~.~~, this qualitative study highlights that multiple strategies are often used to try to achieve maximum retention in primary care ~~RCTs. Also~~trials. Furthermore it suggests that clinical trialists~~primary care researchers~~ should consider other factors that may affect retention, such as building a rapport with participants and minimising the impact of trial follow-up on their daily lives. Consideration should also be given to the characteristics of the intervention, participants and trial personnel when choosing effective strategies identified by our ~~the~~ Cochrane review ~~are being considered~~ to improve trial retention[2].

Future research

Field Code Changed

Formatted: Do not check spelling or grammar, Superscript

Field Code Changed

For peer review only

For peer review only - http://bmjopen.bmj.com/site/about/guidelines.xhtml

It was clear from the interviews that there is a move toward contacting participants by email and a desire to use text reminders when automated facilities for these become available for use in randomised trials~~RCTs~~. These new strategies would need evaluation in future trials~~RCTs~~.

Further research is also needed to identify potential barriers and facilitators to follow-up in trials~~RCTs~~ e.g. with trial participants from different social, economic, age and disease groups. The results would be useful for primary care researchers and would help target and tailor strategies to meet the needs of different population groups to keep them specifically engaged in trial follow-up[16] . ~~[17]. Incorporating the views of trial participants, including those who have withdrawn, in future research is challenging but would enhance understanding what works best and why.~~

Field Code Changed

Conclusion

Qualitative research can help to interpret the results of systematic reviews by telling us what researchers do or think happens in research practice. These findings provide a deeper understanding of -the factors that may facilitate retention such as rapport with participants and respect for their time, and highlights the participant and environmental characteristics that can limit the implementation of effective strategies. The use of small monetary incentives to improve questionnaire response is ~~effective and~~ acceptable however a~~. A~~ reconsideration of the use of non-monetary incentives, and certain communication strategies including 1st class post is needed. A similar exploration of barriers and facilitators to retention in other research contexts would help identify ways to improve retention in trials~~RCTs~~ conducted in other research contexts.

For peer review only - http://bmjopen.bmj.com/site/about/guidelines.xhtml

BMJ Open qualitative paper submitted Version 3 VB 26.11.13

Article Summary

Article focus

High levels of retention in trialsRCTs are important in order to maintain power and minimise bias. Strategies to improve retention are widely used in trialsRCTs but without evidence of effect.

Strategies thought to improve retention in UK primary care randomised controlled trials (RCTs) have not been well documented or evaluated. This study explores the use, in UK primary care trialsRCTs, of strategies to improve retention. identified by a Cochrane review. It also explored factors associated with retention and attrition.

Key messages

Small monetary incentives, non-monetary incentives and communication strategies are used in primary care trialsRCTs to improve retention. Behavioural, methodology and case management strategies were not used. Small monetary incentives and communication strategies were thought to be useful for retention and case management thought to be potentially useful. Interviewees were negative about the use of behavioural, methodology and non-monetary incentive strategies.

Factors thought to contribute to retention are: effective communication, participant altruism and perceived benefits of participation for participants, burden reduction, personality and flexibility of trial staff.

Factors thought to contribute to loss to follow-up are: lack of time and interest in the trial, physical constraints, and limitations of research facilitiesparticipant age, disease/condition and research environment.

Formatted: Not Highlight

Formatted: Not Highlight

Strengths and limitations

This study is the first to explore the use of retention strategies in UK primary care trials andRCTsand the factors associated with retention and loss to follow-up. Interviews were conducted with researchers experienced in UK primary care trialsRCTs, many of whom had used strategies to improve retention.

For peer review only - http://bmjopen.bmj.com/site/about/guidelines.xhtml

A limitation is that the thoughts and experiences of trial participants have not been gathered to further explore the barriers and facilitators to retention in primary care ~~RCTs~~trials.

Acknowledgements

We would like to thank all twenty nine interviewees who gave of their time and thoughts on the complexities of trial retention in primary care.

Statement of funding and competing interests

All authors have completed the ICMJE uniform disclosure form and declare no competing interests. This project was funded by the Medical Research Council Population Health Sciences Research Network grant number PHSRN 30

For peer review only

For peer review only - http://bmjopen.bmj.com/site/about/guidelines.xhtml

Reference List

(1) Janson SL, Alioto ME, Boushey HA. Attrition and Retention of Ethnically Diverse Subjects in a Multicenter Randomized Controlled Research Trial. *Control Clin Trials* 2001; 22(6, Supplement 1):S236-S243.

(2) Brueton VC, Tierney J, Stenning S, Nazareth I, Meredith S, Harding S et al. Strategies to improve retention in randomised trials . *Cochrane Methodology Group* 2013; in press.

(3) Sutherland HJ, Beaton M, Mazer R, Kriukov V, Boyd NF. A randomized trial of the total design method for the postal follow-up of women in a cancer prevention trial. *Eur J Cancer Prev* 1996; 5(3):165-168.

(4) Avenell A, Grant AM, McGee M, McPherson G, Campbell MK, McGee MA et al. The effects of an open design on trial participant recruitment, compliance and retention - a randomized controlled trial comparison with a blinded, placebo-controlled design. *Clinical Trials* 2004; 1(6):490-498.

(5) Tai SS, Nazareth I, Haines A, Jowett C. A randomized trial of the impact of telephone and recorded delivery reminders on the response rate to research questionnaires. *J Public Health* 1997; 19(2):219-221.

(6) McColl EM, Eccles MPM, Rousseau NSB, Steen INP, Parkin DWD, Grimshaw JMP. From the Generic to the Condition-specific?: Instrument Order Effects in Quality of Life Assessment. [Article]. *Med Care* 2003; 41(7):777-790.

(7) Cockayne S, Torgerson D. A randomised controlled trial to assess the effectiveness of offering study results as an incentive to increase response rates to postal questionnaires [ISRCTN26118436]. *BMC Medical Research Methodology* 2005; 5(1):34.

(8) Man MS, Tilbrook HE, Jayakody S, Hewitt CE, Cox H, Cross B et al. Electronic reminders did not improve postal questionnaire response rates or response times: a randomized controlled trial. *J Clin Epidemiol* 2011; 64(9):1001-1004.

(9) Dangour AD, Allen E, Elbourne D, Fasey N, Fletcher AE, Hardy P et al. Effect of 2-y n-3 long-chain polyunsaturated fatty acid supplementation on cognitive function in older people: a randomized, double-blind, controlled trial. *Am J Clin Nutr* 2010; 91(6):1725-1732.

(10) Williamson I, Benge S, Barton S, Petrou S, Letley L, Fasey N et al. Topical intranasal corticosteroids in 4-11 year old children with persistent bilateral otitis media with effusion in primary care: double blind randomised placebo controlled trial. *BMJ* 2009; 339(dec16_1):b4984.

(11) Trial Managers Network. 2013.
Ref Type: Online Source

Formatted: Space After: 12 pt

Formatted: Font: Italic, Do not check spelling or grammar

Formatted: Font: Italic, Do not check spelling or grammar

Formatted: Font: Italic, Do not check spelling or grammar

Formatted: Font: Italic, Do not check spelling or grammar

Formatted: Font: Italic, Do not check spelling or grammar

Formatted: Font: Italic, Do not check spelling or grammar

Formatted: Font: Italic, Do not check spelling or grammar

Formatted: Font: Italic, Do not check spelling or grammar

Formatted: Font: Italic, Do not check spelling or grammar

Formatted: Font: Italic, Do not check spelling or grammar

Formatted: Space After: 12 pt

For peer review only

Qualitative retention paper BMJ submission 18.06.13

(12) Pope C, Ziebland S, May N. Analysing qualitative data. In: Catherine Pope, Nicolas May, editors. Qualitative Research in Health Care. second ed. London: BMJ Publishing Group; 2000. 75-88.

(13) Farmer T, Robinson K, Elliott SJ, Eyles J. Developing and Implementing a Triangulation Protocol for Qualitative Health Research. *Qual Health Res* 2006; 16(3):377-394.

(14) Leathem C, Cupples M, Byrne M, O'Malley M, Houlihan A, Murphy A et al. Identifying strategies to maximise recruitment and retention of practices and patients in a multicentre randomised controlled trial of an intervention to optimise secondary prevention for coronary heart disease in primary care. *BMC Medical Research Methodology* 2009; 9(1):40.

(15) Graffy J, Grant J, Boase S, Ward E, Wallace P, Miller J et al. UK research staff perspectives on improving recruitment and retention to primary care research; nominal group exercise. *Fam Pract* 2009; 26(1):48-55.

(16) Kimmel SE, Troxel AB. Novel incentive-based approaches to adherence. *Clin Trials* 2012; 9(6):689-695.

Formatted: Font: Italic, Do not check spelling or grammar

Formatted: Font: Italic, Do not check spelling or grammar

Formatted: Font: Italic, Do not check spelling or grammar

Formatted: Font: Italic, Do not check spelling or grammar

Formatted: Indent: Left: 0", Hanging: 0.5", Tab stops: 0.38", Right + 0.5", Left

For peer review only

For peer review only - http://bmjopen.bmj.com/site/about/guidelines.xhtml

Table 1 Characteristics of interviewees

Characteristics	Number of interviewees
Male	10
Female	19
**Role**	
Research nurse	9
Principal investigator	10
Trial manager	10
**Location**	
London	8
Midlands	8
Northeast England	3
Southwest England	3
East of England	5
Scotland	2
**Unit**	
University/Research organisation based	23
GP practice site based	6

Table 2 Other sample characteristics

Other characteristics	Number of interviewees
No of interviewees from trials published between 2000-2004	6
No of interviewees from trials published between 2005-2010	23
No of interviewees from trials conducted through the MRC GPRF	19
No of interviewees from trials conducted through other research units	10
No of interviewees recruited from trials with loss-to–follow-up levels*:	
>20%	10
<20%	17

* Two nurses not linked to a sample frame trial

For peer review only - http://bmjopen.bmj.com/site/about/guidelines.xhtml

Figure 1 Questionnaire design features reported by interviewees to improve questionnaire return

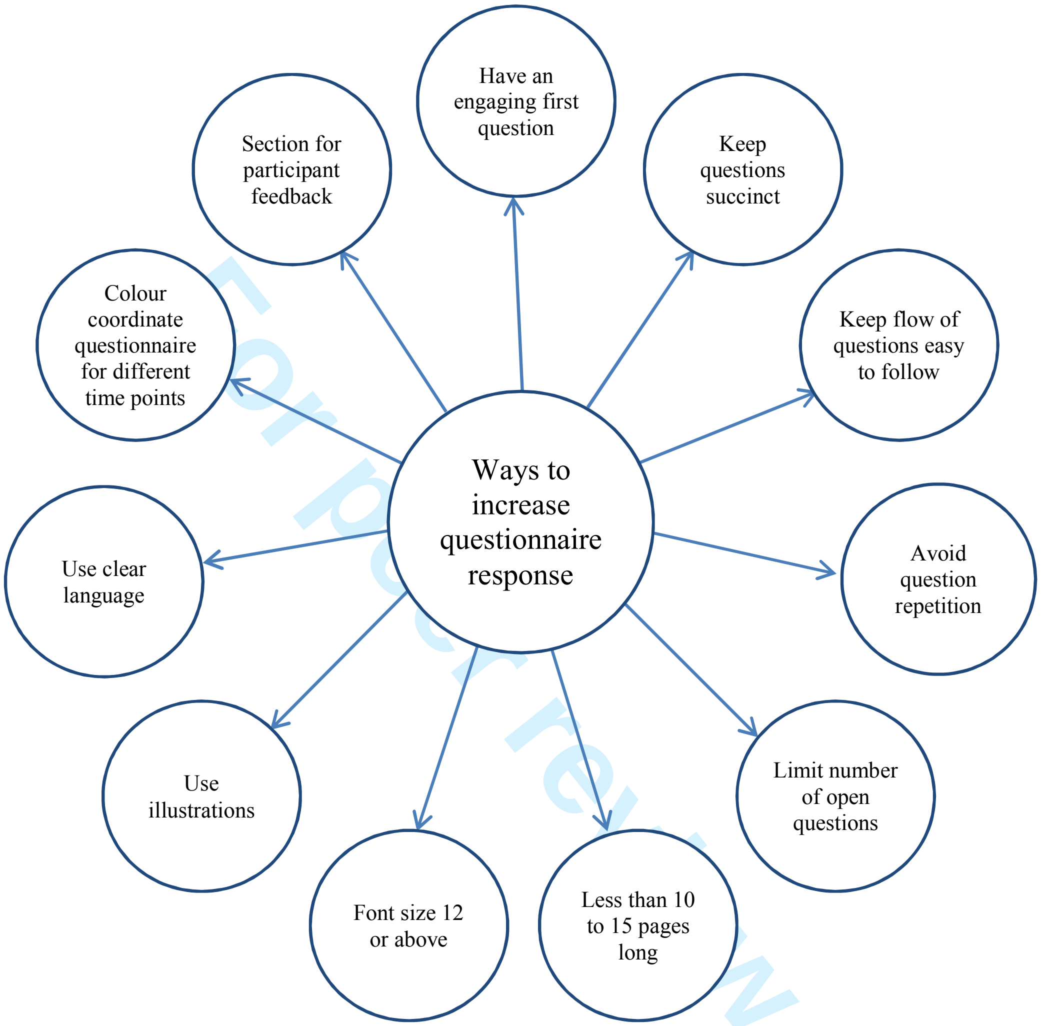

For peer review only

Web Appendix 1: Interview schedule

Background / introduction to the interview

I am interested in attrition from randomised trials. We define attrition as incomplete ascertainment of the primary outcome, but will subsequently use the term "loss to follow-up". I want to explore trialist's opinions on if and when loss to follow-up has been a problem in their experience and the strategies they may have used to deal with or to prevent loss to follow-up. I also want to find out more about trialists preferred strategies for preventing loss to follow-up.

Topic guide questions

1. **Can you tell me about recent trials you have worked on?**
   Were there any with high follow-up?
   Were there any with low follow-up?
2. **What do you think are the factors that lead to loss to follow-up in trials?**
   Why do you think it can be difficult to keep participants in randomised trials?
3. **What are the factors that lead to retention in randomised trials?**
4. **What strategies to increase follow-up have been successful for you in trials you have worked on?**
   Why do you think these have worked?
   What strategies have been unsuccessful?
   Why have these not worked?

Decision making around strategies used to reduce loss to follow-up

1. When do you perceive loss to follow-up to be a problem?
2. How do you decide which strategies to improve follow-up work best?
3. How did/ do you implement the strategies to improve follow-up that worked best?
4. How is loss to follow-up monitored in trials you have worked on?
5. Who deals with loss to follow-up when it presents?

Impact of research governance

1. What do you feel about using incentives to keep participants in trials?
2. Have you had any experience with ethics committees?
3. What do you feel about ethics committees asking about payments or giving other incentives to participants?
4. Has ethics committees approach to payments affected any trials you have worked on in anyway?

Ask this next question at the end of the interview

These are the strategies to reduce attrition/ improve retention identified by the Cochrane review.

For peer review only

Show participant each card separately (See below). Then ask the following questions for each strategy discussed:

1. Have you used these?
2. What do you think about using this strategy?
3. Might you have considered using this strategy in your trial?
4. What could be the advantages of using the strategy?
5. What could be the disadvantages of using this strategy?

Each card shown individually

**Card no 1**
Communication strategies. e.g. email, telephone, text messages, letters signed by different study personnel, type of delivery- e.g. post 1st 2nd class, or recorded delivery, type of envelope used for response.

**Card no 2**
Incentives to either participants or trialists e.g. gifts pens, pins, monetary incentives, offers of incentives, vouchers.

**Card no 3**
Methodological strategies blind versus un blind trials.

**Card no 4**
Different length of questionnaire: Short versus long.

**Card no 5**
Using case management. Having trial assistants manage participant follow-up, for example arranging transport and services to enable participants to keep trial follow-up appointments.

**Card no 6**
Motivational/educational strategies. Such as arranging workshops to give participants information about goal setting and time management.

For peer review only

Table 1 Characteristics of interviewees

**Characteristics**	**Number of interviewees**
Male	10
Female	19
**Role**	
Research nurse	9
Principal investigator	10
Trial manager	10
**Location**	
London	8
Midlands	8
Northeast England	3
Southwest England	3
East of England	5
Scotland	2
**Unit**	
University/Research organisation based	23
GP practice site based	6

Table 2 Other sample characteristics

**Other characteristics**	**Number of interviewees**
No of interviewees from trials published between 2000-2004	6
No of interviewees from trials published between 2005-2010	23
No of interviewees from trials conducted through the MRC GPRF	19
No of interviewees from trials conducted through other research units	10
No of interviewees recruited from trials with loss-to–follow-up levels*:	
>20%	10
<20%	17

* Two nurses not linked to a sample frame trial

For peer review only

Table 3 Use of retention strategies identified by the Cochrane review by primary care research team members

Strategy	Principal investigators	Trial managers	Research nurses
**Incentives**			
Monetary incentives	One PI had never used incentives	Used to increase response to postal questionnaires. Some split the incentive and gave participants a small incentive at different times e.g. £5 at randomisation and £5 at completion. Some send the incentive with the questionnaire; others on receipt of the questionnaire.	Mostly in pharmaceutical trials. Sometimes in academic trials.
Vouchers	Used in trials they lead	For those that had used vouchers, types used included: high street stores (M&S, ASDA, Tesco) online gift tokens (Amazon, Waterstones and WHS), or fresh fruit vouchers. Some had tailored the type of voucher to participant requests e.g. one interviewee used B&Q home improvement vouchers. These were sent by TMs with questionnaires or with a promissory element that on completion of questionnaires the participant would receive a voucher. Mobile phone top up vouchers used in trial to retain young people.	Administered at site level by research nurse. Used as a thank you from the trial team.
Gifts	One PI used gifts. Given for practical reasons i.e. carrying study materials	Used gifts. Seldom mentioned spontaneously. Used as reminders to keep trial at the forefront of participants and clinicians' minds or as a thank you for participants. Uncertainty about the effectiveness of gifts given on behalf of the team to either participants or clinicians.	RNs used gifts to retain people in randomised trials if prescribed. Seldom mentioned spontaneously. Used to increase recruitment rather than retention.
**Communication**			
Letter	Used in trials they lead	Uncertain of effectiveness. Trial managers signed letters sent from coordinating centre. Electronic and hand signatures used. Name of CI mentioned. Sometimes used letters as a stand-alone communication method.	Only used if specified in trial protocol/ study procedure manual.
Post	Used in trials they lead	2nd class used for pre-paid reply envelopes to reduce cost. 1st class used to send letters. Recorded delivery for second copy of questionnaire. Would confirm patient had moved. Hand delivered letters if participant difficult to contact.	Only used if specified in trial protocol/ study procedure manual. Sometimes hand delivered.
Telephone contact	Used in trials they lead	Used sometimes	Preferred method. Used for follow-up contact.
SMS texting	Used in trials they lead	Used when all other methods fail or if prescribed for follow-up	Used if prescribed. Seldom used.

For peer review only

Strategy	Principal investigators	Trial managers	Research nurses
Emails	Used in trials they lead	Used when email address given	Used when email address given.
New questionnaire formats	Some used shorter follow-up questionnaire with the second reminder.	Used a briefer follow-up questionnaire with a second reminder.	Short ones used for telephone interviews for non responders. Different lengths used for face to face interviews at site.
Behavioural	Not used	Not familiar with this	Not used
Case management	Not used	Used by one	Not used but aware of this
Methodology	Not used	Not used	Not used

For peer review only

Table 4 Speculation about the use of retention strategies identified by the Cochrane review in primary care randomised trials

Strategy	Principal investigators	Trial managers	Research nurses
**Monetary incentives**	Participation in pharmaceutical company trials paid. Payment could be perceived as bribery used to coerce participants, if the amount given is excessive e.g. >£50 acceptable to increase questionnaire response. Young people motivated by money. Older people participate for altruistic reasons. Some felt "cash in the hand" was a useful motivator. One suggested increasing the value of incentives for each questionnaire returned. Incentive values used £5 - £20 cash or vouchers. Considered reasonable for adults and children.	Could be perceived as bribery used to coerce participants, if the amount excessive >£50. Incentives are acceptable to increase questionnaire response. Uncertainty about when to administer incentives during follow-up. £5 - £20 considered reasonable for adults and children.	Could be perceived as bribery used to coerce participants, if amount is excessive i.e. >£50. Small incentives acceptable to increase questionnaire response. Participants should not be out of pocket. £5 - £20 considered reasonable for adults and children.
Vouchers	Some never use vouchers as incentives. Unclear if these were successful. Some thought vouchers might not be appropriate for some social groups. Generic vouchers most appropriate.	One TM never used vouchers. Unclear if these were successful. Vouchers thought to be less open to corruption, but a burden. Some TMs thought some vouchers might not be appropriate for some social groups. Generic vouchers are more appropriate.	Vouchers good for retention. Generic vouchers more have more universal application. Do not have to deal with cash at sites.
Gifts	Gifts not given on behalf of the study team by PIs. Interested to know what participants thought about gifts. Some thought these were patronising, a waste of money and associated with charity fundraising and participants might question the use of public money for this purpose. Some thought participants would prefer monetary alternatives. Participants would not participate in a trial for the gifts alone	For altruistic participants gifts could be perceived as "insulting" or "uninteresting". Some thought participants might be suspicious about the use of public money on gifts. This might be interpreted as wasteful or extravagant use of public money especially if motivated by altruism to participate. Could be associated with Charities and fund raising.	Gifts, offered or given, should be useful, appropriate, not patronising and tailored toward the disease/condition and participant group. One thought pens were "incidentals" not gifts. Felt gifts given would not retain participants in trials.
**Communication**			

For peer review only

Strategy	Principal investigators	Trial managers	Research nurses
Letter	Few comments on usefulness. Important factors tone and personalisation. Participant input important. Don't want to be seen as interfering in trial management. Mail outs boring and burdensome for trial managers.	Consistency of signatory more important than who signs. Name of CI mentioned. Some participants respond to authority. Franking with institutional logo used. Effect unknown. "Hate" brown envelopes, perceived as "bills" "junk mail" "letter from tax man". One thought no evidence that envelope colour mattered. Uncertain about hand written envelopes over preaddressed.	May not be opened by participants. Could get lost/ opened by someone else. Makes it look different with Logo. Might work.
Post	Not raised spontaneously by PIs	1st class used to send letters. More likely to be opened.	1st class post waste of money
Telephone	Involved in decisions about use of telephone contact. Calls should be conversational to build rapport with participant. Would be "daunting" if they heard from a PI. Young people change telephone numbers. Difficult to get hold of. Be careful not to harass participants.	Nurses use this, should use it more to keep in touch with participants, might increase retention. Reminder call before due to attend would increase retention. Calls may not be answered fear of telemarketing.	Prefer telephone to letters. Engage in dialogue. One thought telephone contact more intrusive letters. Be careful not to harass participants.
SMS texting	Good for young people. A system based on the automated NHS clinic appointments system would be useful. Need evidence it works.	Used for young people. Young people change numbers.	Good for young people. Utilising similar system to automated NHS clinic appointments system might be useful. Limited to people that can text
Email	Every mode should be considered	Used for people with busy lives	Good for people with busy lives
**New questionnaire formats**	One thought getting the balance right between length, readability, content and the acceptability of the topic were important. If this could be done without compromising what the questionnaire intends to measure. If questionnaire administered at clinic by nurse rather than post response could increase.	Long questionnaires considered off putting, no matter how engaged they were in a trial. Adding extra questions was another barrier to completion and non return. Having several short questionnaires could encourage return. Not so short that participants would not see the point of completion. Questionnaires administered at the clinic by a nurse rather than by post could increase response. The time at which questionnaires are posted was felt to be a factor associated with improved response. Questionnaires sent before a clinic visit enables the participant to complete a	Shorter questionnaires of 30-50 pages were thought to be long and off putting for participants to complete, no matter how engaged they were in a trial. The addition of extra questions that were not often analysed was another barrier to completion and non return. Having several short questionnaires was thought to encourage return, these should not be so short that trial participants would not see the point of completing them. People get used to questionnaires.

For peer review only

Strategy	Principal investigators	Trial managers	Research nurses
		questionnaire at home before their clinic visit.	
**Behavioural**	Don't understand it. May contaminate intervention. Generalisability of results questionable. "*Preachy*". Need evidence. Sounds like giving them a bit of the intervention. Could lessen effects of the treatment intervention.	Work shops about motivation condescending to participants. Don't know if it would work. Time consuming and expensive. May work with some groups. Don't like it and don't know much about it. Could not imagine using it. May be a burden for coordinators and participants.	Not heard of this. Workshop potential problems: non attendance, burden for participants and organisers, time commitment.
**Case management**	Where the intervention has to be reproduced could be problematic. Useful if there is not much benefit for participants. Takes time to arrange. Expensive. Annoying if participant does not turn up. Would not have thought of doing it. We do some of this.	Important for elderly. Costly. Should be done, rarely have luxury to do it. Should not be a substitute for good communication. Degrees of case management used. Logistics need working out.	Could be useful. Some heard about this. "*Cannot fail*", expensive, logistics need to be worked out. Useful for elderly. Some do elements of this. Personality of case manager important.
**Methodology**	Could not blind some participants to e.g. therapist lead interventions. Depends on study. Could bias results, blinding should be done where possible. Not suitable as a strategy to reduce attrition.	Do not know enough and never thought about it. Risks: would drop out if open and did not get allocation to preferred treatment.	Don't know enough about this. Participants could drop out if not in preferred allocation group.

For peer review only

Box 1 Factors thought to contribute to retention in primary care trials

Reducing the burden of participation for participants

"The burden of activity on the patients, how easy it is for them, how long it's going to take, how intrusive in terms of blood samples or answering huge long questionnaires, it's partly the complexity as well, if it's very simple for them to do, often you just want very simple data back from people, alive or dead, or specific complications, if it can be done either with a, increasingly with a click of a button by sending a text or an email to people or just a very you know, reply paid cards so you're just getting the data back from them, I suppose the other thing is….trying to do electronic follow-up as much as possible so you don't actually have to bother the patient, you have their consent to access their electronic records and either their Primary care data can be interrogated …a way of linking all of those together, like with Cancer Registry, Registrar General data, so I suppose the more you can automate it the easier it becomes".

Principal investigator interview 4

Establishing a rapport with participants

"I think with a lot of studies the rapport with whoever, the sort of teams that they're engaging with, you know, be that the GP and the practice nurse or be it a consultant and a research nurse, if they show interest and passion and enthusiasm for the study that's really, really helpful and then if they're sort of treated well when they come for the follow-up visits……"

Principal investigator interview 28

Effective communication with participants

"I think it is communication, communication at all levels; so the nurse who interfaces with the trial management team behind the coordinating centre, …but her or him listening to the participant's views as well and … feeding them back, so ……. a two-way process. Because we're this side doing the research, we don't know best, we have to listen to… which is, I guess, why we involve consumers in our work as well, so yes, just communication and understanding your population that you're working with".

Trial manager interview 13

"*We …..mak(e) sure that we have [communication] strategies in place to get as many …..responses to our postal questionnaires …we send out the questionnaire…, if we don't hear back ….we will ring up to say …."Could you possibly send it back to us?" If we still don't hear …we'll send .. another copy … if we don't have a response from that copy then we'll ring them again…if we still don't hear we've telephoned …again to collect a set of core outcomes to get a response………..*"

Trial manager interview 14

Personality of trial research staff

"[Being] friendly is very important. And I just think having the right approach to things in general. So

For peer review only

you have a 'can do' attitude rather than a inflexible attitude. You have to have somebody who... Or people who... people who are prepared to go out of their way to do things. And have a very person focused way of doing things."

Trial manager interview 26

"If you're enthusiastic, and can give the information to the participants with,…interest and,… also sympathetic to their, …. queries, always being accommodating, always getting back to them if they have any queries if, there are messages left, being absolutely spot on with, so that they don't feel they've been let down or you haven't been bothered to get back to them."

Research nurse interview 5

"I think there's a paradox here because they [trial managers] need to be obsessional about detail but they also need to be relaxed and flexible in their response to different situations ….that come up, different problems that come up. That's asking quite a lot, I think. So you know it's great if you find somebody who's good at it…..Or be very kind hearted. And prepared to work out of hours!"

Principal investigator interview 25

Flexibility of trial research staff

"They're [participants] do[you] a favour if they are taking part in a clinical trial so we need to try to be flexible, come very early morning …or stay later or they can come after work. Lunch time if it is, you know near to their work but of course like sometimes people work in the city and is away from where they live or it's a long journey. ….If a patient sees that they're going sit there for an hour and a half he says "I can't, I can't afford the time", ….. you get that, you know, quite often…"

Research nurse interview 12

Altruism of participants

"I've always been of the opinion that (…..)healthy volunteers, … they have what can be considered a less of a kind of vested interest in the study (…) they're doing it all out of the goodness of their hearts, there's not a lot in it for them"

Trial manager interview 26

Tailoring strategies to different follow-up groups

"Depending on the trial and the length of the trial and your client group and all those sort of things…you would have a completely different strategy for, you know, …..thinking of Chlamydia, the fifteen to twenty-four year olds, [compared] to something like [a trial involving the elderly] where you're looking at the seventy-five plus….. you do sort of tailor it [the strategy] to your group."

Research Nurse interview 2

\

For peer review only

Box 2 Factors thought to contribute to loss to follow-up in primary care trials

Participant age

Younger participants

"A young girl whose parents had agreed to be in the trial and then, you know, she didn't want to and then decided herself she was going to withdraw. And, you know, I can see the potential for that sort of thing if it's a trial in the sort of pre-teen, teen group that,... they might well change their mind....more interesting things going on in their lives, they've got more after school activities or they've got exams or they've got whatever. And coupled with being more independent and wanting to assert their own ideas".

Principal investigator interview 28

Older participants

"In trials with older people, people will die, people will get frailer and maybe be institutionalised and not be able to continue or not want to continue for those sort of reasons".

Principal investigator interview 28

"There are all the usual things, we assume that older people don't move, there's a astonishingly high level of mobility in the older population. So lost to follow-up can occur just because people say "right, I'm going to go and live in............." or "I'm going to go and live near my daughter" or "I've off to ....for the winter". [They] do… a lot of child care, grandparental child care and sorting out their children's divorces and so on and so they get distracted and drop out completely through distraction".

Principal investigator interview 21

Disease/condition health status

"Bi-polar groups, a lot of them find it very, very difficult going to an NHS facility".

Trial manager interview 17

"it depends on what area of medicine it is, for instance, if you're dealing with people with psychiatric illness, there's always a problem with attendance, whether that's within the surgery or whether that's within research".

Research nurse interview 1

"In nutrition studies we're very much bound by looking at healthy individuals to begin with. So from that perspective they may certainly be less motivated than an individual participating in a drug trial who has a severe or debilitating condition that, you know, they want to try and do something about".

Trial manager interview 15

Research environment at the general practice

"if somebody genuinely did want to make the trial work then it was possible to make it work. ……………….it needed somebody to actually manage the trial within that practice. The practices in which we had success I felt that we had a champion there who was fighting our corner for us"

Trial manager interview 27

For peer review only

"the other thing is practice awareness, you know, because you said it's a big practice and if somebody comes in and hasn't got the sort of clock-in bit straight and they go to the desk and say, I've come to see X about this trial and, you know, new receptionist knows nothing. That can be quite off-putting so as long as everybody's engaged …and everybody knows about it".

Research nurse interview 2

For peer review only

Figure 1 Questionnaire design features reported by interviewees to improve questionnaire return

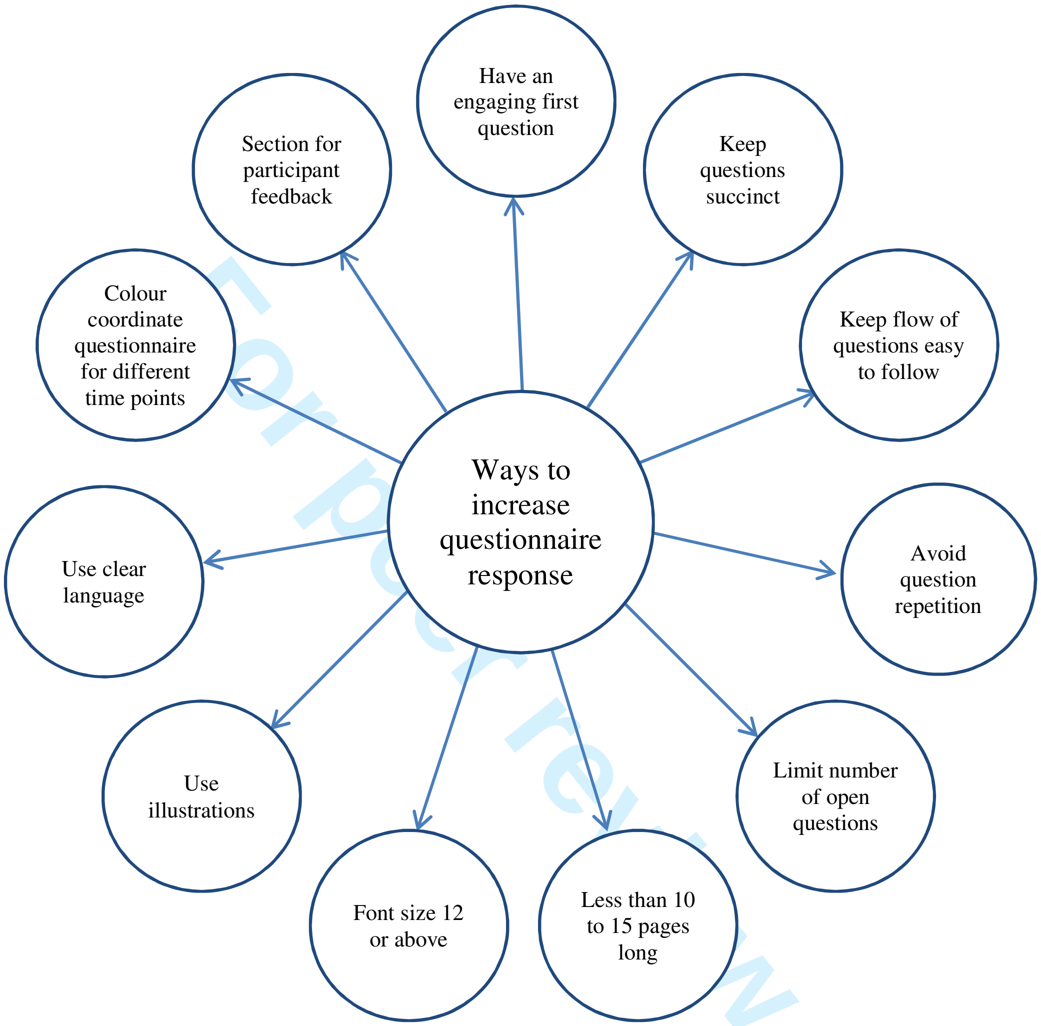

Web Appendix 1: Interview schedule

Background / introduction to the interview

I am interested in attrition from randomised trials. We define attrition as incomplete ascertainment of the primary outcome, but will subsequently use the term "loss to follow-up". I want to explore trialist's opinions on if and when loss to follow-up has been a problem in their experience and the strategies they may have used to deal with or to prevent loss to follow-up. I also want to find out more about trialists preferred strategies for preventing loss to follow-up.

Topic guide questions

1. **Can you tell me about recent trials you have worked on?**

    Were there any with high follow-up?

    Were there any with low follow-up?

2. **What do you think are the factors that lead to loss to follow-up in trials?**

    Why do you think it can be difficult to keep participants in randomised trials?

3. **What are the factors that lead to retention in randomised trials?**

4. **What strategies to increase follow-up have been successful for you in trials you have worked on?**

    Why do you think these have worked?

    What strategies have been unsuccessful?

    Why have these not worked?

Decision making around strategies used to reduce loss to follow-up

1. When do you perceive loss to follow-up to be a problem?
2. How do you decide which strategies to improve follow-up work best?
3. How did/ do you implement the strategies to improve follow-up that worked best?
4. How is loss to follow-up monitored in trials you have worked on?
5. Who deals with loss to follow-up when it presents?

Impact of research governance

1. What do you feel about using incentives to keep participants in trials?
2. Have you had any experience with ethics committees?
3. What do you feel about ethics committees asking about payments or giving other incentives to participants?
4. Has ethics committees approach to payments affected any trials you have worked on in anyway?

Ask this next question at the end of the interview

These are the strategies to reduce attrition/ improve retention identified by the Cochrane review.

For peer review only

Show participant each card separately (See below). Then ask the following questions for each strategy discussed:

1. Have you used these?
2. What do you think about using this strategy?
3. Might you have considered using this strategy in your trial?
4. What could be the advantages of using the strategy?
5. What could be the disadvantages of using this strategy?

Each card shown individually

Card no 1

Communication strategies. e.g. email, telephone, text messages, letters signed by different study personnel, type of delivery- e.g. post 1st 2nd class, or recorded delivery, type of envelope used for response.

Card no 2

Incentives to either participants or trialists e.g. gifts pens, pins, monetary incentives, offers of incentives, vouchers.

Card no 3

Methodological strategies blind versus un blind trials.

Card no 4

Different length of questionnaire: Short versus long.

Card no 5

Using case management. Having trial assistants manage participant follow-up, for example arranging transport and services to enable participants to keep trial follow-up appointments.

Card no 6

Motivational/educational strategies. Such as arranging workshops to give participants information about goal setting and time management.

For peer review only
